# Burden of Skin Cancer in Older Adults From 1990 to 2021 and Modelled Projection to 2050

**DOI:** 10.1001/jamadermatol.2025.1276

**Published:** 2025-05-21

**Authors:** Ruiyao Wang, Yangmei Chen, Xinyi Shao, Tingqiao Chen, Judan Zhong, Yi Ou, Jin Chen

**Affiliations:** 1 Department of Dermatology, The First Affiliated Hospital of Chongqing Medical University, Chongqing, China

## Abstract

**Importance:**

Skin cancer is a significant public health threat in the aging population, yet data on its burden and trends are limited.

**Objective:**

To evaluate the global burden of skin cancer among adults 65 years or older from 1990 to 2021, and to project its change to 2050.

**Design, Setting, and Participants:**

In this observational study, population-based registry data were extracted from the Global Burden of Diseases (GBD) Study 2021. The database covers 204 countries and territories, ensuring global applicability of the results. Data analysis was conducted from June 9, 2024, to February 18, 2025.

**Main Outcomes and Measures:**

Age-standardized rates of prevalence, incidence, deaths, and disability-adjusted life-years (DALYs) were calculated per 100 000 population. Estimated rates of disease burden were calculated for 2050.

**Results:**

A worldwide total of 153 993 melanoma, 1 463 424 squamous cell carcinoma (SCC), and 2 802 354 basal cell carcinoma (BCC) instances were estimated for 2021. SCC exhibited the highest age-standardized rate of prevalence (236.91 per 100 000 population; 95% uncertainty interval [UI], 188.23-303.82), deaths (6.16 per 100 000 population; 95% UI, 5.13-6.87), and DALYs (95.50 per 100 000 population; 95% UI, 81.65-106.39), whereas BCC displayed the highest incidence rate (371.97 per 100 000 population; 95% UI, 310.75-439.58). The disease burden was notably greater in male individuals than in female individuals. During the observation period, the global burden of skin cancer among older people demonstrated a general upward trend. Decomposition analysis indicated that population growth was the primary contributor to this increase. Health-inequality analysis revealed a disproportionately higher burden shouldered by countries with higher sociodemographic index (SDI) levels. Frontier analysis identified the countries with considerable potential to mitigate skin cancer. It was anticipated that only incidence and prevalence rates attributable to keratinocyte cancer (KC), along with DALYs rate related to BCC, would increase by 2050.

**Conclusions and Relevance:**

The older population (particularly male individuals and those living in high-SDI countries) is facing a substantial growing burden of skin cancer. Despite the relative incompleteness of KC data and lack of race and ethnicity data, these results highlight the urgency for more effective prevention and management strategies targeting high-risk groups.

## Key Points

**Question:**

What is the global burden of skin cancer among older people, and how has it changed from 1990 to 2021?

**Findings:**

In this study using data from the Global Burden of Diseases database, there was a significant increase in skin cancer burden over the past 3 decades (particularly for keratinocyte cancer) driven by population growth and marked by disparities in sex and sociodemographic index. Projections suggest a rising incidence rate for keratinocyte cancer and decreasing burden of cutaneous melanoma by 2050.

**Meaning:**

These findings highlight the urgent need for targeted prevention strategies and resource allocation to address the growing public health challenge of skin cancer among the aging population.

## Teaser

This study using data from the Global Burden of Diseases database investigates the most updated trends in skin cancer burden across subgroups of age cohorts, sexes, geographical regions, and sociodemographic index levels and further models a predicted burden by 2050.

## Introduction

Skin cancer is the most prevalent type of cancer. It can be classified into cutaneous melanoma and keratinocyte cancer (KC). Within KC, basal cell carcinoma (BCC) and squamous cell carcinoma (SCC) are the most important from epidemiological and clinical perspectives.^1^ Skin cancer ranks among the costliest cancer to treat in the US,^2^ exerting substantial health and economic burdens.^3^

The global population is aging. This has resulted in a shift in the epidemiologic profile toward the predominance of chronic, nontransmissible diseases, with cancer being one of them.^4^ An observational study^5^ in Japan revealed that the proportion of patients with skin cancer older than 70 years increased from 44% in 1989 to 74% in 2021. Most cutaneous malignant diseases occur in the older population, which is associated with higher incidence and mortality rates.^6^ Age-related disparities in the clinical management of melanoma are considerable, often resulting in undertreatment and restricted access to advanced surgical and medical interventions, particularly among older patients.^7^

The latest spatial patterns and temporal trends of skin cancer burden among the older population, which is essential for targeted public policymaking, are lacking. We aim to investigate the most updated trends in skin cancer burden across subgroups of age cohorts, sexes, geographical regions, and sociodemographic index (SDI) level and further model a predicted burden by 2050.

## Methods

### Data Source

Data on skin cancer from 1990 to 2021 were sourced from the Global Burden of Disease (GBD) Study 2021 database, which is accessible on the Institute for Health Metrics and Evaluation website.^8^ Data collection and analysis followed the Strengthening the Reporting of Observational Studies in Epidemiology (STROBE) reporting guidelines. We undertook a systematic analysis of the incidence, prevalence, deaths, and disability-adjusted life-years (DALYs) associated with skin cancer among adults 65 years or older. We examined these metrics at global, regional, and national levels. DALYs, which are quantified as the aggregate of years of life lost and years lived with disability, serve as a comprehensive metric for assessing the overall burden of a disease/health condition.^9,10,11^ We also used the SDI, a composite measure that quantifies sociodemographic status of a country or region based on its average income, educational attainment, and fertility rates.^12,13^ Ethical approval was waived because the analysis was deemed nonhuman participants research by the First Affiliated Hospital of Chongqing Medical University, and informed consent was not required.

### Statistical Analysis

Data are numerical counts and age-standardized rates (ASRs) per 100 000 population, accompanied by 95% uncertainty intervals (UIs), which were based on the 2.5 and 97.5 ordered values of 1000 draws of the posterior distribution.^14,15^ We used joinpoint regression analysis to assess the temporal trends in ASRs of skin cancer burden at global, regional, and national levels. This method identifies points with significant changes in trends (ie, joinpoints), divides the overall trend into multiple subsegments based on the observed joinpoints, and assesses the epidemiological trend of each subsegment by calculating the annual percentage change (APC) and average APC (AAPC).^16^ Decomposition analysis was undertaken to elucidate the separate contribution of 3 population-level drivers: population aging, population growth, and age- and population-standardized rates (referred to as *epidemiologic changes*) to the total change of skin cancer burden.^17,18^ This methodology entailed assessing the contribution of each factor independently while holding the other 2 factors fixed, thereby determining the extent to which epidemiological trends were affected by each specific factor.^19,20^

Inequalities analysis was also used. The inequality slope index and concentration index are standardized indicators for measuring absolute and relative gradient inequalities, respectively.^21,22^ The inequality slope index was determined through regression analysis, correlating the country-level DALYs rate with its SDI relative position, defined by the midpoint of the population in a cumulative distribution ranked by SDI.^23,24^ The concentration index was derived by fitting a Lorenz concentration curve to the observed cumulative relative distribution of the populations ranked by SDI and the corresponding DALYs of the disease, as well as numerically integrating the area under the curve.^25,26^ Frontier analysis was used to (1) evaluate the potentially achievable age-standardized DALYs on the basis of a given SDI; and (2) identify countries for which performance was lagging or leading relative to other countries with the same level of development.^18,27^

Furthermore, we used the Bayesian age–period–cohort (BAPC) model implemented in the integrated nested laplace approximations (INLA) framework to predict future trends by 2050.^28,29^

The detailed analysis methods are presented in the eMethods in Supplement 1. All procedures for analysis and graphic representation were undertaken utilizing R statistical software (version 4.3.2; R Institute for Statistical Computing). World maps were created with QGIS software (version 3.34.4; QGIS Development Team).

## Results

### Global Trends

Among individuals 65 years or older, the global numbers of new reported cases for melanoma, SCC, and BCC were 153 993, 1 463 424, and 2 802 354, respectively, in 2021 (eTables 1-3 in Supplement 1). BCC had the highest age-standardized incidence rate (371.97 per 100 000 population; 95% UI, 310.75-439.58), which was 1.9-fold of the incidence rate of SCC (196.24 per 100 000 population; 95% UI, 163.32-234.39). SCC accounted for the highest number of DALYs (approximately 706 405), which corresponded to an age-standardized DALYs rate of 95.50 per 100 000 population (95% UI, 81.65-106.39). The incidence and prevalence rate of melanoma across all age subgroups increased during 1990 to 2021, especially among those aged 85 to 94 years (Table 1). The mortality rates for melanoma per 100 000 population among people 65 years or older increased with age, from 2.5 among those aged 65 to 69 years to 21.28 among those 95 years or older. The mortality and DALYs of melanoma decreased among those 79 years or younger worldwide, especially among those aged 65 to 69 years. KC burden showed an upward trend during the observation period. KC rates increased with age, with the highest rates among those 95 years or older (Table 2 and Table 3). The DALYs rate for SCC per 100 000 population increased from 56 among those aged 65 to 69 years (56.33; 95% UI, 47.78-63.13) to 485 among those 95 years or older (485.05; 95% UI, 362.12-549.80). Within each age cohort, BCC exhibited the highest absolute numbers and incidence rates, followed by SCC and melanoma. Joinpoint analysis indicated a significant increase in the global burden of skin cancer from 1990 to 2021, as evidenced by the AAPCs exceeding zero (eTables 1-3 in Supplement 1). Importantly, the ASRs of deaths and DALYs attributable to melanoma exhibited relative stability during this period. There were disparities between the sexes, with higher numbers and rates observed in men. Notably, male individuals (ASR = 286.89; 95% UI, 238.37-343.30) had an almost 230% higher SCC-related incidence rate than female individuals (ASR = 128.29; 128.29; 95% UI, 106.66-153.44). The gender gap has expanded slightly over recent decades, primarily due to a more pronounced increased incidence among male individuals. An ascending trend was observed in the ASRs of incidence and prevalence as the SDI increased. Countries with a high SDI level exhibited the highest ASRs of deaths and DALYs due to melanoma, with rates of 9.49 per 100 000 population (95% UI, 8.38-10.13) and 175.6 per 100 000 population (95% UI, 157.91-189.12), respectively. These rates were more than 5-fold higher than those recorded in countries with a low-middle to middle SDI levels.

**Table 1.** Global Burden of Cutaneous Melanoma by Age Group in 2021 and Average Annual Percentage Change (AAPC) From 1990 to 2021

Age group, y	Incidence			Prevalence			Deaths			DALYs		
	No. (95% UI)	Age-specific rate per 100 000 (95% UI)	AAPC (95% CI)	No. (95% UI)	Age-specific rate per 100 000 (95%UI)	AAPC (95%CI)	No. (95% UI)	Age-specific rate per 100 000 (95% UI)	AAPC (95% CI)	No. (95%UI)	Age-specific rate per 100 000 (95% UI)	AAPC (95% CI)
65-69	35 842 (33 664 to 37 785)	12.99 (12.20 to 13.70)	0.65 (0.55 to 0.65)	267 257 (252 534 to 281 507)	96.89 (91.55 to 102.05)	1.14 (1.08 to 1.19)	6893 (6139 to 7391)	2.50 (2.23 to 2.68)	−0.68 (−0.71 to −0.65)	183 671 (165 601 to 198 338)	66.59 (60.03 to 71.90)	−0.58 (−0.61 to −0.55)
70-74	37 586 (34 756 to 39 674)	18.26 (16.88 to 19.27)	1.12 (1.04 to 1.17)	269 793 (251 099 to 283 812)	131.07 (121.99 to 137.88)	1.67 (1.58 to 1.75)	7557 (6846 to 8075)	3.67 (3.33 to 3.92)	−0.22 (−0.29 to −0.16)	167 358 (151 764 to 180 374)	81.31 (73.73 to 87.63)	−0.1 (−0.16 to −0.04)
75-79	29 704 (26 879 to 31 632)	22.52 (20.38 to 23.98)	1.21 (1.15 to 1.25)	197 833 (177 835 to 209 819)	150.00 (134.84 to 159.09)	2.09 (2.04 to 2.14)	6930 (6219 to 7419)	5.25 (4.72 to 5.63)	−0.23 (−0.27 to −0.21)	123 110 (111 355 to 133 213)	93.35 (84.43 to 101.01)	−0.09 (−0.13 to −0.06)
80-84	24 780 (21 022 to 27 139)	28.29 (24.00 to 30.99)	1.57 (1.51 to 1.64)	144 079 (119 371 to 157 729)	164.51 (136.29 to 180.09)	2.70 (2.62 to 2.79)	6955 (5965 to 7563)	7.94 (6.81 to 8.64)	0.23 (0.17 to 0.28)	96 690 (83 613 to 105 776)	110.40 (95.47 to 120.77)	0.36 (0.31 to 0.41)
85-89	17 124 (13 705 to 19 025)	37.45 (29.97 to 41.61)	1.74 (1.67 to 1.81)	82 690 (64 927 to 92 724)	180.86 (142 to 202.8)	3.18 (3.09 to 3.27)	5260 (4377 to 5817)	11.51 (9.57 to 12.72)	0.42 (0.38 to 0.46)	58 454 (48 503 to 64 929)	127.85 (106.08 to 142.01)	0.57 (0.52 to 0.61)
90-94	6768 (5345 to 7610)	37.83 (29.88 to 42.54)	1.71 (1.63 to 1.79)	19 842 (15 336 to 22 525)	110.92 (85.73 to 125.91)	3.87 (3.78 to 3.97)	3017 (2405 to 3365)	16.87 (13.44 to 18.81)	0.54 (0.50 to 0.60)	28 082 (22 377 to 31 359)	156.97 (125.08 to 175.29)	0.65 (0.60 to 0.70)
≥95	2188 (1606 to 2513)	40.15 (29.46 to 46.10)	1.64 (1.56 to 1.71)	1523 (1110 to 1754)	27.95 (20.37 to 32.19)	2.42 (2.28 to 2.54)	1160 (848 to 1319)	21.28 (15.56 to 24.21)	0.66 (0.60 to 0.71)	9838 (7221 to 11 186)	180.51 (132.48 to 205.24)	0.69 (0.63 to 0.75)

Abbreviations: DALYs, disability-adjusted life-year; UI, uncertainty interval.

**Table 2.** Global Burden of Squamous Cell Carcinoma by Age Group in 2021 and Average Annual Percentage Change (AAPC) From 1990 to 2021

Age group, y	Incidence			Prevalence			Deaths			DALYs		
	No. (95% UI)	Age-specific rate per 100 000 (95%UI)	AAPC (95% CI)	No. (95% UI)	Age-specific rate per 100 000 (95%UI)	AAPC (95% CI)	No. (95% UI)	Age-specific rate per 100 000 (95%UI)	AAPC (95% CI)	No. (95% UI)	Age-specific rate per 100 000 (95%U)	AAPC (95% CI)
65-69	303 767 (258 514 to 352 052)	110.12 (93.72 to 127.63)	2.03 (1.91 to 2.16)	35 4463 (28 5583 to 442 283)	128.50 (103.53 to 160.34)	2.36 (2.20 to 2.49)	5787 (4901 to 6564)	2.10 (1.78 to 2.38)	0.01 (−0.01 to 0.04)	155 378 (131 811 to 174 148)	56.33 (47.78 to 63.13)	0.15 (0.12 to 0.17)
70-74	356 664 (296 845 to 428 785)	173.27 (144.21 to 208.31)	2.03 (1.92 to 2.13)	425 949 (338 490 to 535 409)	206.93 (164.44 to 260.11)	2.33 (2.23 to 2.44)	6811 (5782 to 7660)	3.31 (2.81 to 3.72)	0.13 (0.11 to 0.15)	153 534 (132 226 to 171 595)	74.59 (64.24 to 83.36)	0.32 (0.29 to 0.35)
75-79	300 351 (243 608 to 367 804)	227.74 (184.71 to 278.88)	1.94 (1.84 to 2.05)	363 660 (284 888 to 478 964)	275.74 (216.01 to 363.17)	2.26 (2.13 to 2.40)	6791 (5922 to 7460)	5.15 (4.49 to 5.66)	0.11 (0.08 to 0.12)	122 833 (108 937 to 136 654)	93.14 (82.6 to 103.62)	0.29 (0.26 to 0.31)
80-84	234 242 (201 431 to 27 5068)	267.45 (229.99 to 314.07)	1.35 (1.22 to 1.49)	286 826 (226 270 to 374 338)	327.49 (258.35 to 427.41)	1.73 (1.59 to 1.88)	7964 (6759 to 8796)	9.09 (7.72 to 10.04)	0.23 (0.20 to 0.26)	110 362 (96 153 to 121 556)	126.01 (109.78 to 138.79)	0.31 (0.28 to 0.34)
85-89	156 262 (131 550 to 182 310)	341.77 (287.72 to 398.74)	0.71 (0.63 to 0.80)	192 688 (159 409 to 241 458)	421.44 (348.65 to 528.10)	1.03 (0.93 to 1.12)	7851 (6493 to 8697)	17.17 (14.20 to 19.02)	0.27 (0.23 to 0.31)	84 921 (72 204 to 94 196)	185.73 (157.92 to 206.02)	0.30 (0.26 to 0.33)
90-94	81 919 (64 772 to 101 247)	457.92 (362.07 to 565.96)	0.28 (0.19 to 0.37)	101 757 (79 819 to 132 266)	568.81 (446.18 to 739.35)	0.63 (0.54 to 0.72)	5710 (4611 to 6356)	31.92 (25.78 to 35.53)	0.52 (0.48 to 0.56)	52 940 (43 627 to 58 988)	295.93 (243.87 to 329.74)	0.53 (0.49 to 0.56)
≥95	30220 (22436 to 39 250)	554.47 (411.65 to 720.15)	−0.36 (−0.48 to −0.26)	38 342 (27 473 to 53 979)	703.48 (504.05 to 990.38)	−0.07 (−0.19 to 0.04)	3109 (2277 to 3538)	57.05 (41.78 to 64.91)	0.90 (0.83 to 0.96)	26 437 (19 736 to 29 966)	485.05 (362.12 to 549.80)	0.82 (0.78 to 0.87)

Abbreviations: DALYs, disability-adjusted life-year; UI, uncertainty interval.

**Table 3.** Global Burden of Basal Cell Carcinoma by Age Group in 2021 and Average Annual Percentage Change (AAPC) From 1990 to 2021

Age group, y	Incidence			Prevalence			DALYs		
	No. (95% UI)	Age-specific rate per 100 000 (95% UI)	AAPC (95% CI)	No. (95% UI)	Age-specific rate per 100 000 (95% UI)	AAPC (95% CI)	No. (95% UI)	Age-specific rate per 100 000 (95% UI)	AAPC (95% CI)
65-69	677 074 (579 145 to 776 585)	245.46 (209.96 to 281.53)	1.79 (1.68 to 1.88)	71 504 (59 727 to 86 646)	25.92 (21.65 to 31.41)	1.55 (1.45 to 1.63)	299 (139 to 566)	0.11 (0.05 to 0.21)	1.54 (1.44 to 1.62)
70-74	719 050 (60 4243 to 840 635)	349.33 (293.55 to 408.39)	1.71 (1.63 to 1.77)	76 085 (62 903 to 92 592)	36.96 (30.56 to 44.98)	1.56 (1.44 to 1.62)	311 (142 to 595)	0.15 (0.07 to 0.29)	1.52 (1.42 to 1.58)
75-79	579 513 (476 035 to 693 808)	439.41 (360.95 to 526.07)	1.62 (1.54 to 1.67)	61 794 (48 311 to 76 991)	46.85 (36.63 to 58.38)	1.26 (1.18 to 1.32)	247 (109 to 487)	0.19 (0.08 to 0.37)	1.22 (1.15 to 1.28)
80-84	421 524 (353 510 to 490 123)	481.28 (403.63 to 559.61)	1.20 (1.11 to 1.27)	46 230 (36 365 to 57 035)	52.78 (41.52 to 65.12)	0.90 (0.82 to 0.96)	182 (80 to 363)	0.21 (0.09 to 0.41)	0.85 (0.78 to 0.91)
85-89	248 491 (210 940 to 298 201)	543.48 (461.36 to 652.21)	0.86 (0.79 to 0.93)	27 305 (22 148 to 32 962)	59.72 (48.44 to 72.09)	0.64 (0.57 to 0.71)	105 (49 to 200)	0.23 (0.11 to 0.44)	0.59 (0.52 to 0.66)
90-94	116 883 (92 548 to 150 455)	653.36 (517.34 to 841.03)	0.68 (0.62 to 0.73)	12 841 (9690 to 16 724)	71.78 (54.16 to 93.49)	0.55 (0.48 to 0.60)	49 (22 to 91)	0.27 (0.12 to 0.51)	0.50 (0.45 to 0.55)
≥95	39 820 (27 837 to 55 203)	730.61 (510.73 to 1012.84)	0.28 (0.22 to 0.34)	4410 (2871 to 6353)	80.91 (52.67 to 116.57)	0.13 (0.05 to 0.19)	16 (7 to 32)	0.30 (0.12 to 0.58)	0.10 (0.02 to 0.17)

Abbreviations: DALYs, disability-adjusted life-year; UI, uncertainty interval.

### Regional and National Trends

The world regions with the greatest melanoma-related burden were Australasia, North America, Western Europe, Central Europe, and Eastern Europe. The highest ASRs of incidence (158.10; 95% UI, 123.16-197.66), prevalence (1165.26; 95% UI, 917.05-1458.33), deaths (27.83; 95% UI, 22.79-32.97), and DALYs (502.22; 95% UI, 412.98-598.92) were observed in Australasia. Australasia experienced the most significant decline in mortality and DALYs attributable to melanoma, with an average annual trend of −1.10% (eTable 1 in Supplement 1). Among the 21 regions, high-income North America reported the highest ASRs of incidence and prevalence attributable to KC, with these rates significantly surpassing global average ASRs (eTables 2-3 in Supplement 1). The highest ASRs of deaths (15.37; 95% UI, 12.80-17.36) and DALYs (226.92; 95% UI, 193.43-256.48) associated with SCC were seen in Australasia, whereas the highest rate of DALYs for BCC (1.21; 95% UI, 0.56-2.36) was recorded in high-income North America. East Asia demonstrated the most rapid escalation in BCC metrics, with all AAPCs exceeding 6%.

At the national level in 2021, New Zealand had the highest burden of melanoma among the aging population, with the ASRs of incidence, prevalence, deaths, and DALYs (per 100 000 population) recorded at 202.21 (95% UI, 152.23-262.44), 1495.67 (95% UI, 1127.78-1935.18), 30.49 (95% UI, 24.55-36.8), and 560.61 (95% UI, 452.38-680.46), respectively (Figure; eFigures 1-3 in Supplement 1). Over the observation period, Mauritius exhibited the most significant increase in rates of incidence, deaths, and DALYs related with melanoma, with AAPCs greater than 7%. Qatar reported the most pronounced decrease in rates of deaths and DALYs (eTable 4 in Supplement 1). Notable variations in SCC burden were observed across 204 countries, with the US registering the highest incidence rate, followed by New Zealand and Australia (eTable 5 in Supplement 1). China showed the most substantial increase in incidence rate among older individuals with SCC, with AAPC of 6.02% (95% UI, 5.67%-6.37%). Regarding ASRs of deaths and DALYs attributable to SCC, Georgia demonstrated the highest annual increase, whereas Poland showed the most pronounced decrease. The US led in the burden metrics of BCC, reaching (per 100 000 population) an incidence rate of 3452.24 (95% UI, 2971.67-3949.03), prevalence rate at 343.35 (95% UI, 285.91-413.48), and DALYs rate at 1.35 (95% UI, 0.62-2.62) (eTable 6 in Supplement 1). For BCC burden in China during this timeframe, the AAPC of incidence rate was 6.86 (95% UI, 6.46-7.25). The AAPC of the prevalence rate was 6.35 (95% UI, 5.99-6.72) and the AAPC of DALYs rate was 6.27 (95% UI, 5.92-6.63).

**Figure.**
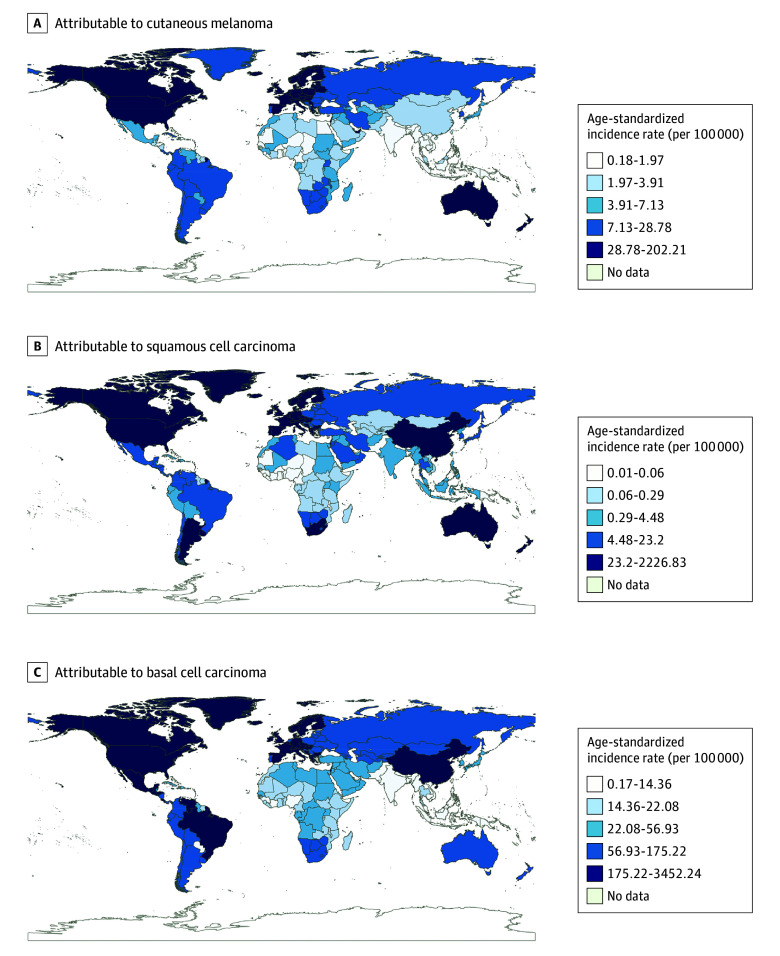
Global Maps of Age-Standardized Incidence Rate Global maps of age-standardized incidence rate attributable to (A) cutaneous melanoma, (B) squamous cell carcinoma, and (C) basal cell carcinoma in 2021.

### Decomposition Analysis of Change in Incidence and DALYs

The findings revealed that population growth emerged as a principal contributor to the rising disease burden (eFigure 4 in Supplement 1). This effect was most pronounced in the low-middle SDI quintile, where population growth accounted for a 139.15% increase in new cases of BCC (eTable 7 in Supplement 1). In terms of epidemiological changes, population growth was associated with an adverse effect on the incidence of KC in low and low-middle SDI regions and DALYs of melanoma in low SDI regions (eTable 8 in Supplement 1).

### Cross-Country Inequality Analysis

Significant absolute and relative SDI-related inequalities were identified, with a disproportionate greater burden shouldered by countries with higher SDI (eFigure 5 in Supplement 1). As illustrated by the slope index of inequality, the gap of DALYs attributable to melanoma between countries with the highest and lowest SDI increased from 124.50 (95% CI, 96.77-152.24) in 1990 to 144.82 (95% CI, 115.81-173.83) in 2021. The slope index of DALYs due to SCC in 1990 was 64.97 (95% CI, 45.57-84.37), so there was an excess of 64.97 (per 100 000 population) DALYs in the country with the highest SDI compared with that in the country with the lowest SDI in 1990, and this gap narrowed to 35.64 (95% UI, 17.34- 53.93) in 2021. For BCC, the slope index of DALYs showed a slight reduction from 0.11 (95% CI, 0.09-0.13) in 1990 to 0.10 (95% CI, 0.09-0.12) in 2021. Meanwhile, the concentration index indicated an upward trend or relative stability from 1990 to 2021, suggesting persistent inequalities.

### Frontier Analysis for the Association Between Ideal Cancer DALYs and SDI

The frontier line delineated the countries with lowest DALY rates (optimal performers) given their SDI. Distance from the frontier line was termed *effective difference* and represented the gap between the observed and potentially achievable DALYs of a country (eFigure 6, eTables 9-11 in Supplement 1).

In terms of DALYs attributable to melanoma, the countries furthest from the frontier line were New Zealand, Australia, Norway, North Macedonia, and Slovenia. Regarding DALYs associated with SCC, the countries furthest from the frontier line were Georgia, Tonga, New Zealand, Cuba, and Australia. For DALYs linked to BCC, the countries furthest from the frontier line were the US, Greenland, Sweden, Switzerland, and Ireland. These countries exhibited significantly higher rates of skin cancer DALYs in relation to other countries with comparable sociodemographic profiles.

### Projections of Skin Cancer up to 2050

Based on the INLA framework and BAPC methodology, we predicted the global disease burden by 2050 (eFigure 7-9 in Supplement 1). At the global scale, projections indicated a decline in melanoma metrics, with the age-standardized prevalence rate expected to decrease by up to 45.87%. The age-standardized incidence and prevalence rates of KC were projected to exhibit an upward trend. Specifically, the incidence rate of newly diagnosed BCC was anticipated to increase by more than 140%. Our findings indicated that the SCC-related rates of deaths and DALYs would decrease. Conversely, the model forecasted a 43.75% increase in the age-standardized DALYs rate of BCC.

## Discussion

This observational study is the first to our knowledge to provide a comprehensive examination of the epidemiological trends in the disease burden posed by skin cancer among older people, incorporating sex, age, and the SDI. In 2021, skin cancer constituted a substantial cancer burden in individuals 65 years or older, predominantly affecting high SDI regions and male individuals. Despite the relative stability in ASRs of deaths and DALYs attributable to melanoma, the global burden of skin cancer increased continuously over this 3-decade span. Decomposition analysis revealed that population growth was the primary driver of this increasing burden. A persistent disproportionate disease burden was observed in regions with high SDI levels. Frontier analysis pinpointed the countries that must prioritize addressing the skin cancer burden. The BAPC model projected that by 2050 there will be an increase in the rates of incidence and prevalence associated with KC, as well as an increase in the DALYs rate of BCC.

The ASRs of skin cancer in the older population were significantly higher compared with the general population, as reported previously.^30,31^ A global upward trend in the ASRs of incidence and prevalence was observed during the study period. The increased disease-burden metrics observed in older individuals are likely the result of prolonged lifetime cumulative risk factors, such as the accumulation of intermittent sun exposure beginning in adolescence.^32,33^ An escalation in screening and biopsy procedures, leading to possible diagnoses of negligible or dormant disease, may also contribute to its reported sharp increase in incidence.^34,35^ Also, the efficacy of cancer reporting systems may also be related to the changing trends.^36^

Sex differences were observed in the global distribution of skin cancer. Over recent decades, there was a more pronounced increase in all burden metrics among male individuals, corresponding well with the literature.^37,38^ The higher incidence of melanoma in men may be due to less use of sun protection and greater exposure to outdoor environments, whether for occupational or recreational purposes, compared with women.^39^ Studies have shown that men have lower awareness of skin cancer, often neglecting the importance of regular skin examinations and early screenings.^40^ Despite higher prevalence rates, female patients with melanoma may experience more favorable outcomes because estrogen likely stimulates immune responses by blocking the inhibitory signals that prevent tumor recognition.^41^

Countries experiencing the greatest burden of skin cancer included Australia, New Zealand, and the US, consistent with previous studies.^1,42^ Countries possessing a higher SDI level disproportionately reported greater burden. Increased incidence and prevalence rates of melanoma were observed in countries with high SDI levels, which could be attributed to the accessibility of medical resources, environmental factors, and lifestyle. Exposure to UV light is the most significant modifiable risk factor for melanoma.^43,44^ Changes in leisure-time activities, including extended sun exposure and the use of indoor tanning facilities, have resulted in a substantial rise in exposure to UV light, thereby contributing to the increased incidence of melanoma.

We predicted that the rates of incidence and prevalence attributable to KC, as well as DALY rates related to BCC, may continue to rise by 2050, whereas the deaths and DALY rates of SCC and the burden of melanoma may exhibit a decreasing trend. The age-standardized prevalence rate of melanoma was expected to decrease by 45% by 2050; this could be partly attributable to the introduction of effective systemic therapies for melanoma.^45^ This decade has brought considerable survival improvement for patients thanks to targeted therapies and immunotherapies.^46,47^ In line with previous studies,^48^ KC will cause more deaths than melanoma globally based on current trends. By 2050, 80% of the older population will be living in low- and middle-income countries.^49^ With an increasing older population and the corresponding rise in skin cancer incidence, we believe an emphasis on skin cancer prevention could lead to more sustainable interventions with favorable outcomes. Sun protection is an essential component to the primary prevention of malignant skin diseases. Counseling patients from a young age on the importance of sun-safe behaviors could mitigate the cumulative effects of sun exposure. Photoprotection remains beneficial even in older populations with cumulative UV exposure for reducing the risk of new skin lesions.

Skin cancer in older patients is likely to be diagnosed at a more advanced stage,^50^ which complicates treatment due to age-related declines in physical and cognitive function, as well as the need for social care.^51^ Our findings suggest that closer collaboration between geriatricians and dermatologists is crucial for optimizing treatment. In addition, enhancing community engagement and preventive strategies could help address the challenges of skin cancer in very old populations.

### Limitations

Our data have limitations that need to be considered when interpreting the study findings. Studies measuring KC are limited because of their exclusion from some large cancer registries, which hinders data comparison. National estimates of nonstandard countries with sparse high-quality data may be misleading because the results in those locations were obtained by mathematical modeling using the available coefficients of their parent locations in the GBD 2021. Specifically, data on race and ethnicity are not available in GBD, which limits the assessment of its confounding effects on skin cancer burden across different SDI countries.

## Conclusions

The findings of this study suggest that the global disease burden of skin cancer in adults 65 years or older is on the rise, particularly among male individuals and in countries with a high SDI level. Our results underscore the urgency to enact prevention and treatment strategies tailored to high-risk older populations.

## Notes

Supplement 1. eMethods eTable 1. Global burden of cutaneous melanoma among adults aged ≥65 years in 2021, categorized by sex, region, and SDI eTable 2. Global burden of squamous cell carcinoma among adults aged ≥65 years in 2021, categorized by sex, region, and SDI eTable 3. Global burden of basal cell carcinoma among adults aged ≥65 years in 2021, categorized by sex, region, and SDI eTable 4. Burden of cutaneous melanoma in 204 countries and territories in 2021 and the average annual percent change (AAPC) from 1990 to 2021 eTable 5. Burden of squamous cell carcinoma in 204 countries and territories in 2021 and the average annual percent change (AAPC) from 1990 to 2021 eTable 6. Burden of basal cell carcinoma in 204 countries and territories in 2021 and the average annual percent change (AAPC) from 1990 to 2021 eTable 7. Decomposition of change in incidence globally and by SDI quintile, 1990 to 2021 eTable 8. Decomposition of change in DALYs globally and by SDI quintile, 1990 to 2021 eTable 9. Frontier analysis based on SDI and DALYs of cutaneous melanoma in 204 countries and territories eTable 10. Frontier analysis based on SDI and DALYs of squamous cell carcinoma in 204 countries and territories eTable 11. Frontier analysis based on SDI and DALYs of basal cell carcinoma in 204 countries and territories eFigure 1. Global maps of age-standardised prevalence rate attributable to skin cancer in 2021 eFigure 2. Global maps of age-standardised deaths rate attributable to skin cancer in 2021 eFigure 3. Global maps of age-standardised disability-adjusted life-years (DALYs) rate attributable to skin cancer in 2021 eFigure 4. Decomposition analysis of changes in incidence and DALYs attributable to skin cancer, 1990-2021 eFigure 5. Inequality analysis of DALYs attributable to skin cancer, 1990-2021 eFigure 6. Frontier analysis based on DALYs attributable to skin cancer and SDI, 1990-2021 eFigure 7. Projected burden of cutaneous melanoma by 2050 eFigure 8. Projected burden of squamous cell carcinoma by 2050 eFigure 9. Projected burden of basal cell carcinoma by 2050

Supplement 2. Data Sharing Statement
