# Supplementary material for: Burden of Skin Cancer in Older Adults From 1990 to 2021 and Modelled Projection to 2050
Source: JAMA Dermatol. 2025 May 21;161(7):715–22. doi: 10.1001/jamadermatol.2025.1276 (PMC12096324; doi:10.1001/jamadermatol.2025.1276)
Supplement: Supplement 1. — eMethods eTable 1. Global burden of cutaneous melanoma among adults aged ≥65 years in 2021, categorized by sex, region, and SDI eTable 2. Global burden of squamous cell carcinoma among adults aged ≥65 years in 2021, categorized by sex, region, and SDI eTable 3. Global burden of basal cell carcinoma among adults aged ≥65 years in 2021, categorized by sex, region, and SDI eTable 4. Burden of cutaneous melanoma in 204 countries and territories in 2021 and the average annual percent change (AAPC) from 1990 to 2021 eTable 5. Burden of squamous cell carcinoma in 204 countries and territories in 2021 and the average annual percent change (AAPC) from 1990 to 2021 eTable 6. Burden of basal cell carcinoma in 204 countries and territories in 2021 and the average annual percent change (AAPC) from 1990 to 2021 eTable 7. Decomposition of change in incidence globally and by SDI quintile, 1990 to 2021 eTable 8. Decomposition of change in DALYs globally and by SDI quintile, 1990 to 2021 eTable 9. Frontier analysis based on SDI and DALYs of cutaneous melanoma in 204 countries and territories eTable 10. Frontier analysis based on SDI and DALYs of squamous cell carcinoma in 204 countries and territories eTable 11. Frontier analysis based on SDI and DALYs of basal cell carcinoma in 204 countries and territories eFigure 1. Global maps of age-standardised prevalence rate attributable to skin cancer in 2021 eFigure 2. Global maps of age-standardised deaths rate attributable to skin cancer in 2021 eFigure 3. Global maps of age-standardised disability-adjusted life-years (DALYs) rate attributable to skin cancer in 2021 eFigure 4. Decomposition analysis of changes in incidence and DALYs attributable to skin cancer, 1990-2021 eFigure 5. Inequality analysis of DALYs attributable to skin cancer, 1990-2021 eFigure 6. Frontier analysis based on DALYs attributable to skin cancer and SDI, 1990-2021 eFigure 7. Projected burden of cutaneous melanoma by 2050 eFigure 8. Projected burden of squamous cell [file jamadermatol-e251276-s001.pdf]

Supplemental Online Content

Wang R, Chen Y, Shao X, et al. Burden of skin cancer in older adults from 1990 to 2021 and modelled projection to 2050. *JAMA Dermatol*. Published online May 21, 2025. doi:10.1001/jamadermatol.2025.1276

eMethods.
eTable 1. Global burden of cutaneous melanoma among adults aged ⩾65 years in 2021, categorized by sex, region, and SDI
eTable 2. Global burden of squamous cell carcinoma among adults aged ⩾65 years in 2021, categorized by sex, region, and SDI
eTable 3. Global burden of basal cell carcinoma among adults aged ⩾65 years in 2021, categorized by sex, region, and SDI
eTable 4. Burden of cutaneous melanoma in 204 countries and territories in 2021 and the average annual percent change (AAPC) from 1990 to 2021
eTable 5. Burden of squamous cell carcinoma in 204 countries and territories in 2021 and the average annual percent change (AAPC) from 1990 to 2021
eTable 6. Burden of basal cell carcinoma in 204 countries and territories in 2021 and the average annual percent change (AAPC) from 1990 to 2021
eTable 7. Decomposition of change in incidence globally and by SDI quintile, 1990 to 2021
eTable 8. Decomposition of change in DALYs globally and by SDI quintile, 1990 to 2021
eTable 9. Frontier analysis based on SDI and DALYs of cutaneous melanoma in 204 countries and territories
eTable 10. Frontier analysis based on SDI and DALYs of squamous cell carcinoma in 204 countries and territories
eTable 11. Frontier analysis based on SDI and DALYs of basal cell carcinoma in 204 countries and territories
eFigure 1. Global maps of age-standardised prevalence rate attributable to skin cancer in 2021
eFigure 2. Global maps of age-standardised deaths rate attributable to skin cancer in 2021
eFigure 3. Global maps of age-standardised disability-adjusted life-years (DALYs) rate attributable to skin cancer in 2021
eFigure 4. Decomposition analysis of changes in incidence and DALYs attributable to skin cancer, 1990-2021
eFigure 5. Inequality analysis of DALYs attributable to skin cancer, 1990-2021
eFigure 6. Frontier analysis based on DALYs attributable to skin cancer and SDI, 1990-2021

© 2025 Wang R, et al. *JAMA Dermatol*.

eFigure 7. Projected burden of cutaneous melanoma by 2050
eFigure 8. Projected burden of squamous cell carcinoma by 2050
eFigure 9. Projected burden of basal cell carcinoma by 2050

This supplemental material has been provided by the authors to give readers additional information about their work.

© 2025 Wang R, et al. *JAMA Dermatol*.

eMethods

1. Joinpoint Regression Analysis

Temporal trends in the burden of skin cancer were analysed by joinpoint regression, and then the simplest possible model was fitted to the data by joining several different line segments on a logarithmic scale.[1,2] This model employs segmented regression on a log-linear regression model, represented as *In(y)*= β* *x*+constant, to identify inflection points in the trend.[3,4] The grid search method (GSM) was utilized to calculate all possible joinpoints, selecting the one with the smallest mean squared error (MSE) as the optimal inflection point.[5] Building on this, the optimal number of joinpoints was determined using the Monte Carlo permutation test, allowing for a maximum of 5 joinpoints and a minimum of 0. The permutation test starts with the number of connection points k = 0 and k_{max} = 5. If k ≠ k_{max}, then k is incremented by 1 (k = k+1) and the test continues until the model corresponding to k = k_{max}, which is identified as the optimal model. Finally, the annual percentage change (APC), average annual percentage change (AAPC), and the corresponding 95% confidence intervals (CI) at global, regional and national levels are calculated using the geometrically weighted average of the various annual percentage change values in the regression analysis and used to quantify trend changes in incidence, prevalence, deaths and disability-adjusted life-years (DALYs) from 1990 to 2021.[6]

The calculation formula for APC is:

$$APC = (e^{\beta} - 1) \times 100\%$$

where β is the regression coefficient from the log-linear model *In(y)*= β* *x*+constant. AAPC reflects the overall trend change by weighting each segment's APC over a

© 2025 Wang R, et al. *JAMA Dermatol.*

predetermined fixed time interval.[7] This analytical method not only enhances the precision in identifying temporal trends but also improves the robustness of the model.

2. Decomposition Analysis

Decomposition analyses can provide quantitative insights into the specific causes that influence the changes of metric numbers over the defined time periods at the global, regional and national levels.[8] The present study further explored the impact of underlying factors on the epidemiology of incidence and DALYs through decomposition analyse developed by Das Gupta.[9-11] Changes in incidence and DALYs attributable to skin cancer were decomposed into population aging, population growth and epidemiological changes (referred to "age- and population-standardised rates").

Incidence and DALYs is calculated as:

$$A_{a,g,e,t} = \sum_{k=1}^{7} a_{k,t} * p_t * e_{k,t}$$

*A indicates incidence or DALYs

Where $A_{a,g,e,t}$ represents incidence/DALYs number accumulated by population aging, population growth and epidemiological changes in year t, $a_{k,t}$ is the proportion of population for the age group k at year t, p_t is the population size at year t, and is $e_{k,t}$ represented by incidence/DALYs rate for a specific age group k at year t.

Unlike traditional methods such as linear regression, which primarily focus on establishing relationships between variables, decomposition analysis enables a detailed assessment of the independent contributions of each factor to the overall changes in disease burden. We determined the effect of one factor on incidence/DALYs change on the basis that

other factors remained unchanged. Therefore, the formula goes as follows if we calculated the contribution of population aging to incidence/DALYs change:

$$Effect_{2021} = \left[\frac{A_{a_{2021},p_{2021},e_{2021}} + A_{a_{2021},p_{1990},e_{1990}}}{3} + \frac{A_{a_{2021},p_{1990},e_{2021}} + A_{a_{2021},p_{2021},e_{1990}}}{6}\right] - \left[\frac{A_{a_{1990},p_{2021},e_{2021}} + A_{a_{1990},p_{1990},e_{1990}}}{3} + \frac{A_{a_{1990},p_{1990},e_{2021}} + A_{a_{1990},p_{2021},e_{1990}}}{6}\right]$$

*A indicates incidence or DALYs

3. Cross-country Inequality Analysis

This study employed the slope index of inequality (SII) and concentration index (CI), as defined by the World Health Organization (WHO), to measure absolute and relative inequality of skin cancer burden across countries.[12,13] The slope index of inequality is calculated by regressing the DALYs rate on the sociodemographic index (SDI), using the midpoint of the cumulative population distribution sorted by SDI.[14,15] To analyze changes in health inequality, we compared data from 204 countries and territories between 1990 and 2021. To better control for bias and heterogeneity, we employed a robust weighted regression model (rlm) instead of the ordinary linear regression model (lm) in our health inequality analysis. The robust regression model reduces sensitivity to outliers, minimizing bias caused by data heterogeneity or extreme values and leading to a more accurate representation of health inequality. Additionally, the concentration index is calculated by numerically integrating the area under the Lorenz curve, which plots the cumulative proportion of DALYs against the cumulative proportion of the population.[16,17] It ranges from -1 to 1, where: 0 indicates perfect equality (DALYs are evenly distributed across the population); positive values indicate that DALYs are concentrated among higher-SDI groups; negative values

indicate that DALYs are concentrated among lower-SDI groups.

4. Frontier Analysis

Frontier analysis focuses on determining the theoretically lowest age-standardised DALYs rate each country or territory could achieve based on its current development level, serving as a benchmark for optimal performance. This method quantifies the gap between a country's or territory's current burden and its potential minimum burden, thereby identifying areas where improvements can be made. We carried out an improved data envelopment analysis in frontier analysis to estimate the production boundary under the convexity assumption.[9,10,18] Mean age-standardised DALYs for the given SDI were calculated from 1000 bootstrapped sampling to ensure the robustness of the analysis. A smooth frontier line was then fitted through locally weighted regression combined with local polynomial regression, using different smoothing spans (0.3, 0.4, 0.5). In the process of boundary generation, outliers were eliminated by excluding super-efficient elements.

The distance between the observed age-standardised DALYs and the frontier values, named "effective difference", was to determine the gap of age-standardised DALYs that could be attained at a given level of development.[10] For example, when the observed rate of a country was far below the frontier value given its SDI, it implied there might be a great deal of unrecognized opportunities for the country for improvement in terms of age-standardised DALYs. Besides, the change of effective difference for a country over time provided a pattern of the changing burden with SDI.

Through frontier analysis, the countries and territories exhibiting the most pronounced

© 2025 Wang R, et al. *JAMA Dermatol.*

disparities have been identified, including (1) the 15 countries with the largest gap to frontier cancer DALYs among all countries; (2) the 5 countries with the smallest gap to frontier values among relatively low SDI (SDI<0.5) regions; (3) the 5 countries with the largest gap to frontier cancer DALYs among relatively high SDI (SDI>0.85) regions.[19]

5. The Integrated Nested Laplace Approximations (INLA) Framework and the Bayesian age-period-cohort (BAPC) Model Projection

We employed the BAPC model to predict future burdens due to skin cancer by 2050. BAPC model for its capacity to manage complex, high-dimensional, and sparse data frequently encountered in large-scale epidemiological studies like GBD 2021.[20] It builds on the traditional generalized linear model framework within a Bayesian context, allowing the dynamic integration of age, period, and cohort effects.[21] These effects are assumed to evolve continuously over time and are smoothed using a second-order random walk, resulting in more accurate posterior probability predictions. The INLA framework was utilized alongside the BAPC model to approximate the marginal posterior distributions, effectively avoiding the mixing and convergence issues associated with conventional Bayesian methods that rely on Markov chain Monte Carlo sampling techniques.[22] The model's flexibility and robustness in handling time series data make it particularly suitable for long-term disease burden predictions.

Therefore, we used the BAPC model implemented within the INLA framework, which was selected for its computational efficiency and lower error rates compared with traditional methods. This approach enables nuanced predictions of future disease burdens while

© 2025 Wang R, et al. *JAMA Dermatol.*

considering the intricate interactions of age, period, and cohort effects.

References

1. Global burden of 369 diseases and injuries in 204 countries and territories, 1990-2019: a systematic analysis for the Global Burden of Disease Study 2019. *Lancet*. 2020;396(10258):1204-1222. doi:10.1016/s0140-6736(20)30925-9
2. Qiu H, Cao S, Xu R. Cancer incidence, mortality, and burden in China: a time-trend analysis and comparison with the United States and United Kingdom based on the global epidemiological data released in 2020. *Cancer Commun (Lond)*. 2021;41(10):1037-1048. doi:10.1002/cac2.12197
3. Kim HJ, Fay MP, Feuer EJ, Midthune DN. Permutation tests for joinpoint regression with applications to cancer rates. *Stat Med*. 2000;19(3):335-51. doi:10.1002/(sici)1097-0258(20000215)19:3<335::aid-sim336>3.0.co;2-z
4. Bai Z, Han J, An J, et al. The global, regional, and national patterns of change in the burden of congenital birth defects, 1990-2021: an analysis of the global burden of disease study 2021 and forecast to 2040. *EClinicalMedicine*. 2024;77:102873. doi:10.1016/j.eclinm.2024.102873
5. Paik JM, Kabbara K, Eberly KE, Younossi Y, Henry L, Younossi ZM. Global burden of NAFLD and chronic liver disease among adolescents and young adults. *Hepatology*. 2022;75(5):1204-1217. doi:10.1002/hep.32228
6. Zhang J, Ma B, Han X, Ding S, Li Y. Global, regional, and national burdens of HIV and other sexually transmitted infections in adolescents and young adults aged 10-24 years from 1990 to 2019: a trend analysis based on the Global Burden of Disease Study 2019. *Lancet Child Adolesc Health*. 2022;6(11):763-776. doi:10.1016/s2352-4642(22)00219-x
7. Liang X, Lyu Y, Li J, Li Y, Chi C. Global, regional, and national burden of preterm birth, 1990-2021: a systematic analysis from the global burden of disease study 2021. *EClinicalMedicine*. 2024;76:102840. doi:10.1016/j.eclinm.2024.102840
8. Global burden of 288 causes of death and life expectancy decomposition in 204 countries and territories and 811 subnational locations, 1990-2021: a systematic analysis for the Global Burden of Disease Study 2021. *Lancet*. 2024;403(10440):2100-2132. doi:10.1016/s0140-6736(24)00367-2
9. Li H, Lu W, Wang A, Jiang H, Lyu J. Changing epidemiology of chronic kidney disease as a result of type 2 diabetes mellitus from 1990 to 2017: Estimates from Global Burden of Disease 2017. *J Diabetes Investig*. 2021;12(3):346-356. doi:10.1111/jdi.13355
10. Xie Y, Bowe B, Mokdad AH, et al. Analysis of the Global Burden of Disease study highlights the global, regional, and national trends of chronic kidney disease epidemiology from 1990 to 2016. *Kidney Int*. 2018;94(3):567-581. doi:10.1016/j.kint.2018.04.011
11. Chevan A, Sutherland M. Revisiting Das Gupta: refinement and extension of standardization and decomposition. *Demography*. 2009;46(3):429-49. doi:10.1353/dem.0.0060
12. Wagstaff A, Paci P, van Doorslaer E. On the measurement of inequalities in health. *Soc Sci Med*. 1991;33(5):545-57. doi:10.1016/0277-9536(91)90212-u

© 2025 Wang R, et al. *JAMA Dermatol.*

13. Ordunez P, Martinez R, Soliz P, Giraldo G, Mujica OJ, Nordet P. Rheumatic heart disease burden, trends, and inequalities in the Americas, 1990-2017: a population-based study. *Lancet Glob Health*. 2019;7(10):e1388-e1397. doi:10.1016/s2214-109x(19)30360-2
14. Cao F, He YS, Wang Y, et al. Global burden and cross-country inequalities in autoimmune diseases from 1990 to 2019. *Autoimmun Rev*. 2023;22(6):103326. doi:10.1016/j.autrev.2023.103326
15. Chen J, Cui Y, Deng Y, et al. Global, regional, and national burden of cancers attributable to particulate matter pollution from 1990 to 2019 and projection to 2050: Worsening or improving? *J Hazard Mater*. 2024;477:135319. doi:10.1016/j.jhazmat.2024.135319
16. Erreygers G, Clarke P, Van Ourti T. "Mirror, mirror, on the wall, who in this land is fairest of all?"--Distributional sensitivity in the measurement of socioeconomic inequality of health. *J Health Econ*. 2012;31(1):257-70. doi:10.1016/j.jhealeco.2011.10.009
17. Chen Z, Roy K. Calculating concentration index with repetitive values of indicators of economic welfare. *J Health Econ*. 2009;28(1):169-75. doi:10.1016/j.jhealeco.2008.09.004
18. Healthcare Access and Quality Index based on mortality from causes amenable to personal health care in 195 countries and territories, 1990-2015: a novel analysis from the Global Burden of Disease Study 2015. *Lancet*. 2017;390(10091):231-266. doi:10.1016/s0140-6736(17)30818-8
19. Wang F, Ma B, Ma Q, Liu X. Global, regional, and national burden of inguinal, femoral, and abdominal hernias: a systematic analysis of prevalence, incidence, deaths, and DALYs with projections to 2030. *Int J Surg*. 2024;110(4):1951-1967. doi:10.1097/js9.0000000000001071
20. Knoll M, Furkel J, Debus J, Abdollahi A, Karch A, Stock C. An R package for an integrated evaluation of statistical approaches to cancer incidence projection. *BMC Med Res Methodol*. 2020;20(1):257. doi:10.1186/s12874-020-01133-5
21. Li S, Chen H, Man J, et al. Changing trends in the disease burden of esophageal cancer in China from 1990 to 2017 and its predicted level in 25 years. *Cancer Med*. 2021;10(5):1889-1899. doi:10.1002/cam4.3775
22. Chen J, Li C, Bu CLN, et al. Global burden of non-communicable diseases attributable to kidney dysfunction with projection into 2040. *Chin Med J (Engl)*. 2024;doi:10.1097/cm9.0000000000003143

© 2025 Wang R, et al. *JAMA Dermatol*.

eTable 1. Global burden of cutaneous melanoma among adults aged ≥65 years in 2021, categorized by sex, region, and SDI

Characteristics	Incidence			Prevalence			Deaths			DALYs		
	NO.(95% UI)	ASR (95% UI, per 100000)	AAPC (95% CI)	NO.(95% UI)	ASR (95% UI, per 100000)	AAPC (95% CI)	NO.(95% UI)	ASR (95% UI, per 100000)	AAPC (95% CI)	NO.(95% UI)	ASR (95% UI, per 100000)	AAPC (95% CI)
**Global**	153993 (136976 to 165377)	20.54 (18.18 to 22.1)	1.17 (0.95 to 1.4)	983018 (882211 to 1049870)	129.03 (115.29 to 138.02)	1.89 (1.65 to 2.14)	37773 (32800 to 40948)	5.15 (4.45 to 5.59)	-0.05 (-0.15 to 0.06)	667203 (590433 to 725175)	88.27 (77.89 to 96.03)	-0.05 (-0.19 to 0.09)
**Sex**												
Male	88021 (79633 to 95001)	26.92 (24.14 to 29.14)	1.95 (1.72 to 2.18)	557500 (507529 to 599891)	164.03 (148.28 to 176.94)	2.92 (2.7 to 3.13)	20766 (18418 to 22672)	6.64 (5.85 to 7.26)	0.74 (0.56 to 0.91)	377659 (337403 to 413352)	113.08 (100.59 to 123.81)	0.66 (0.5 to 0.83)
Female	65972 (56514 to 72818)	15.72 (13.46 to 17.35)	1.07 (0.95 to 1.18)	425518 (368928 to 467058)	101.46 (87.89 to 111.38)	1.89 (1.68 to 2.09)	17007 (14025 to 19316)	4.05 (3.34 to 4.6)	0.06 (-0.09 to 0.21)	289544 (244544 to 328908)	68.9 (58.21 to 78.26)	0.04 (-0.09 to 0.18)
**Region**												
East Asia	5417 (2868 to 7169)	2.85 (1.51 to 3.77)	1.83 (1.49 to 2.18)	22160 (11371 to 30134)	10.82 (5.54 to 14.71)	7.13 (6.66 to 7.6)	2992 (1604 to 3924)	1.67 (0.89 to 2.18)	-0.05 (-0.24 to 0.14)	51496 (27614 to 67603)	26.67 (14.3 to 35)	-0.03 (-0.34 to 0.28)
Southeast Asia	611 (424 to 847)	1.28 (0.89 to 1.8)	0.9 (0.79 to 1)	488 (329 to 700)	0.97 (0.66 to 1.39)	1.52 (1.42 to 1.61)	598 (415 to 822)	1.29 (0.9 to 1.79)	0.6 (0.5 to 0.7)	10653 (7394 to 14511)	21.39 (14.84 to 29.31)	0.55 (0.51 to 0.59)
Oceania	8 (5 to 13)	1.85 (1.21 to 3.04)	-0.17 (-0.28 to -0.06)	5 (3 to 8)	1.12 (0.74 to 1.84)	-0.11 (-0.24 to 0.01)	9 (6 to 14)	2.07 (1.35 to 3.43)	-0.23 (-0.34 to -0.12)	159 (105 to 262)	33.92 (22.3 to 56.02)	-0.13 (-0.21 to -0.05)

© 2025 Wang R, et al. *JAMA Dermatol*.

Central Asia	372 (320 to 425)	6.57 (5.65 to 7.49)	0.36 (-0.48 to 1.21)	1046 (873 to 1248)	17.01 (14.17 to 20.3)	1.71 (0.83 to 2.61)	236 (204 to 268)	4.34 (3.75 to 4.93)	-0.33 (-1.24 to 0.59)	4053 (3521 to 4633)	70.29 (61.01 to 80.23)	-0.36 (-1.14 to 0.42)
Central Europe	7671 (6660 to 8665)	34.41 (29.87 to 38.88)	3.1 (2.99 to 3.21)	38992 (33332 to 45129)	174.26 (148.93 to 201.68)	5.33 (5.18 to 5.47)	3034 (2686 to 3330)	13.68 (12.11 to 15.02)	1.38 (1.27 to 1.49)	51325 (45770 to 56394)	230.38 (205.35 to 253.16)	1.32 (1.22 to 1.42)
Eastern Europe	8065 (7269 to 8787)	24.22 (21.82 to 26.38)	3.37 (2.88 to 3.87)	41936 (37815 to 45887)	124.7 (112.49 to 136.45)	5.08 (4.67 to 5.5)	3118 (2804 to 3395)	9.45 (8.49 to 10.29)	2 (1.64 to 2.35)	57695 (52313 to 62773)	172.43 (156.24 to 187.68)	2.06 (1.68 to 2.45)
High-income Asia Pacific	3463 (2599 to 4229)	7.04 (5.34 to 8.56)	2.07 (1.24 to 2.91)	23999 (18214 to 29440)	51.57 (39.39 to 62.98)	3.16 (2.77 to 3.56)	805 (634 to 929)	1.48 (1.19 to 1.7)	-0.01 (-0.22 to 0.21)	12859 (10310 to 14864)	26.26 (21.31 to 30.27)	0.15 (-0.07 to 0.36)
Australasia	8363 (6505 to 10459)	158.1 (123.16 to 197.66)	1.16 (0.48 to 1.85)	60780 (47781 to 76077)	1165.26 (917.05 to 1458.33)	1.9 (1.24 to 2.57)	1516 (1238 to 1797)	27.83 (22.79 to 32.97)	-1.15 (-1.58 to -0.71)	26575 (21805 to 31701)	502.22 (412.98 to 598.92)	-1.14 (-1.55 to -0.73)
Western Europe	52661 (45529 to 58336)	56.64 (49.52 to 62.49)	2.89 (2.63 to 3.16)	367577 (320847 to 406411)	409.57 (360.88 to 451.28)	3.93 (3.62 to 4.24)	11091 (9527 to 12107)	11.2 (9.76 to 12.16)	0.81 (0.59 to 1.03)	189663 (166582 to 207583)	205.41 (182.44 to 224.01)	0.92 (0.73 to 1.1)
Southern Latin America	1062 (897 to 1231)	13.03 (11.01 to 15.1)	2.36 (1.74 to 2.99)	3924 (3215 to 4671)	48.62 (39.85 to 57.86)	5.42 (4.61 to 6.24)	548 (469 to 629)	6.68 (5.72 to 7.67)	0.94 (0.38 to 1.49)	9173 (7937 to 10516)	112.83 (97.68 to 129.33)	0.92 (0.44 to 1.4)
High-income North America	54984 (48885 to 59028)	85.69 (76.24 to 91.95)	1.12 (0.76 to 1.48)	392374 (352464 to 419772)	614.6 (551.96 to 657.48)	1.54 (1.24 to 1.84)	7811 (6865 to 8337)	12.07 (10.63 to 12.87)	0.12 (-0.23 to 0.46)	147137 (131901 to 159176)	229.12 (205.61 to 247.77)	0.05 (-0.17 to 0.26)
Caribbean	267 (222 to 317)	5.58 (4.64 to 6.63)	1.78 (1.44 to 2.12)	958 (753 to 1195)	20.23 (15.9 to 25.23)	4.27 (3.95 to 4.58)	151 (127 to 179)	3.13 (2.62 to 3.7)	0.63 (0.35 to 0.91)	2468 (2071 to 2932)	51.63 (43.34 to 61.34)	0.71 (0.43 to 1)
Andean Latin	435 (312 to	8.77 (6.28 to	1.49 (0.98	948 (645 to	18.78 (12.79 to	5.05 (4.6	299 (219 to	6.08 (4.45	0.2 (-0.17	4920 (3611 to	98.7 (72.45 to	0.22 (-0.17

© 2025 Wang R, et al. *JAMA Dermatol*.

America	570)	11.5)	to 2)	1328)	26.32)	to 5.5)	387)	to 7.87)	to 0.57)	6388)	128.1)	to 0.6)
Central Latin America	1477 (1284 to 1665)	7.09 (6.16 to 7.98)	2.1 (1.9 to 2.29)	3743 (3170 to 4402)	17.46 (14.78 to 20.55)	5.19 (4.66 to 5.73)	967 (849 to 1078)	4.69 (4.11 to 5.22)	1 (0.82 to 1.19)	16117 (14260 to 17957)	76.68 (67.82 to 85.4)	1.02 (0.85 to 1.19)
Tropical Latin America	1970 (1709 to 2172)	9 (7.79 to 9.93)	1.68 (1.49 to 1.87)	4393 (3807 to 5023)	19.49 (16.86 to 22.3)	4.25 (3.61 to 4.89)	1336 (1162 to 1467)	6.17 (5.35 to 6.78)	0.66 (0.46 to 0.86)	22439 (19924 to 24488)	101.68 (90.05 to 111.03)	0.65 (0.46 to 0.83)
North Africa and Middle East	4126 (2089 to 5582)	13.05 (6.55 to 17.7)	3.25 (3.07 to 3.43)	16114 (8155 to 22475)	46.45 (23.35 to 64.97)	7.53 (7.35 to 7.71)	693 (344 to 913)	2.39 (1.17 to 3.17)	-0.12 (-0.29 to 0.05)	12532 (6358 to 16439)	39.51 (19.82 to 52.06)	0.02 (-0.21 to 0.25)
South Asia	1618 (1046 to 2201)	1.38 (0.89 to 1.88)	1.22 (1.06 to 1.38)	2201 (1333 to 2946)	1.76 (1.06 to 2.35)	3.26 (2.94 to 3.58)	1292 (843 to 1767)	1.15 (0.75 to 1.57)	0.55 (0.33 to 0.77)	24130 (15764 to 32928)	20.06 (13.08 to 27.4)	0.47 (0.28 to 0.65)
Central Sub-Saharan Africa	104 (63 to 160)	3.29 (1.94 to 5.11)	0.63 (0.56 to 0.71)	93 (57 to 144)	2.64 (1.57 to 4.12)	1.38 (1.25 to 1.5)	99 (60 to 154)	3.31 (1.93 to 5.22)	0.39 (0.33 to 0.44)	1877 (1155 to 2927)	55.43 (33.29 to 86.74)	0.33 (0.28 to 0.39)
Eastern Sub-Saharan Africa	546 (330 to 730)	4.72 (2.87 to 6.32)	0.25 (0.22 to 0.29)	487 (289 to 661)	3.95 (2.36 to 5.35)	1.01 (0.89 to 1.14)	508 (308 to 680)	4.55 (2.78 to 6.1)	-0.04 (-0.07 to 0)	9755 (5884 to 13094)	80.94 (49.05 to 108.55)	-0.07 (-0.1 to -0.04)
Southern Sub-Saharan Africa	421 (229 to 526)	10.06 (5.37 to 12.6)	1.18 (1.07 to 1.3)	509 (266 to 651)	11.09 (5.74 to 14.22)	2.28 (1.97 to 2.59)	347 (190 to 434)	8.63 (4.63 to 10.83)	0.73 (0.55 to 0.92)	6213 (3487 to 7726)	142.53 (78.68 to 177.83)	0.76 (0.56 to 0.95)
Western Sub-Saharan Africa	352 (162 to 466)	2.73 (1.29 to 3.58)	0.58 (0.5 to 0.66)	292 (130 to 392)	2.12 (0.96 to 2.83)	1.15 (1.07 to 1.24)	325 (155 to 425)	2.62 (1.28 to 3.4)	0.28 (0.22 to 0.35)	5963 (2780 to 7867)	44.26 (21.02 to 58.01)	0.2 (0.15 to 0.25)
**SDI category**												
High SDI	112765 (99683 to 121220)	54.15 (48.19 to 58.07)	1.52 (1.24 to 1.8)	789982 (705536 to 845857)	387.24 (347.53 to 413.9)	2.14 (1.88 to 2.39)	20518 (17959 to 21981)	9.49 (8.38 to 10.13)	0.12 (-0.08 to 0.32)	364292 (325522 to 393120)	175.6 (157.91 to 189.12)	0.13 (-0.06 to 0.32)

© 2025 Wang R, et al. *JAMA Dermatol*.

High-middle SDI	29160 (25207 to 32537)	16.18 (13.95 to 18.07)	2.08 (1.85 to 2.31)	165029 (143142 to 184728)	89.84 (77.78 to 100.7)	3.69 (3.43 to 3.96)	9429 (8184 to 10306)	5.34 (4.62 to 5.84)	0.35 (0.23 to 0.46)	165333 (144361 to 180600)	91.1 (79.39 to 99.61)	0.38 (0.24 to 0.53)
Middle SDI	8649 (6266 to 10183)	4 (2.89 to 4.7)	1.66 (1.41 to 1.92)	23260 (16417 to 28053)	9.89 (6.98 to 11.93)	5.11 (4.92 to 5.29)	5101 (3781 to 5916)	2.47 (1.83 to 2.86)	0.4 (0.3 to 0.51)	87609 (64993 to 101796)	39.6 (29.36 to 45.98)	0.36 (0.21 to 0.51)
Low-middle SDI	2265 (1597 to 2741)	2.07 (1.47 to 2.51)	1.17 (0.98 to 1.36)	2942 (2131 to 3499)	2.5 (1.81 to 2.97)	2.91 (2.65 to 3.18)	1774 (1279 to 2208)	1.69 (1.22 to 2.09)	0.5 (0.36 to 0.64)	32095 (23074 to 40010)	28.45 (20.49 to 35.4)	0.52 (0.43 to 0.61)
Low SDI	980 (595 to 1240)	2.76 (1.67 to 3.49)	0.31 (0.28 to 0.35)	859 (513 to 1096)	2.26 (1.35 to 2.88)	1.04 (0.95 to 1.12)	889 (543 to 1137)	2.61 (1.59 to 3.34)	0 (-0.11 to 0.1)	16831 (10279 to 21575)	45.37 (27.68 to 58.11)	-0.09 (-0.12 to -0.06)

Abbreviations: UI, uncertainty interval; ASR, age-standardised rate per 100,000; AAPC, average annual percent change; CI, confidence interval; DALYs, disability-adjusted life-year; SDI, socio-demographic index.

© 2025 Wang R, et al. *JAMA Dermatol*.

eTable 2. Global burden of squamous cell carcinoma among adults aged ≥65 years in 2021, categorized by sex, region, and SDI

Characteristics	Incidence			Prevalence			Deaths			DALYs		
	NO.(95% UI)	ASR (95% UI, per 100000)	AAPC (95% CI)	NO.(95% UI)	ASR (95% UI, per 100000)	AAPC (95% CI)	NO.(95% UI)	ASR (95% UI, per 100000)	AAPC (95% CI)	NO.(95% UI)	ASR (95% UI, per 100000)	AAPC (95% CI)
**Global**	1463424 (1219157 to 1746516)	196.24 (163.32 to 234.39)	1.46 (1.26 to 1.66)	1763685 (1401932 to 2258696)	236.91 (188.23 to 303.82)	1.81 (1.67 to 1.96)	44024 (36746 to 49070)	6.16 (5.13 to 6.87)	0.27 (0.17 to 0.36)	706405 (604692 to 787103)	95.5 (81.65 to 106.39)	0.31 (0.18 to 0.44)
**Sex**												
Male	925139 (770372 to 1105531)	286.89 (238.37 to 343.3)	0.87 (0.67 to 1.07)	1126200 (895475 to 1434758)	350.42 (278.35 to 447.54)	1.11 (0.81 to 1.41)	24416 (19900 to 27765)	8.32 (6.79 to 9.41)	0.69 (0.53 to 0.84)	415210 (346814 to 473283)	130.22 (108.85 to 147.84)	0.62 (0.5 to 0.74)
Female	538285 (447460 to 643752)	128.29 (106.66 to 153.44)	0.05 (-0.15 to 0.26)	637485 (503868 to 821445)	151.94 (120.09 to 195.83)	0.34 (0.04 to 0.64)	19608 (15785 to 22195)	4.65 (3.74 to 5.26)	0.46 (0.22 to 0.71)	291195 (244439 to 328730)	69.15 (58.09 to 78.05)	0.38 (0.21 to 0.56)
**Region**												
East Asia	82192 (55276 to 119016)	42.87 (28.54 to 62.57)	6.05 (5.72 to 6.37)	178888 (119682 to 251772)	93.87 (62.5 to 133.28)	7.05 (6.76 to 7.34)	12857 (9711 to 15911)	7.45 (5.59 to 9.2)	0.63 (0.44 to 0.82)	213609 (164869 to 263395)	113.53 (87.2 to 139.7)	0.56 (0.36 to 0.76)
Southeast Asia	728 (479 to 1060)	1.55 (1.02 to 2.26)	-0.21 (-0.3 to -0.13)	906 (582 to 1316)	1.92 (1.24 to 2.79)	-0.22 (-0.31 to -0.13)	2232 (1723 to 2784)	5.12 (3.94 to 6.45)	0.47 (0.41 to 0.54)	36674 (28337 to 45201)	76.93 (59.41 to 95.54)	0.41 (0.32 to 0.5)
Oceania	0 (0 to 0)	0.02 (0 to 0.04)	-0.03 (-0.04 to	0 (0 to 1)	0.05 (0.01 to 0.12)	-0.1 (-0.11 to -0.09)	17 (10 to 25)	5.26 (3.39 to 7.81)	0.17 (0.04 to 0.29)	257 (159 to 390)	68.43 (43.3 to 102.58)	0.27 (0.16 to 0.37)

© 2025 Wang R, et al. *JAMA Dermatol.*

			-0.02)									
Central Asia	16 (9 to 24)	0.26 (0.16 to 0.41)	-0.12 (-0.15 to -0.08)	33 (18 to 55)	0.56 (0.31 to 0.92)	-0.14 (-0.2 to -0.09)	526 (459 to 589)	10.22 (8.87 to 11.46)	3.24 (2.67 to 3.82)	7882 (6937 to 8829)	144.61 (126.8 to 162)	3.23 (2.34 to 4.13)
Central Europe	5237 (3633 to 7448)	23.55 (16.32 to 33.53)	0.22 (0.19 to 0.26)	8312 (5671 to 11674)	37.37 (25.47 to 52.54)	0.13 (0.07 to 0.19)	1374 (1201 to 1527)	6.24 (5.45 to 6.94)	-2.77 (-3.38 to -2.16)	18879 (16664 to 20993)	85.34 (75.28 to 94.92)	-2.64 (-3.19 to -2.08)
Eastern Europe	5772 (3891 to 8330)	17.57 (11.75 to 25.46)	0.51 (0.49 to 0.54)	9661 (6335 to 13815)	29.44 (19.28 to 42.19)	0.53 (0.48 to 0.58)	2076 (1841 to 2265)	6.43 (5.69 to 7.02)	0.3 (-0.06 to 0.66)	30664 (27514 to 33382)	93.69 (83.96 to 102.04)	0.28 (-0.06 to 0.62)
High-income Asia Pacific	2711 (1805 to 3977)	5.23 (3.52 to 7.58)	1.26 (1.21 to 1.3)	5317 (3429 to 7726)	10.23 (6.64 to 14.69)	1.21 (1.12 to 1.3)	1726 (1307 to 2071)	2.63 (2.06 to 3.18)	-0.93 (-1.48 to -0.38)	19740 (15608 to 23918)	33.9 (27.75 to 41.57)	-1.04 (-1.66 to -0.41)
Australasia	23036 (15095 to 33101)	433.56 (285.26 to 620.77)	-0.09 (-0.13 to -0.05)	26160 (17268 to 37839)	492.3 (325.57 to 710.34)	-0.11 (-0.17 to -0.06)	874 (724 to 987)	15.37 (12.8 to 17.36)	0.83 (0.2 to 1.45)	12422 (10553 to 14040)	226.92 (193.43 to 256.48)	0.48 (-0.13 to 1.09)
Western Europe	49861 (33420 to 71649)	51.54 (34.63 to 73.87)	0.56 (0.51 to 0.61)	62112 (41927 to 89728)	64.36 (43.43 to 92.55)	0.46 (0.41 to 0.5)	6744 (5603 to 7392)	6.01 (5.06 to 6.56)	0.16 (-0.24 to 0.57)	83506 (71518 to 90910)	80.14 (69.62 to 86.85)	0.02 (-0.39 to 0.44)
Southern Latin America	2101 (1397 to 3068)	25.65 (17.08 to 37.42)	0.12 (0.11 to 0.14)	3648 (2395 to 5320)	44.52 (29.24 to 64.86)	-0.02 (-0.05 to 0)	659 (567 to 732)	7.95 (6.84 to 8.82)	0.75 (0.18 to 1.33)	8983 (7859 to 9936)	109.25 (95.64 to 120.84)	0.65 (0.38 to 0.92)
High-income North America	1280160 (1077652 to 1496614)	1986.41 (1674.51 to 2319.54)	2.65 (2.48 to 2.82)	1453914 (1172531 to 1842741)	2253.79 (1818.76 to 2855.07)	2.94 (2.83 to 3.05)	4591 (3929 to 4961)	6.94 (5.97 to 7.48)	0.71 (0.29 to 1.13)	121312 (99446 to 149407)	187.18 (153.44 to 230.66)	1.39 (1.2 to 1.57)
Caribbean	95 (63 to 136)	2 (1.33 to 2.85)	-2.47 (-2.74 to	113 (74 to 160)	2.38 (1.56 to 3.36)	-2.21 (-2.47 to -1.95)	609 (509 to 708)	12.28 (10.3 to 14.27)	1.15 (0.68 to 1.62)	8186 (6858 to 9530)	168.27 (141.26 to 195.89)	1.11 (0.61 to 1.62)

© 2025 Wang R, et al. *JAMA Dermatol*.

			-2.19)									
Andean Latin America	60 (40 to 86)	1.2 (0.8 to 1.72)	-2.7 (-2.91 to -2.49)	111 (68 to 163)	2.22 (1.36 to 3.26)	-2.17 (-2.33 to -2.02)	499 (371 to 630)	10.27 (7.64 to 12.97)	2.09 (1.28 to 2.91)	6777 (5010 to 8573)	137.92 (102.03 to 174.35)	2.02 (1.22 to 2.83)
Central Latin America	1688 (1167 to 2390)	8.02 (5.54 to 11.37)	-0.25 (-0.27 to -0.22)	2884 (1894 to 4109)	13.73 (9.01 to 19.6)	-0.36 (-0.38 to -0.35)	1800 (1548 to 2024)	8.86 (7.63 to 9.95)	-0.87 (-1.14 to -0.6)	24972 (21693 to 28140)	121.14 (105.32 to 136.34)	-1.01 (-1.28 to -0.74)
Tropical Latin America	4388 (3183 to 5949)	19.92 (14.44 to 27.04)	-0.12 (-0.23 to -0.02)	4778 (3474 to 6373)	21.73 (15.79 to 29.01)	-0.15 (-0.26 to -0.04)	2461 (2089 to 2692)	11.54 (9.78 to 12.62)	1.34 (0.94 to 1.74)	34849 (30550 to 37663)	160.75 (140.6 to 173.85)	1.16 (0.69 to 1.63)
North Africa and Middle East	1774 (1194 to 2529)	5.67 (3.79 to 8.12)	-0.15 (-0.26 to -0.03)	2397 (1597 to 3419)	7.62 (5.07 to 10.94)	-0.14 (-0.25 to -0.02)	694 (507 to 959)	2.62 (1.91 to 3.65)	0.41 (-0.01 to 0.84)	10052 (7423 to 13765)	34.54 (25.38 to 47.59)	0.46 (0.08 to 0.83)
South Asia	1097 (674 to 1620)	0.97 (0.59 to 1.44)	0.44 (0.39 to 0.49)	1377 (803 to 2121)	1.22 (0.71 to 1.88)	0.41 (0.36 to 0.46)	3436 (2722 to 4562)	3.6 (2.82 to 4.76)	0.72 (0.11 to 1.33)	53907 (42899 to 71887)	50.8 (40.17 to 67.51)	0.65 (0.15 to 1.16)
Central Sub-Saharan Africa	8 (4 to 13)	0.25 (0.14 to 0.4)	-0.17 (-0.18 to -0.15)	14 (7 to 24)	0.43 (0.22 to 0.72)	-0.42 (-0.42 to -0.41)	103 (31 to 180)	4.13 (1.27 to 7.41)	1.2 (1.13 to 1.27)	1727 (522 to 2996)	59.07 (18.05 to 104.35)	1.17 (1.12 to 1.23)
Eastern Sub-Saharan Africa	62 (39 to 92)	0.56 (0.35 to 0.83)	-0.04 (-0.07 to -0.01)	87 (51 to 136)	0.77 (0.46 to 1.21)	-0.19 (-0.22 to -0.15)	353 (100 to 572)	3.96 (1.14 to 6.48)	1.17 (1.1 to 1.23)	5685 (1595 to 9130)	55.96 (15.9 to 90.72)	1.08 (1.03 to 1.13)
Southern Sub-Saharan Africa	2422 (1635 to 3430)	56.74 (38.01 to 80.88)	1.45 (1.3 to 1.6)	2951 (2019 to 4270)	69.32 (47.14 to 100.85)	1.27 (1.12 to 1.42)	262 (195 to 308)	7.36 (5.49 to 8.63)	1.31 (1.06 to 1.56)	4291 (3205 to 5034)	107.83 (80.91 to 126.28)	1.39 (1.12 to 1.65)
Western Sub-Saharan Africa	15 (9 to 23)	0.11 (0.06 to 0.17)	0.31 (0.23 to 0.39)	22 (12 to 36)	0.16 (0.09 to 0.26)	0.19 (0.12 to 0.26)	132 (47 to 186)	1.18 (0.44 to 1.63)	1.72 (1.6 to 1.84)	2030 (689 to 2874)	16.84 (5.95 to 23.62)	1.78 (1.67 to 1.9)

© 2025 Wang R, et al. *JAMA Dermatol*.

**SDI category**												
High SDI	1346008 (1129226 to 1584964)	642.63 (539.97 to 755.81)	2.22 (2.12 to 2.33)	1540880 (1233984 to 1959735)	733.09 (587.48 to 930.67)	2.43 (2.32 to 2.54)	12392 (10386 to 13592)	5.32 (4.52 to 5.82)	-0.12 (-0.35 to 0.11)	220433 (187537 to 253777)	101.43 (86.67 to 116.95)	0.31 (0.17 to 0.45)
High-middle SDI	66555 (45899 to 94307)	37.19 (25.55 to 52.84)	1.9 (1.85 to 1.96)	121295 (83077 to 168339)	67.7 (46.28 to 94.2)	2.45 (2.12 to 2.78)	12058 (10209 to 13956)	7.08 (5.97 to 8.18)	-0.01 (-0.23 to 0.22)	178007 (153097 to 207407)	101.06 (86.67 to 117.43)	0.04 (-0.18 to 0.26)
Middle SDI	48255 (31996 to 70197)	21.9 (14.44 to 32.03)	3.45 (3.16 to 3.74)	97857 (65039 to 138518)	44.67 (29.59 to 63.64)	4.74 (4.28 to 5.21)	14259 (11178 to 16490)	7.28 (5.72 to 8.4)	0.49 (0.3 to 0.69)	225463 (176221 to 260853)	106.22 (83.29 to 122.71)	0.42 (0.26 to 0.58)
Low-middle SDI	2221 (1464 to 3202)	2.04 (1.34 to 2.95)	-0.17 (-0.24 to -0.09)	3120 (2012 to 4503)	2.87 (1.85 to 4.16)	-0.12 (-0.17 to -0.07)	4392 (3594 to 5290)	4.77 (3.87 to 5.74)	1.34 (0.87 to 1.8)	67692 (55601 to 81591)	66.54 (54.39 to 80.14)	1.23 (1.11 to 1.35)
Low SDI	239 (151 to 349)	0.69 (0.44 to 1.01)	0.39 (0.36 to 0.42)	327 (195 to 496)	0.95 (0.57 to 1.44)	0.2 (0.16 to 0.24)	872 (468 to 1210)	3.1 (1.65 to 4.37)	1.23 (0.81 to 1.66)	14106 (7593 to 19431)	44.14 (23.61 to 61.45)	1.15 (0.8 to 1.49)

Abbreviations: UI, uncertainty interval; ASR, age-standardised rate per 100,000; AAPC, average annual percent change; CI, confidence interval; DALYs, disability-adjusted life-year; SDI, socio-demographic index.

© 2025 Wang R, et al. *JAMA Dermatol*.

eTable 3. Global burden of basal cell carcinoma among adults aged ≥65 years in 2021, categorized by sex, region, and SDI

Characteristics	Incidence			Prevalence			DALYs		
	NO.(95% UI)	ASR (95% UI, per 100000)	AAPC (95% CI)	NO.(95% UI)	ASR (95% UI, per 100000)	AAPC (95% CI)	NO.(95% UI)	ASR (95% UI, per 100000)	AAPC (95% CI)
**Global**	2802354 (2344258 to 3305009)	371.97 (310.75 to 439.58)	1.46 (1.27 to 1.64)	300169 (242015 to 369305)	39.89 (32.08 to 49.15)	1.19 (0.98 to 1.4)	1208 (547 to 2334)	0.16 (0.07 to 0.31)	1.17 (0.95 to 1.39)
**Sex**									
Male	1664625 (1401821 to 1953802)	504.43 (423.5 to 594.71)	1.3 (1.05 to 1.55)	173517 (140856 to 212300)	52.73 (42.59 to 64.72)	1.13 (0.85 to 1.4)	694 (315 to 1331)	0.21 (0.1 to 0.4)	1.09 (0.8 to 1.37)
Female	1137729 (943444 to 1354623)	271.14 (224.83 to 322.79)	0.37 (0.16 to 0.59)	126652 (100600 to 157287)	30.19 (23.97 to 37.49)	0.16 (0.03 to 0.28)	514 (232 to 1012)	0.12 (0.06 to 0.24)	0.14 (-0.02 to 0.29)
**Region**									
East Asia	349449 (273043 to 439115)	175.36 (136.31 to 221.85)	6.85 (6.44 to 7.26)	38904 (29837 to 49657)	19.52 (14.89 to 25.04)	6.32 (5.92 to 6.71)	167 (72 to 320)	0.08 (0.04 to 0.16)	6.23 (5.83 to 6.63)
Southeast Asia	4437 (2976 to 6264)	9.22 (6.14 to 13.09)	-1.35 (-1.42 to -1.28)	637 (413 to 927)	1.32 (0.85 to 1.93)	-1.26 (-1.35 to -1.17)	3 (1 to 5)	0.01 (0 to 0.01)	-1.27 (-1.36 to -1.18)
Oceania	1 (0 to 2)	0.17 (0.04 to 0.37)	-0.02 (-0.02 to -0.01)	0 (0 to 0)	0.02 (0.01 to 0.06)	-0.03 (-0.04 to -0.03)	0 (0 to 0)	0 (0 to 0)	-0.03 (-0.05 to -0.02)
Central Asia	8630 (6255 to 11517)	149.6 (107.65 to 201.29)	0.04 (0.03 to 0.04)	1077 (756 to 1449)	18.78 (13.07 to 25.37)	0 (-0.01 to 0.01)	5 (2 to 9)	0.08 (0.03 to 0.16)	-0.01 (-0.03 to 0.01)
Central Europe	39224 (28704 to 52833)	176.19 (128.77 to 237.61)	0.45 (0.39 to 0.51)	5116 (3578 to 6888)	22.98 (16.05 to 30.97)	0.38 (0.29 to 0.47)	21 (9 to 43)	0.1 (0.04 to 0.19)	0.36 (0.2 to 0.52)
Eastern Europe	40211 (29004 to 54590)	121.98 (87.56 to 166.42)	0.55 (0.53 to 0.57)	5337 (3746 to 7219)	16.22 (11.34 to 21.99)	0.47 (0.45 to 0.49)	23 (10 to 46)	0.07 (0.03 to 0.14)	0.46 (0.43 to 0.49)

© 2025 Wang R, et al. *JAMA Dermatol*.

High-income Asia Pacific	14104 (10381 to 18952)	27.45 (20.52 to 36.65)	1.29 (1.23 to 1.34)	2827 (1937 to 3887)	5.57 (3.86 to 7.6)	1.05 (1.01 to 1.1)	12 (5 to 25)	0.02 (0.01 to 0.05)	1.05 (1.01 to 1.1)
Australasia	8661 (6109 to 11895)	163.41 (115.67 to 223.61)	-0.04 (-0.07 to -0.01)	1306 (882 to 1790)	24.66 (16.71 to 33.7)	-0.07 (-0.09 to -0.04)	5 (2 to 12)	0.1 (0.03 to 0.23)	-0.02 (-0.25 to 0.2)
Western Europe	223341 (158109 to 309588)	234.45 (166.52 to 323.9)	0.02 (-0.05 to 0.1)	31012 (21160 to 42640)	32.67 (22.42 to 44.83)	-0.04 (-0.13 to 0.05)	125 (52 to 247)	0.13 (0.05 to 0.26)	-0.04 (-0.15 to 0.07)
Southern Latin America	11674 (8325 to 16033)	142.86 (101.99 to 195.98)	-0.01 (-0.04 to 0.01)	1689 (1153 to 2317)	20.65 (14.12 to 28.32)	-0.04 (-0.07 to -0.02)	7 (3 to 15)	0.09 (0.03 to 0.18)	-0.02 (-0.12 to 0.09)
High-income North America	1987127 (1708265 to 2278244)	3092.24 (2660.92 to 3541.03)	2.81 (2.73 to 2.89)	198415 (165036 to 239194)	308.65 (256.91 to 371.88)	2.63 (2.55 to 2.72)	779 (361 to 1518)	1.21 (0.56 to 2.36)	2.59 (2.5 to 2.68)
Caribbean	1494 (1043 to 2082)	31.23 (21.88 to 43.44)	-0.47 (-0.59 to -0.35)	220 (147 to 308)	4.6 (3.09 to 6.43)	-0.45 (-0.52 to -0.37)	1 (0 to 2)	0.02 (0.01 to 0.04)	-0.45 (-0.52 to -0.38)
Andean Latin America	3812 (2774 to 5089)	76.35 (55.55 to 101.95)	-1.15 (-1.22 to -1.08)	535 (377 to 728)	10.71 (7.54 to 14.59)	-1.02 (-1.09 to -0.95)	2 (1 to 5)	0.05 (0.02 to 0.1)	-1.02 (-1.12 to -0.91)
Central Latin America	37626 (26803 to 51420)	180.06 (128.39 to 246.12)	-0.05 (-0.05 to -0.05)	4566 (3196 to 6203)	21.85 (15.28 to 29.66)	-0.07 (-0.07 to -0.06)	19 (8 to 38)	0.09 (0.04 to 0.18)	-0.07 (-0.08 to -0.05)
Tropical Latin America	42649 (33269 to 53198)	192.51 (150.08 to 240.29)	-2.25 (-2.39 to -2.12)	4591 (3518 to 5880)	20.75 (15.88 to 26.58)	-2.13 (-2.24 to -2.02)	19 (8 to 39)	0.09 (0.04 to 0.17)	-1.99 (-2.13 to -1.85)
North Africa and Middle East	12429 (8958 to 16836)	38.48 (27.61 to 52.52)	-0.56 (-0.75 to -0.36)	1790 (1244 to 2455)	5.55 (3.83 to 7.65)	-0.46 (-0.63 to -0.3)	8 (3 to 15)	0.02 (0.01 to 0.05)	-0.47 (-0.63 to -0.32)
South Asia	7669 (5186 to 10835)	6.59 (4.42 to 9.37)	0.44 (0.33 to 0.55)	945 (609 to 1377)	0.81 (0.52 to 1.19)	0.41 (0.29 to 0.53)	4 (2 to 8)	0 (0 to 0.01)	0.41 (0.29 to 0.53)
Central Sub-Saharan Africa	845 (594 to 1165)	25.86 (18.01 to 36.02)	-0.11 (-0.12 to -0.11)	103 (70 to 143)	3.19 (2.13 to 4.46)	-0.14 (-0.15 to -0.14)	0 (0 to 1)	0.01 (0.01 to 0.03)	-0.15 (-0.15 to -0.14)
Eastern Sub-Saharan Africa	2325 (1622 to 3191)	20.61 (14.27 to 28.54)	-0.11 (-0.12 to -0.1)	296 (200 to 416)	2.65 (1.77 to 3.74)	-0.13 (-0.13 to -0.12)	1 (1 to 3)	0.01 (0 to 0.02)	-0.13 (-0.14 to -0.13)

© 2025 Wang R, et al. *JAMA Dermatol*.

Southern Sub-Saharan Africa	4708 (3345 to 6283)	108.33 (76.56 to 145.62)	0.56 (0.36 to 0.76)	545 (382 to 738)	12.6 (8.75 to 17.15)	0.5 (0.34 to 0.66)	2 (1 to 5)	0.06 (0.02 to 0.11)	0.51 (0.26 to 0.76)
Western Sub-Saharan Africa	1939 (1321 to 2706)	14.81 (10.03 to 20.8)	0.05 (0.04 to 0.06)	258 (170 to 370)	1.98 (1.29 to 2.85)	0.01 (0 to 0.02)	1 (0 to 2)	0.01 (0 to 0.02)	0.01 (0 to 0.02)
**SDI category**									
High SDI	2195956 (1871916 to 2543442)	1060.32 (905.22 to 1224.68)	2.2 (2.07 to 2.33)	227890 (186825 to 276787)	109.82 (90.29 to 133.11)	1.93 (1.77 to 2.08)	901 (411 to 1751)	0.44 (0.2 to 0.85)	1.89 (1.72 to 2.05)
High-middle SDI	305763 (231437 to 396535)	169.41 (127.76 to 220.62)	1.15 (1.06 to 1.23)	38182 (28027 to 50054)	21.18 (15.47 to 27.83)	0.8 (0.73 to 0.88)	161 (69 to 314)	0.09 (0.04 to 0.17)	0.82 (0.74 to 0.91)
Middle SDI	266740 (204959 to 339400)	118.58 (90.71 to 151.81)	1.71 (1.43 to 1.99)	29967 (22579 to 38666)	13.35 (10 to 17.3)	1.62 (1.35 to 1.9)	128 (56 to 244)	0.06 (0.02 to 0.11)	1.69 (1.42 to 1.97)
Low-middle SDI	27920 (19965 to 37862)	25.71 (18.3 to 35.08)	-0.83 (-0.95 to -0.72)	3373 (2364 to 4664)	3.12 (2.17 to 4.33)	-0.75 (-0.85 to -0.64)	15 (6 to 29)	0.01 (0.01 to 0.03)	-0.64 (-0.74 to -0.55)
Low SDI	5104 (3549 to 7089)	14.47 (9.99 to 20.25)	-0.11 (-0.13 to -0.09)	642 (432 to 910)	1.83 (1.22 to 2.61)	-0.14 (-0.16 to -0.12)	3 (1 to 6)	0.01 (0 to 0.02)	-0.14 (-0.16 to -0.12)

Abbreviations: UI, uncertainty interval; ASR, age-standardised rate per 100,000; AAPC, average annual percent change; CI, confidence interval; DALYs, disability-adjusted life-year; SDI, socio-demographic index.

© 2025 Wang R, et al. *JAMA Dermatol*.

eTable 4. Burden of cutaneous melanoma in 204 countries and territories in 2021 and the average annual percent change (AAPC) from 1990 to 2021

Country	Incidence		Prevalence		Deaths		DALYs	
	ASR (95% UI)	AAPC (95% CI)	ASR (95% UI)	AAPC (95% CI)	ASR (95% UI)	AAPC (95% CI)	ASR (95% UI)	AAPC (95% CI)
Afghanistan	4.35 (1.42 to 9.03)	1.18 (1.15 to 1.21)	3.67 (1.19 to 7.97)	2.04 (1.94 to 2.15)	2.73 (0.87 to 5.51)	0.17 (0.13 to 0.21)	44.27 (14.46 to 91.99)	0.16 (0.11 to 0.2)
Albania	11.04 (6.24 to 17.03)	1.73 (1.53 to 1.93)	50.32 (27.86 to 78.82)	5.51 (5.1 to 5.93)	5.27 (3.07 to 7.93)	-0.1 (-0.34 to 0.15)	84.24 (48.68 to 128.31)	-0.19 (-0.44 to 0.05)
Algeria	3.1 (1.55 to 5.11)	2.82 (2.55 to 3.09)	8.37 (3.79 to 14.48)	6.49 (6.21 to 6.76)	0.72 (0.39 to 1.14)	0.14 (-0.03 to 0.32)	11.42 (5.97 to 18.33)	0.28 (0.17 to 0.39)
American Samoa	3.31 (1.88 to 5.01)	0.6 (0.16 to 1.05)	2.09 (1.2 to 3.19)	0.66 (0.21 to 1.11)	3.65 (2.05 to 5.62)	0.43 (-0.05 to 0.93)	55.97 (32.12 to 85.24)	0.62 (0.22 to 1.01)
Andorra	38.79 (21.24 to 62.47)	0.46 (-0.09 to 1.01)	268.93 (147.2 to 439.2)	1.23 (0.84 to 1.63)	7.31 (4.14 to 11.44)	-1.42 (-1.81 to -1.02)	134.87 (75.88 to 213.26)	-1.27 (-1.67 to -0.88)
Angola	3.43 (1.96 to 5.44)	0.95 (0.86 to 1.04)	2.8 (1.61 to 4.47)	1.84 (1.7 to 1.99)	3.43 (1.92 to 5.5)	0.66 (0.57 to 0.76)	57.34 (32.66 to 91.44)	0.57 (0.48 to 0.66)
Antigua and Barbuda	10.69 (9 to 12.65)	2.23 (1.55 to	28.45 (21.32 to 37.36)	4.18 (3.48 to 4.88)	6.79 (5.95 to 7.75)	1.52 (0.77 to 2.27)	110.21 (97.11 to 126.14)	1.37 (0.59 to 2.16)

© 2025 Wang R, et al. *JAMA Dermatol*.

		2.91)						
Argentina	11.47 (9.14 to 14.14)	2.28 (1.69 to 2.88)	33.44 (24.43 to 44.69)	4.74 (3.85 to 5.64)	6.67 (5.48 to 8.05)	1.19 (0.69 to 1.69)	112.42 (92.75 to 135.07)	1.14 (0.61 to 1.67)
Armenia	5.37 (4.26 to 6.57)	2.73 (1.82 to 3.64)	18.21 (13.36 to 23.62)	4.72 (4.16 to 5.28)	2.99 (2.46 to 3.51)	1.55 (0.74 to 2.37)	47.45 (39.17 to 55.79)	1.45 (0.59 to 2.32)
Australia	150.13 (113.35 to 194.06)	0.91 (0.18 to 1.65)	1105.19 (839.36 to 1434.98)	1.92 (1.41 to 2.42)	27.35 (21.96 to 32.99)	-1.45 (-2.1 to -0.8)	491.63 (396.84 to 596.61)	-1.37 (-1.87 to -0.86)
Austria	51.94 (39.39 to 66.01)	2.34 (1.65 to 3.04)	353.24 (267.61 to 452.05)	3.62 (2.77 to 4.48)	14.14 (11.35 to 16.85)	0.71 (0.36 to 1.07)	244.58 (198.28 to 291.19)	0.62 (0.23 to 1.02)
Azerbaijan	4.49 (2.72 to 7.05)	0.33 (0.06 to 0.61)	10.22 (5.68 to 16.91)	2.78 (2.31 to 3.25)	3.03 (1.84 to 4.68)	-0.64 (-0.82 to -0.46)	51.37 (30.95 to 79.63)	-0.48 (-0.84 to -0.12)
Bahamas	6.71 (5.33 to 8.38)	1.31 (0.34 to 2.28)	12.45 (8.96 to 17.15)	2.63 (1.69 to 3.59)	4.97 (4.01 to 6.09)	0.8 (-0.34 to 1.96)	82.02 (65.93 to 101.68)	0.68 (-0.13 to 1.5)
Bahrain	7.52 (3.69 to 12.36)	2.58 (1.74 to 3.43)	29.79 (14.06 to 50.22)	6.78 (5.94 to 7.64)	1.1 (0.55 to 1.75)	-0.74 (-1.5 to 0.03)	19.16 (9.47 to 30.89)	-0.5 (-1.07 to 0.08)
Bangladesh	1.26 (0.63 to 2.35)	0.68 (0.34 to 1.02)	1.62 (0.77 to 3.19)	2.9 (2.43 to 3.37)	1.03 (0.52 to 1.88)	-0.12 (-0.48 to 0.23)	17.55 (8.62 to 32.46)	-0.25 (-0.57 to 0.07)
Barbados	5.49 (4.23 to 6.96)	1.87	15.36 (10.85 to 21)	4.12	3.51 (2.79 to 4.32)	1.03	55.58 (43.7 to 69.03)	0.97

© 2025 Wang R, et al. *JAMA Dermatol*.

		(1.57 to 2.17)		(3.81 to 4.42)		(0.69 to 1.36)		(0.64 to 1.31)
Belarus	39.53 (29.21 to 52.64)	5.36 (5.18 to 5.55)	244.51 (178.6 to 331.98)	6.8 (5.69 to 7.93)	11.34 (8.82 to 14.3)	3.1 (1.7 to 4.51)	212.87 (165.08 to 269.87)	3.27 (1.92 to 4.65)
Belgium	48.57 (36.2 to 62.85)	2.43 (1.92 to 2.95)	350.21 (260.15 to 451.84)	3.38 (2.81 to 3.95)	10.26 (8.14 to 12.47)	0.6 (0.25 to 0.96)	189.79 (152.72 to 230.05)	0.73 (0.23 to 1.23)
Belize	2.39 (1.97 to 2.84)	1.93 (0.38 to 3.49)	3.65 (2.75 to 4.8)	3.12 (1.57 to 4.69)	1.9 (1.58 to 2.21)	1.51 (0.17 to 2.86)	30.98 (25.87 to 36.12)	1.47 (0.06 to 2.89)
Benin	1.44 (0.53 to 2.36)	1.12 (0.95 to 1.28)	1.18 (0.42 to 1.99)	1.73 (1.53 to 1.94)	1.41 (0.52 to 2.31)	0.87 (0.74 to 1)	23.27 (8.62 to 37.96)	0.7 (0.59 to 0.82)
Bermuda	38.28 (27.71 to 51.58)	1.77 (0.91 to 2.64)	222.44 (157.18 to 306.52)	4.73 (2.68 to 6.82)	13.13 (10.06 to 16.88)	-0.26 (-0.72 to 0.19)	210.01 (161.12 to 271.57)	-0.52 (-1.37 to 0.33)
Bhutan	1.33 (0.71 to 2.53)	0.75 (0.67 to 0.82)	1.64 (0.84 to 3.35)	2.73 (2.54 to 2.93)	1.12 (0.61 to 2.09)	0.02 (-0.09 to 0.13)	19.25 (10.3 to 35.97)	-0.08 (-0.19 to 0.03)
Bolivia (Plurinational State of)	9.86 (5.49 to 16.52)	0.63 (0.51 to 0.75)	10.04 (5.52 to 17.79)	2.11 (2 to 2.21)	8.94 (5.03 to 15)	0.04 (-0.03 to 0.11)	145.23 (80.8 to 245.62)	-0.02 (-0.1 to 0.06)
Bosnia and Herzegovina	21.85 (11.22 to 31.02)	2.67 (2.38 to 2.95)	102.46 (51.34 to 149.85)	5.44 (4.93 to 5.95)	9.5 (4.95 to 13.01)	1 (0.74 to 1.26)	165.76 (86.71 to 227.33)	0.99 (0.82 to 1.16)

© 2025 Wang R, et al. *JAMA Dermatol*.

Botswana	8.72 (5.07 to 14.73)	0.26 (-0.03 to 0.56)	7.81 (4.42 to 13.86)	1.08 (0.83 to 1.34)	8.07 (4.73 to 13.39)	-0.11 (-0.43 to 0.22)	133.01 (77.59 to 221.64)	-0.14 (-0.47 to 0.19)
Brazil	9.04 (7.81 to 9.99)	1.71 (1.06 to 2.37)	19.64 (16.94 to 22.55)	4.32 (3.62 to 5.02)	6.19 (5.37 to 6.82)	0.76 (0.07 to 1.45)	102.04 (90.33 to 111.53)	0.67 (-0.06 to 1.42)
Brunei Darussalam	4.27 (2.59 to 6.23)	0.6 (0.14 to 1.06)	12.2 (7.04 to 18.78)	3 (2.69 to 3.32)	2.68 (1.65 to 3.87)	-0.28 (-0.82 to 0.26)	45.3 (27.83 to 65.98)	-0.45 (-0.88 to -0.02)
Bulgaria	22.22 (17.33 to 28.22)	2.68 (1.47 to 3.92)	98.91 (72.9 to 129.51)	4.59 (4.06 to 5.13)	9.67 (7.81 to 11.64)	1.26 (0.44 to 2.08)	162.72 (131.88 to 196.13)	1.51 (0.71 to 2.31)
Burkina Faso	1.72 (0.69 to 2.77)	0.91 (0.61 to 1.21)	1.39 (0.55 to 2.28)	1.39 (1.07 to 1.7)	1.74 (0.7 to 2.8)	0.78 (0.56 to 0.99)	28.44 (11.51 to 45.81)	0.63 (0.43 to 0.82)
Burundi	4.63 (2.74 to 6.88)	-0.61 (-0.71 to -0.51)	3.34 (1.99 to 5.02)	-0.22 (-0.39 to -0.06)	4.75 (2.81 to 7.06)	-0.76 (-0.84 to -0.67)	84.42 (49.7 to 125.6)	-0.83 (-0.93 to -0.73)
Cabo Verde	3.28 (0.48 to 6.17)	6.71 (6.41 to 7.01)	6.75 (1.06 to 13.68)	9.44 (9.05 to 9.82)	2.42 (0.34 to 4.51)	5.95 (5.68 to 6.22)	37.31 (5.69 to 69.19)	5.8 (5.53 to 6.07)
Cambodia	1.72 (1.02 to 2.67)	1.61 (1.55 to 1.67)	1.1 (0.65 to 1.72)	1.85 (1.77 to 1.94)	1.91 (1.12 to 2.96)	1.5 (1.45 to 1.56)	31.11 (18.27 to 48.41)	1.34 (1.29 to 1.4)
Cameroon	1.78 (0.69 to 2.96)	1.05 (0.95 to	1.53 (0.59 to 2.63)	1.74 (1.61 to 1.87)	1.7 (0.65 to 2.78)	0.82 (0.71 to 0.94)	28.19 (10.89 to 46.17)	0.68 (0.58 to 0.78)

© 2025 Wang R, et al. *JAMA Dermatol*.

		1.14)						
Canada	54.99 (41.58 to 71.14)	1.88 (1.35 to 2.42)	396.17 (298.43 to 511.75)	2.43 (1.85 to 3.02)	11.12 (9.01 to 13.31)	0.45 (-0.02 to 0.92)	199.54 (161.59 to 239.1)	0.36 (-0.13 to 0.85)
Central African Republic	2.86 (1.71 to 4.55)	-0.05 (-0.14 to 0.04)	1.81 (1.09 to 2.92)	0.06 (-0.05 to 0.17)	3.14 (1.88 to 4.96)	-0.09 (-0.18 to 0)	53.48 (32.6 to 84.64)	-0.12 (-0.2 to -0.03)
Chad	1.33 (0.64 to 2.16)	1.19 (1.08 to 1.31)	0.93 (0.45 to 1.55)	1.42 (1.35 to 1.49)	1.38 (0.65 to 2.24)	1.11 (1.06 to 1.17)	23.25 (11.16 to 38.02)	1.01 (0.95 to 1.07)
Chile	14.99 (11.72 to 18.8)	2.35 (1.81 to 2.89)	76.81 (57.97 to 100.12)	6.28 (5.31 to 7.25)	5.9 (4.81 to 7.1)	0.21 (-0.32 to 0.75)	100.83 (82.94 to 120.84)	0.14 (-0.49 to 0.77)
China	2.86 (1.48 to 3.81)	1.87 (1.52 to 2.22)	11.08 (5.64 to 15.11)	7.24 (6.85 to 7.64)	1.65 (0.85 to 2.18)	-0.03 (-0.47 to 0.42)	26.37 (13.63 to 34.87)	-0.06 (-0.45 to 0.33)
Colombia	9.35 (7.24 to 11.98)	1.88 (1.57 to 2.19)	31.38 (22.08 to 43.09)	5.91 (5.49 to 6.33)	5.3 (4.15 to 6.61)	0.35 (-0.09 to 0.8)	88.86 (69.58 to 110.84)	0.29 (-0.15 to 0.73)
Comoros	4.62 (2.46 to 7.29)	0.28 (0.11 to 0.44)	3.92 (2.04 to 6.27)	0.95 (0.8 to 1.09)	4.44 (2.37 to 6.94)	0.02 (-0.11 to 0.15)	78.39 (41.36 to 122.91)	-0.09 (-0.24 to 0.06)
Congo	3.71 (2.19 to 5.7)	0.56 (0.48 to 0.64)	3.22 (1.87 to 5.16)	1.51 (1.29 to 1.74)	3.6 (2.14 to 5.49)	0.22 (0.14 to 0.3)	59.8 (36.32 to 91.07)	0.15 (0.07 to 0.24)
Cook Islands	2.16 (1.29 to 3.45)	0.24	1.92 (1.11 to 3.13)	1.11	1.95 (1.17 to 3.08)	-0.23	30.36 (18.59 to 47.47)	-0.3

© 2025 Wang R, et al. *JAMA Dermatol*.

		(-0.34 to 0.83)		(0.55 to 1.68)		(-0.86 to 0.4)		(-0.77 to 0.18)
Costa Rica	14.51 (11.08 to 18.51)	1.77 (1.15 to 2.4)	56.15 (39.86 to 75.34)	4.04 (3.38 to 4.7)	7.5 (5.89 to 9.21)	0.72 (0.14 to 1.31)	123.58 (97.71 to 151.13)	0.66 (0.02 to 1.3)
Croatia	54.23 (41 to 70.41)	2.99 (2.42 to 3.57)	318.31 (236.19 to 422.67)	4.97 (4.26 to 5.69)	17.79 (14.42 to 21.72)	1.46 (1.29 to 1.62)	305.68 (245.68 to 374.33)	1.58 (1.41 to 1.75)
Cuba	7.83 (6.01 to 9.98)	2.5 (2.14 to 2.86)	31.74 (22.91 to 42.88)	4.38 (4.18 to 4.59)	3.91 (3.07 to 4.79)	1.26 (0.92 to 1.61)	64.65 (50.64 to 79.44)	1.42 (1.07 to 1.78)
Cyprus	35.99 (17.56 to 56.78)	1.66 (0.96 to 2.37)	248.87 (122.18 to 393.86)	4.17 (3.66 to 4.68)	8.02 (3.98 to 12.25)	-1.28 (-1.97 to -0.59)	141.3 (72.15 to 213.87)	-0.78 (-1.36 to -0.21)
Czechia	72.04 (53.49 to 93.09)	3.12 (2.83 to 3.42)	492.61 (365.66 to 639.37)	4.75 (4.4 to 5.1)	15.75 (12.75 to 19.15)	0.69 (0.05 to 1.33)	285.15 (230.67 to 348.45)	0.6 (0.06 to 1.14)
Côte d'Ivoire	2.44 (1.41 to 3.99)	0.52 (0.38 to 0.66)	2.14 (1.2 to 3.52)	1.27 (1.16 to 1.37)	2.29 (1.33 to 3.71)	0.18 (0.02 to 0.34)	39.79 (22.85 to 63.45)	0.15 (-0.01 to 0.31)
Democratic People's Republic of Korea	1.62 (0.98 to 2.6)	0.34 (0.29 to 0.4)	1.04 (0.63 to 1.68)	0.45 (0.35 to 0.56)	1.75 (1.04 to 2.85)	0.27 (0.2 to 0.34)	28.71 (17.45 to 46.04)	0.24 (0.21 to 0.28)
Democratic Republic of the Congo	3.19 (1.75 to 5.14)	0.62 (0.56 to 0.69)	2.49 (1.36 to 4.11)	1.22 (1.12 to 1.33)	3.24 (1.75 to 5.32)	0.4 (0.35 to 0.46)	54.33 (30.3 to 88.05)	0.36 (0.3 to 0.42)
Denmark	84.88 (64.98 to 108.53)	3.42 (2.76 to	604.49 (459.19 to 771.99)	4.45 (3.72 to 5.19)	17.58 (14.31 to 21.04)	1.25 (0.82 to 1.68)	321.32 (262.42 to 386.48)	1.28 (0.76 to 1.8)

© 2025 Wang R, et al. *JAMA Dermatol*.

		4.09)						
Djibouti	4.82 (2.48 to 7.83)	0.31 (0.27 to 0.36)	4.2 (2.14 to 6.91)	1.03 (0.92 to 1.14)	4.56 (2.35 to 7.36)	0.05 (0 to 0.11)	80.86 (41.3 to 131.67)	0 (-0.04 to 0.04)
Dominica	3.04 (1.73 to 4.55)	0.13 (-0.17 to 0.44)	3.84 (2.07 to 5.98)	0.91 (0.65 to 1.17)	2.55 (1.46 to 3.79)	-0.19 (-0.46 to 0.07)	41.69 (24.01 to 62.11)	-0.15 (-0.38 to 0.08)
Dominican Republic	1.36 (0.81 to 2.18)	0.38 (-0.01 to 0.78)	1.86 (1.05 to 3.15)	2.22 (1.79 to 2.66)	1.14 (0.68 to 1.82)	-0.28 (-0.65 to 0.08)	18.27 (11 to 29.14)	-0.28 (-0.63 to 0.08)
Ecuador	10.58 (7.94 to 13.69)	2.06 (0.65 to 3.49)	18.53 (12.6 to 26.33)	4.88 (3.5 to 6.29)	7.87 (5.97 to 10.01)	0.98 (-0.77 to 2.77)	121.93 (92.01 to 156.58)	0.92 (-0.79 to 2.65)
Egypt	1.19 (0.7 to 1.79)	2.58 (2.35 to 2.8)	2.12 (1.22 to 3.38)	5.44 (5.02 to 5.86)	0.34 (0.2 to 0.49)	0.05 (-0.23 to 0.34)	5.52 (3.31 to 7.97)	0.3 (-0.07 to 0.67)
El Salvador	1.87 (0.98 to 2.64)	2.07 (1.52 to 2.61)	4.64 (2.3 to 6.99)	5.94 (5.55 to 6.34)	1.25 (0.65 to 1.74)	0.7 (0.23 to 1.19)	20.64 (10.85 to 28.84)	0.71 (0.21 to 1.22)
Equatorial Guinea	3.9 (1.96 to 6.5)	1.25 (1.09 to 1.41)	5.22 (2.46 to 9.41)	3.75 (3.54 to 3.95)	3.36 (1.68 to 5.59)	0.42 (0.38 to 0.47)	55.64 (28.13 to 91.82)	0.31 (0.14 to 0.49)
Eritrea	5.46 (3.28 to 8.37)	0.38 (0.31 to 0.45)	3.91 (2.32 to 6.08)	0.78 (0.72 to 0.85)	5.53 (3.3 to 8.48)	0.2 (0.14 to 0.27)	98.22 (58.63 to 150.38)	0.09 (0.03 to 0.15)
Estonia	46.14 (34.87 to 59.52)	3.37 (2.22 to	286.94 (213.37 to 378.34)	5.42 (4.14 to 6.71)	13.02 (10.31 to 15.76)	1.3 (0.54 to 2.07)	231.34 (183.31 to 281.69)	1.36 (0.52 to 2.22)

© 2025 Wang R, et al. *JAMA Dermatol*.

		4.52)						
Eswatini	10.01 (6.05 to 17.15)	0.57 (0.51 to 0.62)	7.9 (4.64 to 13.74)	0.97 (0.88 to 1.06)	9.59 (5.89 to 16.06)	0.36 (0.29 to 0.44)	162.92 (100.2 to 275.17)	0.39 (0.31 to 0.48)
Ethiopia	2.75 (1.74 to 3.76)	0.12 (0.05 to 0.19)	2.33 (1.46 to 3.26)	1.17 (1.07 to 1.27)	2.66 (1.67 to 3.63)	-0.29 (-0.37 to -0.21)	46.74 (29.36 to 64.13)	-0.42 (-0.5 to -0.34)
Fiji	2.53 (1.31 to 5.35)	0.78 (0.28 to 1.28)	1.48 (0.77 to 3.1)	0.8 (0.37 to 1.24)	3.11 (1.6 to 6.61)	0.99 (0.46 to 1.53)	42.19 (22.16 to 87)	0.69 (0.25 to 1.13)
Finland	68.68 (51.35 to 89.01)	3.12 (2.67 to 3.57)	508.52 (381.13 to 659.25)	4.24 (3.95 to 4.53)	12.9 (10.22 to 15.63)	0.86 (0.33 to 1.4)	243.18 (194.28 to 295.69)	1.03 (0.5 to 1.57)
France	54.23 (41.48 to 68.58)	3.05 (2.47 to 3.64)	418.49 (322.05 to 529.41)	4.03 (3.44 to 4.63)	8.73 (7.05 to 10.45)	0.14 (-0.71 to 1.01)	169.3 (136.97 to 204.93)	0.49 (-0.3 to 1.29)
Gabon	4.25 (2.37 to 6.67)	0.47 (0.43 to 0.51)	4.58 (2.47 to 7.53)	1.88 (1.7 to 2.06)	3.84 (2.14 to 6.01)	-0.01 (-0.11 to 0.09)	63.36 (35.7 to 98.41)	-0.07 (-0.12 to -0.01)
Gambia	1.38 (0.82 to 2.16)	0.94 (0.46 to 1.41)	1.22 (0.71 to 1.94)	1.57 (1 to 2.15)	1.28 (0.76 to 2.02)	0.66 (0.22 to 1.11)	22.5 (13.23 to 35.05)	0.65 (0.1 to 1.2)
Georgia	16.28 (13.16 to 19.79)	0.21 (-1.04 to 1.47)	45.76 (33.48 to 60.07)	0.85 (-0.23 to 1.93)	10.19 (8.51 to 11.94)	-0.23 (-1.51 to 1.06)	169.21 (142.07 to 198.54)	-0.26 (-1.42 to 0.91)
Germany	65.27 (49.59 to 82.49)	3.35	475.14 (362.24 to 602.6)	4.14	11.94 (9.66 to 14.3)	0.85	220.73 (179.93 to	0.96

© 2025 Wang R, et al. *JAMA Dermatol*.

		(2.61 to 4.09)		(3.15 to 5.13)		(0.04 to 1.66)	265.62)	(0.27 to 1.66)
Ghana	0.18 (0.09 to 0.32)	0.85 (0.76 to 0.94)	0.19 (0.09 to 0.35)	2.02 (1.77 to 2.28)	0.16 (0.08 to 0.28)	0.46 (0.38 to 0.55)	2.72 (1.38 to 4.74)	0.41 (0.33 to 0.49)
Greece	30.43 (23.98 to 37.83)	2.66 (2.44 to 2.89)	211.13 (165.62 to 262.67)	3.18 (2.6 to 3.77)	7.37 (6.31 to 8.29)	1.56 (0.85 to 2.28)	134.82 (116.12 to 152.68)	1.74 (1.07 to 2.42)
Greenland	9.41 (5.25 to 14.62)	0.23 (-0.19 to 0.65)	25.44 (13.49 to 41.58)	2.75 (1.93 to 3.57)	5.49 (3.09 to 8.43)	-0.72 (-1.21 to -0.24)	96.84 (54.7 to 148.99)	-0.77 (-1.23 to -0.3)
Grenada	5.52 (4.34 to 6.93)	2.36 (0.03 to 4.75)	8.98 (6.39 to 12.34)	4.03 (2.5 to 5.58)	4.19 (3.35 to 5.18)	1.65 (0.05 to 3.28)	70.02 (56.08 to 86.42)	1.69 (0 to 3.41)
Guam	0.5 (0.31 to 0.79)	-1.31 (-2.43 to -0.17)	0.41 (0.25 to 0.65)	-1.12 (-2.41 to 0.18)	0.47 (0.29 to 0.74)	-1.53 (-2.97 to -0.06)	8.07 (5.07 to 12.74)	-1.3 (-2.49 to -0.09)
Guatemala	2.62 (2.15 to 3.13)	1.14 (-0.31 to 2.62)	2.98 (2.26 to 3.87)	3.13 (1.17 to 5.12)	2.3 (1.9 to 2.71)	0.4 (-0.94 to 1.75)	36.62 (30.19 to 43.45)	0.47 (-1.04 to 1.99)
Guinea	4.88 (2.85 to 7.63)	0.45 (0.38 to 0.52)	3.45 (2.01 to 5.38)	0.76 (0.69 to 0.83)	4.96 (2.9 to 7.81)	0.28 (0.19 to 0.36)	87.08 (50.91 to 136)	0.29 (0.24 to 0.33)
Guinea-Bissau	1.75 (0.77 to 2.98)	1.29 (1.22 to 1.37)	1.21 (0.53 to 2.09)	1.63 (1.57 to 1.68)	1.82 (0.79 to 3.07)	1.13 (1.05 to 1.22)	30.42 (13.61 to 51.49)	0.98 (0.92 to 1.03)

© 2025 Wang R, et al. *JAMA Dermatol*.

Guyana	2.12 (1.55 to 2.8)	1.44 (0.16 to 2.74)	1.97 (1.41 to 2.7)	2.3 (1.04 to 3.58)	1.97 (1.44 to 2.59)	1.13 (-0.1 to 2.37)	33.38 (24.52 to 44.09)	1.14 (-0.24 to 2.54)
Haiti	3.92 (2.12 to 6.74)	0.27 (0.2 to 0.33)	2.6 (1.37 to 4.53)	0.59 (0.37 to 0.82)	4.19 (2.24 to 7.2)	0.13 (0.04 to 0.21)	69.21 (36.61 to 120.83)	0.09 (0.05 to 0.14)
Honduras	1.96 (1.19 to 2.87)	1.48 (1.31 to 1.66)	1.98 (1.18 to 3)	2.68 (2.38 to 2.98)	1.77 (1.09 to 2.6)	1.03 (0.8 to 1.27)	29.46 (18.07 to 43)	1.05 (0.82 to 1.28)
Hungary	33.21 (25.11 to 42.85)	2.48 (2.27 to 2.69)	187.6 (136.95 to 247.97)	4.87 (4.64 to 5.1)	11.7 (9.38 to 14.26)	0.32 (0.03 to 0.6)	208.49 (167.33 to 254.91)	0.42 (0.14 to 0.71)
Iceland	68.71 (50.19 to 90.42)	2.6 (1.61 to 3.6)	513.87 (376.09 to 676.26)	3.13 (2.23 to 4.05)	11.88 (9.22 to 14.63)	0.96 (0.34 to 1.59)	219.05 (172.2 to 271.15)	0.99 (0.24 to 1.75)
India	1.33 (0.85 to 1.72)	1.63 (1.4 to 1.87)	1.79 (1.08 to 2.29)	3.71 (3.3 to 4.13)	1.08 (0.7 to 1.41)	0.88 (0.59 to 1.17)	18.93 (12.3 to 24.78)	0.81 (0.55 to 1.08)
Indonesia	1.14 (0.73 to 1.51)	1.13 (1.08 to 1.18)	0.72 (0.47 to 0.96)	1.3 (1.19 to 1.41)	1.24 (0.79 to 1.66)	1.06 (0.99 to 1.13)	19.92 (12.94 to 26.55)	0.89 (0.83 to 0.94)
Iran (Islamic Republic of)	19.2 (9.67 to 26.52)	1.69 (1.1 to 2.28)	68.87 (34.28 to 100.46)	5.48 (4.95 to 6)	2.99 (1.5 to 3.88)	-1.4 (-1.48 to -1.33)	50.79 (26.31 to 66.13)	-1.18 (-1.27 to -1.1)
Iraq	4.79 (2.77 to 8.05)	2.84 (2.44 to 3.24)	12.61 (6.79 to 21.78)	6.03 (5.53 to 6.52)	1.01 (0.62 to 1.68)	0.28 (0.08 to 0.48)	17.96 (10.87 to 29.16)	0.3 (0.13 to 0.48)
Ireland	60.08 (45.45 to 78.01)	2.63 (1.58 to	442.5 (334.97 to 575.26)	3.82 (2.84 to 4.81)	11.13 (8.75 to 13.44)	0.43 (0.03 to 0.83)	202.76 (160.94 to 245.59)	0.53 (0.11 to 0.95)

© 2025 Wang R, et al. *JAMA Dermatol*.

		3.69)						
Israel	52.35 (39.18 to 67.45)	1.92 (0.75 to 3.09)	355.73 (266.48 to 459.02)	3.31 (2.19 to 4.44)	13.75 (10.85 to 16.65)	0.03 (-0.98 to 1.05)	235.58 (188.08 to 285.46)	-0.04 (-0.47 to 0.4)
Italy	43.88 (34.51 to 53.58)	2.55 (2.22 to 2.87)	301.68 (236.84 to 372.3)	3.93 (3.65 to 4.22)	9.95 (8.52 to 10.94)	0.7 (0.41 to 0.99)	178.63 (156.1 to 196.86)	0.75 (0.49 to 1)
Jamaica	4.64 (3.34 to 6.22)	2.58 (-0.07 to 5.3)	10.58 (7.02 to 15.61)	3.95 (2.46 to 5.45)	3.18 (2.33 to 4.2)	2 (-0.66 to 4.74)	53.22 (38.81 to 70.39)	2.31 (-0.26 to 4.94)
Japan	6.82 (5.26 to 8.4)	1.81 (0.88 to 2.75)	50.22 (38.78 to 62.35)	2.64 (1.69 to 3.6)	1.42 (1.18 to 1.55)	0.06 (-0.66 to 0.79)	25.14 (21.47 to 27.79)	0.22 (-0.34 to 0.79)
Jordan	3.53 (1.77 to 5.78)	2.57 (1.95 to 3.19)	13.9 (6.27 to 23.89)	6.14 (5.76 to 6.53)	0.55 (0.29 to 0.85)	-0.57 (-0.93 to -0.21)	9.73 (4.94 to 15.3)	-0.37 (-0.78 to 0.04)
Kazakhstan	9.63 (7.7 to 11.89)	1.86 (1.03 to 2.69)	30.12 (21.82 to 39.96)	4.42 (2.83 to 6.03)	5.86 (4.84 to 6.91)	0.92 (-0.27 to 2.13)	92.59 (76.58 to 109.57)	0.77 (0.31 to 1.24)
Kenya	2.6 (1.57 to 3.69)	0.79 (0.61 to 0.97)	2.73 (1.6 to 3.97)	1.85 (1.64 to 2.07)	2.43 (1.48 to 3.43)	0.59 (0.45 to 0.73)	42.51 (25.84 to 60.16)	0.55 (0.42 to 0.68)
Kiribati	0.44 (0.14 to 0.76)	0.38 (0.33 to 0.42)	0.25 (0.08 to 0.42)	0.36 (0.31 to 0.41)	0.54 (0.17 to 0.96)	0.45 (0.4 to 0.5)	7.85 (2.76 to 13.41)	0.24 (0.17 to 0.3)
Kuwait	3.41 (2.52 to 4.48)	2.36	17.91 (12.83 to 24.09)	4	0.43 (0.32 to 0.55)	0.93	7.5 (5.52 to 9.98)	0.79

© 2025 Wang R, et al. *JAMA Dermatol*.

		(-3.3 to 8.35)		(-1.56 to 9.88)		(-4.97 to 7.2)		(-4.45 to 6.32)
Kyrgyzstan	5.35 (3.98 to 6.96)	-0.03 (-1.02 to 0.97)	12.73 (8.78 to 17.73)	1.63 (-0.14 to 3.43)	3.69 (2.78 to 4.74)	-0.64 (-1.63 to 0.35)	60.11 (45.65 to 77.08)	-1.16 (-2.33 to 0.02)
Lao People's Democratic Republic	1.5 (0.88 to 2.39)	0.7 (0.65 to 0.76)	0.9 (0.52 to 1.44)	0.82 (0.77 to 0.86)	1.7 (0.99 to 2.73)	0.62 (0.57 to 0.67)	27.79 (16.23 to 45.01)	0.46 (0.43 to 0.5)
Latvia	38.37 (29.23 to 49.52)	4.06 (3.1 to 5.03)	225.95 (167.17 to 299.3)	5.59 (4.65 to 6.54)	12.67 (10.19 to 15.43)	2.42 (1.64 to 3.22)	227.5 (183.23 to 280)	2.56 (1.73 to 3.39)
Lebanon	11.04 (5.99 to 19.12)	3.28 (3.03 to 3.53)	52.49 (27.88 to 90.88)	7.06 (6.67 to 7.45)	1.49 (0.83 to 2.56)	0.02 (-0.25 to 0.29)	26.93 (15.17 to 45.66)	0.21 (-0.05 to 0.48)
Lesotho	9.18 (5.57 to 15.98)	0.93 (0.7 to 1.15)	6.42 (3.83 to 11.48)	0.98 (0.79 to 1.17)	9.37 (5.72 to 16.33)	0.89 (0.67 to 1.11)	160.09 (97.57 to 280.85)	0.91 (0.67 to 1.15)
Liberia	1.55 (0.62 to 2.61)	1.19 (0.94 to 1.44)	1.4 (0.55 to 2.48)	2.28 (1.96 to 2.61)	1.46 (0.58 to 2.44)	0.8 (0.62 to 0.99)	24.4 (9.77 to 40.9)	0.71 (0.52 to 0.9)
Libya	2.35 (1.28 to 3.94)	2.03 (1.3 to 2.78)	7.24 (3.78 to 12.1)	4.78 (4.12 to 5.45)	0.51 (0.29 to 0.87)	-0.02 (-0.28 to 0.23)	9.18 (5.23 to 15.14)	0.13 (-0.07 to 0.33)
Lithuania	47.38 (35.52 to 62.47)	4.89 (3.57 to 6.23)	285.45 (207.72 to 385.76)	6.06 (4.73 to 7.41)	12.66 (10.22 to 15.58)	3.46 (2.01 to 4.93)	232.64 (187.07 to 286.79)	3.59 (2.1 to 5.1)
Luxembourg	53.02 (41.47 to 66.48)	2.44 (1.93 to 2.96)	386.19 (300.38 to 487.96)	3.69 (3.28 to 4.1)	10.82 (9.12 to 12.47)	0.08 (-0.34 to 0.5)	197.48 (166.73 to 229.76)	0.18 (-0.03 to 0.39)

© 2025 Wang R, et al. *JAMA Dermatol*.

Madagascar	3.93 (2.17 to 6.41)	-0.44 (-0.64 to -0.23)	3.04 (1.66 to 4.88)	0.01 (-0.25 to 0.27)	3.89 (2.13 to 6.37)	-0.6 (-0.77 to -0.44)	69.5 (37.87 to 114.54)	-0.66 (-0.83 to -0.5)
Malawi	9.84 (5.3 to 15.9)	0.67 (0.53 to 0.81)	8.14 (4.29 to 13.53)	1.4 (1.18 to 1.61)	9.48 (5.12 to 15.06)	0.4 (0.27 to 0.53)	168.86 (90.48 to 267.25)	0.35 (0.25 to 0.44)
Malaysia	2.38 (1.31 to 3.68)	0.31 (0.19 to 0.43)	1.94 (1.03 to 3.06)	1.02 (0.49 to 1.56)	2.27 (1.25 to 3.51)	0.04 (-0.1 to 0.17)	38.55 (21.07 to 59.65)	-0.17 (-0.27 to -0.06)
Maldives	0.54 (0.26 to 0.88)	-0.87 (-1.28 to -0.47)	0.48 (0.23 to 0.8)	0.42 (-0.15 to 1)	0.5 (0.23 to 0.82)	-1.45 (-1.83 to -1.08)	7.76 (3.7 to 12.69)	-1.54 (-2.14 to -0.94)
Mali	3.95 (2.14 to 6.97)	0.29 (0.19 to 0.4)	3.1 (1.65 to 5.6)	0.84 (0.72 to 0.96)	3.86 (2.09 to 6.73)	0.04 (-0.07 to 0.16)	67.85 (36.1 to 115.83)	0.01 (-0.08 to 0.11)
Malta	28.87 (21.34 to 38.1)	2.14 (1.74 to 2.54)	208.63 (153.08 to 277.29)	3.34 (2.92 to 3.76)	6.31 (4.95 to 7.71)	0 (-0.37 to 0.37)	115.99 (91.59 to 142.96)	0.25 (-0.02 to 0.52)
Marshall Islands	2.07 (1.17 to 3.46)	0.54 (0.42 to 0.66)	1.21 (0.69 to 2.04)	0.58 (0.45 to 0.7)	2.38 (1.35 to 3.97)	0.51 (0.4 to 0.63)	38.11 (21.77 to 63.62)	0.45 (0.33 to 0.56)
Mauritania	1.73 (0.68 to 2.88)	1.24 (1.13 to 1.36)	2.07 (0.77 to 3.63)	3.1 (2.97 to 3.24)	1.48 (0.58 to 2.44)	0.57 (0.47 to 0.67)	24.6 (9.79 to 40.75)	0.45 (0.26 to 0.63)
Mauritius	1.33 (1.14 to 1.52)	7.59 (4.32 to 10.96)	1.1 (0.92 to 1.31)	8.07 (4.55 to 11.7)	1.27 (1.1 to 1.45)	7.33 (3.93 to 10.85)	21.24 (18.55 to 24.07)	7.33 (4.04 to 10.72)

© 2025 Wang R, et al. *JAMA Dermatol*.

Mexico	7.07 (6.22 to 7.92)	2.3 (2.06 to 2.54)	13.62 (11.9 to 15.43)	4.91 (4.17 to 5.65)	5.07 (4.49 to 5.64)	1.17 (0.9 to 1.44)	81.57 (72.25 to 91.34)	1.4 (1.15 to 1.65)
Micronesia (Federated States of)	1.86 (1.09 to 3.03)	-0.06 (-0.09 to -0.02)	1.11 (0.65 to 1.81)	0.02 (-0.01 to 0.05)	2.12 (1.23 to 3.47)	-0.12 (-0.15 to -0.09)	33.95 (19.91 to 55.43)	-0.17 (-0.19 to -0.14)
Monaco	49.33 (26.26 to 75.64)	2.18 (2.04 to 2.32)	350.66 (184.79 to 539.83)	2.52 (2.42 to 2.62)	9.92 (5.53 to 14.46)	0.96 (0.79 to 1.13)	181.99 (101.61 to 263.02)	1 (0.83 to 1.17)
Mongolia	3.49 (1.93 to 5.85)	0.19 (-0.16 to 0.53)	5 (2.72 to 8.64)	2.45 (2.14 to 2.76)	2.83 (1.57 to 4.67)	-0.61 (-1 to -0.22)	44.44 (24.91 to 73.22)	-0.72 (-1.12 to -0.31)
Montenegro	27.02 (16.29 to 38.63)	1.75 (1.18 to 2.32)	145.32 (83.87 to 212.01)	2.05 (1.67 to 2.44)	9.92 (6.2 to 13.8)	1.06 (0.62 to 1.51)	165.39 (101.74 to 229.53)	0.85 (0.53 to 1.17)
Morocco	6.39 (2.09 to 11.09)	2.73 (2.54 to 2.92)	10.76 (3.33 to 18.99)	5.32 (5.17 to 5.46)	2.06 (0.66 to 3.56)	0.63 (0.49 to 0.76)	33.71 (11.13 to 57.82)	0.68 (0.54 to 0.83)
Mozambique	5.52 (3.06 to 8.68)	0.44 (0.38 to 0.51)	4.05 (2.21 to 6.42)	0.82 (0.7 to 0.94)	5.61 (3.1 to 8.79)	0.27 (0.18 to 0.37)	99.78 (54.84 to 156.06)	0.26 (0.2 to 0.33)
Myanmar	1.3 (0.77 to 1.98)	0.72 (0.7 to 0.75)	0.82 (0.49 to 1.25)	0.98 (0.91 to 1.06)	1.44 (0.85 to 2.21)	0.61 (0.59 to 0.64)	23.49 (14.04 to 36.35)	0.47 (0.45 to 0.5)
Namibia	18.65 (11.33 to 32.94)	1.26 (1.18 to 1.34)	20.27 (11.87 to 35.81)	2.63 (2.48 to 2.79)	16.28 (9.99 to 28.52)	0.76 (0.66 to 0.86)	281.22 (174.36 to 476.13)	0.74 (0.66 to 0.83)

© 2025 Wang R, et al. *JAMA Dermatol*.

Nauru	2.12 (1.12 to 3.69)	-0.3 (-0.33 to -0.27)	1.26 (0.67 to 2.21)	-0.24 (-0.27 to -0.21)	2.41 (1.26 to 4.15)	-0.31 (-0.34 to -0.28)	38.25 (20.1 to 66.63)	-0.37 (-0.4 to -0.33)
Nepal	1.24 (0.73 to 2.22)	1.07 (1 to 1.14)	1.33 (0.75 to 2.51)	2.71 (2.56 to 2.87)	1.1 (0.65 to 1.95)	0.46 (0.4 to 0.52)	18.94 (11.2 to 33.64)	0.4 (0.35 to 0.46)
Netherlands	92.75 (71.01 to 118.85)	3.8 (3.45 to 4.16)	675.51 (517.96 to 865.43)	4.54 (4.14 to 4.93)	18.45 (14.84 to 22.18)	1.89 (1.57 to 2.21)	337.1 (273.21 to 404.45)	1.94 (1.61 to 2.26)
New Zealand	202.21 (152.23 to 262.44)	1.71 (1.31 to 2.12)	1495.67 (1127.78 to 1935.18)	2.11 (1.12 to 3.11)	30.49 (24.55 to 36.8)	0.37 (-0.33 to 1.07)	560.61 (452.38 to 680.46)	0.32 (-0.36 to 1.01)
Nicaragua	2.62 (1.43 to 3.88)	1.49 (1.18 to 1.81)	5.97 (3.13 to 9.3)	4.63 (4.33 to 4.92)	1.87 (0.99 to 2.74)	0.5 (0.15 to 0.86)	31.51 (16.94 to 46.22)	0.69 (0.42 to 0.96)
Niger	1.31 (0.57 to 2.12)	1.03 (0.93 to 1.13)	0.94 (0.41 to 1.55)	1.4 (1.28 to 1.52)	1.35 (0.58 to 2.16)	0.87 (0.75 to 0.99)	22.43 (9.68 to 36.22)	0.76 (0.64 to 0.88)
Nigeria	3.76 (1.63 to 5.3)	0.77 (0.66 to 0.88)	2.83 (1.18 to 4.1)	1.28 (1.18 to 1.39)	3.56 (1.61 to 4.96)	0.44 (0.36 to 0.52)	60.46 (26.09 to 85.61)	0.38 (0.31 to 0.45)
Niue	2.43 (1.45 to 3.92)	0.47 (0.41 to 0.53)	1.6 (0.96 to 2.6)	0.61 (0.54 to 0.68)	2.6 (1.52 to 4.25)	0.37 (0.29 to 0.44)	40.57 (24.1 to 66.64)	0.27 (0.21 to 0.33)
North Macedonia	44.78 (25.53 to 63.48)	1.74 (1.51 to 1.97)	177.14 (100.59 to 261.1)	4.55 (4.26 to 4.84)	21.9 (12.63 to 30.47)	0.4 (0.04 to 0.77)	356.43 (209.28 to 500.35)	0.25 (0.12 to 0.39)

© 2025 Wang R, et al. *JAMA Dermatol*.

Northern Mariana Islands	1.71 (1.01 to 2.65)	0.58 (0.15 to 1.01)	1.28 (0.75 to 1.99)	0.66 (0.16 to 1.16)	1.65 (1 to 2.57)	0.55 (0.21 to 0.9)	27.14 (16.36 to 41.89)	0.55 (0.13 to 0.98)
Norway	110.53 (87.41 to 135.39)	2.55 (2 to 3.11)	793.03 (628.46 to 975.36)	3.4 (3.03 to 3.76)	23.17 (20.07 to 25.62)	0.75 (0.49 to 1.02)	418.12 (367.66 to 463.6)	0.73 (0.45 to 1.02)
Oman	3.12 (1.71 to 5.44)	3 (1.91 to 4.09)	13.1 (7.16 to 22.12)	6.87 (5.98 to 7.77)	0.42 (0.23 to 0.73)	-0.27 (-1.06 to 0.52)	8.34 (4.68 to 14.06)	-0.03 (-0.66 to 0.61)
Pakistan	2.23 (1.4 to 3.4)	0.69 (0.62 to 0.76)	1.6 (0.97 to 2.49)	1.08 (1.02 to 1.14)	2.11 (1.33 to 3.25)	0.39 (0.33 to 0.46)	36.75 (23.13 to 56.19)	0.37 (0.31 to 0.43)
Palau	7.11 (4.07 to 11.08)	-0.84 (-1.18 to -0.5)	4.73 (2.72 to 7.34)	-0.52 (-0.77 to -0.28)	7.93 (4.5 to 12.35)	-1.08 (-1.47 to -0.69)	116.28 (67.06 to 180.03)	-0.81 (-1.08 to -0.55)
Palestine	5.97 (3.5 to 8.91)	1.74 (1.47 to 2.02)	17.01 (9.36 to 27.1)	4.97 (4.62 to 5.32)	1.34 (0.79 to 1.94)	-0.61 (-0.79 to -0.44)	21.93 (13.16 to 31.67)	-0.45 (-0.61 to -0.28)
Panama	8.55 (6.32 to 10.81)	3.01 (2.6 to 3.42)	26.4 (17.96 to 36.46)	5.99 (5.41 to 6.58)	5.12 (3.87 to 6.3)	1.99 (1.61 to 2.37)	84.15 (63.71 to 103.81)	1.96 (1.59 to 2.33)
Papua New Guinea	1.67 (0.95 to 2.83)	0.33 (0.23 to 0.42)	0.99 (0.56 to 1.66)	0.36 (0.26 to 0.45)	1.92 (1.09 to 3.28)	0.34 (0.26 to 0.41)	31.44 (18.02 to 53.5)	0.33 (0.23 to 0.43)
Paraguay	7.06 (3.64 to 10.86)	1.7 (1.45 to 1.94)	12.83 (6.14 to 20.3)	3.79 (3.28 to 4.29)	5.1 (2.59 to 7.83)	0.92 (0.68 to 1.15)	85.19 (43.6 to 130.74)	0.97 (0.73 to 1.21)
Peru	7.71 (4.46 to 11.28)	1.64	21.45 (11.88 to 33.76)	5.96	4.65 (2.72 to 6.76)	-0.03	76.33 (45.16 to 111.42)	0.01

© 2025 Wang R, et al. *JAMA Dermatol*.

		(1.14 to 2.15)		(5.34 to 6.58)		(-0.69 to 0.63)		(-0.66 to 0.69)
Philippines	1.59 (1.01 to 2.23)	0.27 (0.06 to 0.47)	0.97 (0.62 to 1.37)	0.39 (0.19 to 0.6)	1.74 (1.09 to 2.43)	0.17 (0.01 to 0.33)	28.47 (18.26 to 40.21)	0.32 (0.13 to 0.51)
Poland	30.6 (25.67 to 35.79)	3.88 (3.42 to 4.34)	99.03 (75.47 to 126.35)	7.13 (6.83 to 7.43)	17.56 (15.38 to 19.34)	2.47 (1.88 to 3.06)	278.57 (247.04 to 306.44)	2.32 (1.77 to 2.87)
Portugal	26.22 (19.96 to 33.8)	3.43 (2.94 to 3.91)	178.35 (134.75 to 231.37)	5.97 (5.7 to 6.25)	6.66 (5.35 to 8.03)	0.68 (0.32 to 1.04)	118.11 (96.29 to 142.75)	0.88 (0.52 to 1.25)
Puerto Rico	7.08 (5.28 to 9.24)	1.46 (0.32 to 2.62)	35.98 (25.95 to 48.35)	4.14 (2.78 to 5.52)	2.92 (2.24 to 3.69)	-0.14 (-0.69 to 0.41)	48.97 (37.68 to 61.66)	0.07 (-0.48 to 0.62)
Qatar	16.35 (7.85 to 27.35)	0.58 (-0.88 to 2.05)	77.54 (37.57 to 132.29)	4.53 (3.11 to 5.97)	1.98 (0.94 to 3.29)	-2.52 (-3.56 to -1.46)	35.63 (17.32 to 59.73)	-2.11 (-3.26 to -0.95)
Republic of Korea	8.2 (2.8 to 12.4)	3.63 (3.22 to 4.03)	58.19 (19.78 to 87.73)	7.86 (7.37 to 8.36)	1.81 (0.65 to 2.65)	-0.24 (-0.44 to -0.03)	31.38 (11.29 to 45.86)	-0.07 (-0.33 to 0.2)
Republic of Moldova	15.59 (12.73 to 19.16)	2.59 (1.88 to 3.3)	75.17 (57.73 to 96.97)	5.2 (4.36 to 6.04)	6.39 (5.45 to 7.36)	0.91 (-0.06 to 1.89)	121.45 (104.49 to 139.83)	1.06 (0.47 to 1.66)
Romania	17.82 (13.88 to 22.56)	3.39 (2.68 to 4.09)	87.92 (65.85 to 114.18)	6.93 (6.07 to 7.8)	7.76 (6.36 to 9.34)	1.43 (0.89 to 1.97)	135.99 (111.77 to 163.59)	1.63 (1.08 to 2.19)
Russian Federation	24.8 (22.38 to 27.04)	3.83	128.05 (115.57 to 139.62)	5.47	9.92 (8.9 to 10.81)	2.1	179.25 (161.87 to	2.23

© 2025 Wang R, et al. *JAMA Dermatol*.

		(3.14 to 4.53)		(4.65 to 6.3)		(1.55 to 2.66)	195.11)	(1.63 to 2.83)
Rwanda	5.42 (3.08 to 8.3)	-0.19 (-0.28 to -0.11)	4.51 (2.5 to 6.91)	0.58 (0.41 to 0.76)	5.18 (2.96 to 7.83)	-0.59 (-0.65 to -0.53)	91.29 (51.38 to 138.06)	-0.67 (-0.72 to -0.63)
Saint Kitts and Nevis	2.42 (1.96 to 2.92)	1.81 (0.86 to 2.78)	4.36 (3.16 to 5.85)	4.41 (3.41 to 5.41)	1.81 (1.49 to 2.14)	0.78 (-0.13 to 1.7)	29.67 (24.36 to 35.38)	0.85 (-0.12 to 1.83)
Saint Lucia	5.24 (4.12 to 6.49)	0.25 (-0.68 to 1.19)	9.58 (6.8 to 13.24)	2.39 (1.76 to 3.03)	4 (3.22 to 4.82)	-0.5 (-1.49 to 0.5)	60.91 (48.76 to 73.89)	-0.41 (-1.34 to 0.53)
Saint Vincent and the Grenadines	5.55 (4.44 to 6.75)	1.24 (0.22 to 2.27)	7.86 (5.68 to 10.59)	2.2 (0.07 to 4.36)	4.6 (3.72 to 5.5)	0.7 (-1.15 to 2.59)	68.84 (56.25 to 82.38)	0.8 (-0.14 to 1.76)
Samoa	8.17 (3.77 to 17.49)	-0.25 (-0.3 to -0.2)	5.32 (2.48 to 11.5)	-0.13 (-0.18 to -0.07)	8.66 (4.05 to 18.48)	-0.31 (-0.35 to -0.27)	145.41 (67.98 to 311.24)	-0.43 (-0.48 to -0.38)
San Marino	42.11 (21.72 to 70.33)	-0.45 (-1 to 0.11)	315.28 (162.38 to 525.61)	-0.13 (-0.65 to 0.4)	8.14 (4.44 to 12.78)	-1.65 (-2.15 to -1.14)	152.13 (82.04 to 242.7)	-1.46 (-1.91 to -0.99)
Sao Tome and Principe	0.22 (0.08 to 0.35)	1.12 (1.04 to 1.2)	0.28 (0.11 to 0.48)	2.94 (2.69 to 3.2)	0.19 (0.07 to 0.31)	0.55 (0.48 to 0.62)	3.02 (1.23 to 4.82)	0.55 (0.47 to 0.63)
Saudi Arabia	1.98 (0.96 to 4.22)	3.84 (3.64 to 4.03)	6.69 (3.21 to 14.14)	7.58 (7.37 to 7.79)	0.32 (0.15 to 0.68)	0.08 (0 to 0.15)	5.89 (2.92 to 12.06)	0.33 (0.27 to 0.4)
Senegal	1.62 (0.69 to 2.63)	1.29	1.55 (0.64 to 2.67)	2.14	1.55 (0.65 to 2.53)	1.02	25.71 (11.03 to 42.01)	0.89

© 2025 Wang R, et al. *JAMA Dermatol*.

		(1.2 to 1.38)		(2.07 to 2.22)		(0.93 to 1.11)		(0.78 to 1)
Serbia	29.54 (17.56 to 43.11)	1.77 (1.48 to 2.06)	137.96 (78.22 to 209.22)	5.2 (4.88 to 5.52)	12.31 (7.43 to 17.68)	-0.2 (-0.44 to 0.04)	217.54 (132.24 to 312.7)	0.07 (-0.07 to 0.21)
Seychelles	3.88 (2.06 to 5.67)	1.89 (1.16 to 2.62)	2.87 (1.53 to 4.25)	2.55 (1.64 to 3.46)	3.93 (2.08 to 5.79)	2.04 (1.2 to 2.88)	62.32 (33.54 to 91.51)	1.59 (0.83 to 2.34)
Sierra Leone	1.43 (0.57 to 2.31)	1.1 (0.98 to 1.22)	1.16 (0.46 to 1.96)	1.72 (1.58 to 1.87)	1.4 (0.56 to 2.26)	0.86 (0.74 to 0.97)	23.32 (9.38 to 37.82)	0.78 (0.68 to 0.88)
Singapore	6.91 (5.17 to 8.93)	2.78 (0.82 to 4.78)	50.08 (37.32 to 65.24)	4.65 (1.67 to 7.71)	1.58 (1.25 to 1.92)	-0.05 (-2.34 to 2.3)	28.02 (22.31 to 34.13)	-0.09 (-2.33 to 2.2)
Slovakia	43.78 (21.41 to 67.62)	2.29 (2.14 to 2.44)	275.58 (130.04 to 423.8)	4.11 (3.88 to 4.33)	13.26 (6.7 to 19.94)	0.32 (0.12 to 0.53)	238.24 (119.64 to 358.83)	0.41 (0.21 to 0.62)
Slovenia	87.21 (64.52 to 114.93)	3.81 (3.21 to 4.41)	566.36 (412.6 to 758.99)	5.79 (4.62 to 6.96)	19.65 (15.47 to 23.94)	1.4 (1.25 to 1.54)	345.93 (272.09 to 426.68)	1.42 (1.28 to 1.57)
Solomon Islands	1.74 (0.99 to 2.89)	-0.03 (-0.15 to 0.1)	1.02 (0.59 to 1.71)	0.03 (-0.11 to 0.16)	2.01 (1.16 to 3.36)	-0.03 (-0.19 to 0.12)	32.16 (18.66 to 53.62)	-0.1 (-0.3 to 0.09)
Somalia	4.25 (2.66 to 6.47)	-0.03 (-0.09 to 0.04)	2.8 (1.75 to 4.26)	0.14 (0.07 to 0.21)	4.48 (2.79 to 6.82)	-0.08 (-0.16 to 0)	81.89 (51.37 to 123.62)	-0.08 (-0.16 to -0.01)
South Africa	9.65 (4.61 to 12.48)	1.29	11.39 (5.38 to 15.02)	2.6	8.02 (3.83 to 10.34)	0.79	130.5 (64.14 to 167.49)	0.83

© 2025 Wang R, et al. *JAMA Dermatol*.

		(1 to 1.58)		(2.25 to 2.95)		(0.52 to 1.06)		(0.53 to 1.14)
South Sudan	4.4 (2.62 to 6.65)	0.17 (0.07 to 0.28)	3.28 (1.89 to 5)	0.55 (0.4 to 0.69)	4.36 (2.59 to 6.61)	-0.01 (-0.11 to 0.09)	78.2 (46.1 to 118.55)	-0.03 (-0.1 to 0.04)
Spain	36.69 (27.56 to 47.23)	2.58 (2.06 to 3.1)	271.58 (204.52 to 351.38)	3.38 (2.8 to 3.96)	7.06 (5.64 to 8.5)	0.72 (0.51 to 0.94)	130.3 (104.68 to 157.33)	0.8 (0.56 to 1.04)
Sri Lanka	1.23 (0.61 to 1.95)	1.38 (1.03 to 1.73)	1.17 (0.56 to 1.92)	2.6 (2.29 to 2.9)	1.11 (0.55 to 1.74)	0.79 (0.47 to 1.11)	18.08 (8.88 to 28.42)	0.78 (0.44 to 1.12)
Sudan	4.05 (1.3 to 7.25)	1.15 (1.01 to 1.3)	4.82 (1.57 to 8.95)	3.04 (2.83 to 3.25)	1.61 (0.5 to 2.84)	-0.71 (-0.73 to -0.68)	25.98 (8.32 to 45.72)	-0.71 (-0.74 to -0.68)
Suriname	2.33 (1.32 to 3.64)	0.1 (-0.32 to 0.52)	2.73 (1.48 to 4.34)	1.32 (0.79 to 1.87)	2.02 (1.13 to 3.17)	-0.31 (-0.69 to 0.07)	33.64 (18.9 to 52.3)	-0.41 (-0.77 to -0.04)
Sweden	80.09 (61.61 to 100.83)	2.29 (1.64 to 2.95)	574.11 (441.79 to 723.6)	2.95 (2.4 to 3.5)	17.21 (14 to 20.68)	0.75 (0.38 to 1.11)	309.68 (253.31 to 374.28)	0.62 (0.05 to 1.2)
Switzerland	64.43 (47.19 to 84.2)	2 (1.51 to 2.51)	492.6 (361.72 to 644.92)	2.51 (1.81 to 3.22)	11.38 (8.88 to 13.99)	0.32 (-0.17 to 0.81)	208.64 (162.77 to 256.65)	0.3 (-0.24 to 0.84)
Syrian Arab Republic	5.67 (2.93 to 8.72)	3.71 (3.29 to 4.13)	16.88 (8.61 to 27.18)	7.6 (6.61 to 8.6)	1.15 (0.6 to 1.65)	0.58 (0.21 to 0.94)	18.63 (9.89 to 27.23)	0.7 (0.31 to 1.09)
Taiwan (Province of China)	3.12 (2.47 to 3.8)	0.93 (0.24 to	4.28 (3.11 to 5.71)	2.03 (1.16 to 2.9)	2.47 (1.97 to 2.98)	0.58 (-0.2 to 1.36)	39.59 (31.64 to 47.71)	0.26 (-0.49 to 1.02)

© 2025 Wang R, et al. *JAMA Dermatol*.

		1.63)						
Tajikistan	3.68 (2.18 to 5.37)	-1.08 (-1.56 to -0.6)	4.34 (2.54 to 6.54)	-0.27 (-0.58 to 0.05)	3.23 (1.89 to 4.71)	-1.45 (-2.06 to -0.83)	51.88 (31.2 to 75)	-1.27 (-1.74 to -0.8)
Thailand	0.96 (0.47 to 2.3)	0.08 (-0.11 to 0.26)	0.96 (0.46 to 2.28)	1.31 (1.06 to 1.57)	0.84 (0.42 to 1.98)	-0.48 (-0.66 to -0.3)	13.8 (6.79 to 32.43)	-0.54 (-0.71 to -0.36)
Timor-Leste	1.09 (0.65 to 1.63)	0.84 (0.62 to 1.05)	0.66 (0.39 to 0.99)	0.91 (0.69 to 1.14)	1.23 (0.72 to 1.86)	0.82 (0.69 to 0.95)	20 (11.81 to 30.07)	0.72 (0.64 to 0.8)
Togo	1.75 (0.64 to 2.9)	1.54 (1.44 to 1.65)	1.55 (0.56 to 2.68)	2.3 (2.18 to 2.42)	1.67 (0.6 to 2.79)	1.31 (1.21 to 1.4)	27.76 (10.22 to 46.22)	1.18 (1.09 to 1.27)
Tokelau	1.88 (1.05 to 3.21)	0.04 (-0.24 to 0.31)	1.23 (0.69 to 2.1)	0.31 (0.12 to 0.51)	2.02 (1.14 to 3.45)	-0.15 (-0.29 to 0)	31.7 (17.98 to 54.5)	-0.27 (-0.48 to -0.05)
Tonga	2.03 (1.1 to 3.59)	0.21 (0.03 to 0.39)	1.29 (0.7 to 2.25)	0.27 (0.05 to 0.48)	2.22 (1.2 to 3.94)	0.2 (0.04 to 0.37)	34.47 (18.95 to 61.7)	0.1 (-0.07 to 0.26)
Trinidad and Tobago	2.09 (1.56 to 2.68)	0.36 (-0.09 to 0.82)	3.98 (2.73 to 5.63)	2.85 (2.24 to 3.46)	1.51 (1.15 to 1.89)	-0.55 (-1.04 to -0.06)	24.93 (19.02 to 31.4)	-0.42 (-0.91 to 0.06)
Tunisia	5 (2.65 to 8.17)	2.83 (2.7 to 2.95)	19.61 (9.75 to 32.3)	6.16 (5.79 to 6.52)	0.78 (0.44 to 1.27)	0 (-0.14 to 0.13)	14.24 (7.87 to 22.97)	0.22 (0.11 to 0.34)
Turkmenistan	4.7 (3.57 to 6.08)	-0.66 (-1.79 to	8.38 (5.94 to 11.7)	0.87 (-0.21 to 1.97)	3.52 (2.74 to 4.5)	-1.31 (-2.4 to -0.21)	59.29 (46.07 to 75.89)	-1.31 (-2.36 to

© 2025 Wang R, et al. *JAMA Dermatol*.

		0.49)						-0.24)
Tuvalu	1.78 (1.07 to 2.87)	0.35 (0.31 to 0.38)	1.09 (0.65 to 1.74)	0.49 (0.45 to 0.53)	2.03 (1.21 to 3.3)	0.25 (0.22 to 0.29)	31.88 (19.26 to 51.51)	0.15 (0.11 to 0.2)
Turkey	28.65 (13.27 to 44.46)	3.52 (3.24 to 3.8)	117.85 (55.29 to 188.51)	8.73 (8.34 to 9.13)	4.61 (2.18 to 6.85)	-0.29 (-0.58 to 0)	78.92 (37.75 to 117.44)	-0.1 (-0.43 to 0.23)
Uganda	10.12 (5.8 to 15.39)	1.16 (1.04 to 1.28)	8.3 (4.69 to 12.85)	1.79 (1.65 to 1.94)	9.61 (5.56 to 14.55)	0.86 (0.76 to 0.97)	169.56 (96.68 to 256.67)	0.81 (0.71 to 0.91)
Ukraine	16.75 (11.83 to 22.58)	2.01 (1.54 to 2.48)	70.83 (47.45 to 101.52)	2.97 (2.46 to 3.48)	7.36 (5.39 to 9.6)	1.21 (0.82 to 1.59)	139.38 (101.2 to 182.8)	1.24 (0.55 to 1.94)
United Arab Emirates	35.53 (20.73 to 56.09)	2.71 (0.1 to 5.39)	96.9 (52.89 to 163.12)	5.32 (3.36 to 7.32)	7.01 (4.26 to 10.41)	0.27 (-2.42 to 3.04)	120.74 (74.49 to 179.24)	0.12 (-2.19 to 2.49)
United Kingdom	66.25 (59.05 to 72.15)	3.12 (2.45 to 3.8)	457.17 (409.95 to 498.01)	4.12 (3.49 to 4.74)	14.09 (12.53 to 14.91)	1.41 (1.06 to 1.75)	248.72 (225.67 to 265.17)	1.37 (1 to 1.73)
United Republic of Tanzania	4.89 (2.74 to 7.6)	-0.06 (-0.14 to 0.02)	4.31 (2.36 to 6.94)	0.68 (0.53 to 0.84)	4.62 (2.56 to 7.17)	-0.33 (-0.41 to -0.25)	81.87 (44.82 to 127.83)	-0.37 (-0.46 to -0.28)
United States of America	89.52 (79.41 to 96.14)	1.1 (0.76 to 1.44)	641.68 (574.29 to 686.34)	1.52 (1.15 to 1.89)	12.19 (10.74 to 12.99)	0.12 (-0.13 to 0.37)	232.8 (209.18 to 251.76)	0.04 (-0.2 to 0.29)
United States Virgin Islands	7.05 (4.35 to 11.01)	-1.19 (-1.62 to -0.75)	14.67 (8.32 to 24.13)	0.44 (-0.69 to 1.59)	4.86 (3.04 to 7.49)	-1.74 (-2.13 to -1.36)	74.6 (46.63 to 115.45)	-1.94 (-2.39 to -1.48)
Uruguay	19.77 (15.43 to 24.94)	2.54	76.81 (55.31 to 102.94)	5.06	9.98 (8.01 to 11.99)	1.62	169.28 (136.97 to	1.62

© 2025 Wang R, et al. *JAMA Dermatol*.

		(2.19 to 2.89)		(4.7 to 5.42)		(0.4 to 2.86)	204.03)	(0.49 to 2.75)
Uzbekistan	3.44 (2.67 to 4.33)	1.41 (0.68 to 2.14)	5.88 (4.21 to 8.05)	2.8 (1.98 to 3.63)	2.62 (2.05 to 3.28)	0.93 (0.34 to 1.53)	42.83 (33.53 to 53.66)	0.92 (0.31 to 1.54)
Vanuatu	2.12 (1.24 to 3.37)	0.11 (0.02 to 0.2)	1.24 (0.73 to 1.98)	0.1 (-0.02 to 0.21)	2.45 (1.43 to 3.93)	0.14 (0.05 to 0.23)	39.37 (23.1 to 63.01)	0.09 (-0.04 to 0.23)
Venezuela (Bolivarian Republic of)	5.83 (4.37 to 7.55)	2.26 (2.02 to 2.49)	12.24 (8.22 to 17.07)	4.52 (3.46 to 5.6)	4.17 (3.19 to 5.3)	1.27 (0.81 to 1.73)	68.55 (52.21 to 87.36)	1.17 (0.7 to 1.65)
Viet Nam	1.47 (0.87 to 2.23)	1.73 (1.62 to 1.83)	1.31 (0.74 to 2.02)	2.82 (2.69 to 2.94)	1.38 (0.83 to 2.09)	1.28 (1.2 to 1.36)	23.22 (13.79 to 34.65)	1.21 (1.13 to 1.29)
Yemen	3.92 (1.2 to 7.22)	1.4 (1.11 to 1.69)	3.94 (1.23 to 7.39)	2.62 (2.34 to 2.89)	1.9 (0.56 to 3.45)	0.26 (0.18 to 0.34)	30.44 (9.28 to 55.41)	0.18 (0.11 to 0.26)
Zambia	7.2 (3.08 to 12.11)	1.44 (1.24 to 1.65)	6.24 (2.52 to 10.78)	2.33 (2.11 to 2.54)	6.8 (2.97 to 11.38)	1.08 (0.86 to 1.3)	124.96 (52.43 to 213.58)	1.15 (0.94 to 1.37)
Zimbabwe	11.47 (6.79 to 17.19)	0.89 (0.5 to 1.28)	8.72 (4.95 to 13.37)	0.82 (0.39 to 1.24)	11.37 (6.66 to 16.96)	0.85 (0.63 to 1.08)	197.73 (113.82 to 293.46)	0.94 (0.71 to 1.17)

Abbreviations: UI, uncertainty interval; ASR, age-standerised rate per 100,000; CI, confidence interval; DALYs, disability-adjusted life-year.

© 2025 Wang R, et al. *JAMA Dermatol*.

eTable 5. Burden of squamous cell carcinoma in 204 countries and territories in 2021 and the average annual percent change (AAPC) from 1990 to 2021.

Country	Incidence		Prevalence		Deaths		DALYs	
	ASR (95% UI)	AAPC (95% CI)	ASR (95% UI)	AAPC (95% CI)	ASR (95% UI)	AAPC (95% CI)	ASR (95% UI)	AAPC (95% CI)
Afghanistan	1.93 (1.23 to 2.9)	-0.36 (-0.39 to -0.34)	3.56 (2.18 to 5.38)	-0.51 (-0.55 to -0.47)	0.11 (0.03 to 0.8)	2.9 (2.77 to 3.03)	1.83 (0.56 to 11.99)	2.4 (2.3 to 2.5)
Albania	24.92 (16.45 to 36.35)	0.25 (0.21 to 0.28)	45.48 (30.2 to 65.98)	0.22 (0.21 to 0.23)	12.55 (8.09 to 18.86)	-0.71 (-0.96 to -0.46)	156.1 (101.05 to 235.91)	-0.95 (-1.21 to -0.7)
Algeria	7.33 (4.83 to 10.79)	-0.13 (-0.2 to -0.07)	9.72 (6.31 to 14.03)	-0.13 (-0.17 to -0.1)	0.12 (0.02 to 0.96)	3.04 (2.73 to 3.35)	1.97 (0.63 to 12.71)	1.91 (1.79 to 2.03)
American Samoa	0.02 (0 to 0.04)	0 (0 to 0.01)	0.05 (0.01 to 0.12)	0 (0 to 0.01)	8.27 (4.94 to 12.68)	1.33 (0.58 to 2.09)	104.89 (62.37 to 160.85)	1.41 (0.66 to 2.16)
Andorra	52.78 (34.78 to 74.56)	0.03 (-0.03 to 0.08)	63.94 (42.77 to 93.49)	-0.07 (-0.13 to -0.01)	4.86 (2.83 to 7.91)	-1 (-1.59 to -0.4)	66.22 (39.59 to 106.56)	-1.06 (-1.65 to -0.46)
Angola	0.25 (0.14 to 0.4)	-0.15 (-0.16 to -0.14)	0.43 (0.22 to 0.73)	-0.38 (-0.39 to -0.37)	4.31 (1.25 to 8.27)	1.44 (1.31 to 1.57)	61.63 (17.75 to 116.75)	1.38 (1.24 to 1.52)
Antigua and Barbuda	0.3 (0.17 to 0.46)	0.19 (0.17 to	0.55 (0.29 to 0.89)	0.04 (0.02 to	9.3 (7.92 to 10.84)	8.34 (6.23 to	126.18 (107.49 to 147.38)	8.03 (5.91 to

© 2025 Wang R, et al. *JAMA Dermatol*.

		0.21)		0.07)		10.49)		10.2)
Argentina	26.38 (17.59 to 38.6)	0.19 (0.14 to 0.23)	45.14 (29.63 to 65.67)	0.03 (-0.03 to 0.09)	7.3 (6.2 to 8.35)	0.24 (-0.03 to 0.52)	103.99 (89.37 to 118.58)	0.15 (-0.24 to 0.54)
Armenia	0.27 (0.16 to 0.42)	0.08 (0.07 to 0.1)	0.53 (0.28 to 0.88)	-0.03 (-0.09 to 0.02)	11.1 (9.02 to 13.19)	3.83 (2.84 to 4.83)	154.56 (126.05 to 183.63)	3.76 (2.78 to 4.75)
Australia	431.61 (284.11 to 618.18)	-0.16 (-0.22 to -0.1)	483.88 (319.66 to 695.69)	-0.19 (-0.25 to -0.13)	14.55 (11.95 to 16.73)	0.54 (0.05 to 1.02)	217.14 (182.17 to 249.66)	0.23 (-0.25 to 0.71)
Austria	31.27 (20.73 to 45.46)	0.73 (0.54 to 0.91)	46.02 (29.91 to 68)	0.55 (0.48 to 0.62)	6.72 (5.42 to 7.81)	0.44 (0.19 to 0.69)	89.28 (73.71 to 103.26)	0.19 (-0.62 to 1.02)
Azerbaijan	0.26 (0.16 to 0.42)	0.24 (0.22 to 0.26)	0.56 (0.3 to 0.9)	0.23 (0.18 to 0.28)	4.39 (2.54 to 7.22)	0.32 (-0.12 to 0.76)	66.25 (38.5 to 108.65)	0.52 (0.12 to 0.92)
Bahamas	0.29 (0.17 to 0.45)	0.14 (0.13 to 0.16)	0.52 (0.27 to 0.86)	-0.01 (-0.04 to 0.03)	5.68 (4.52 to 7.06)	1.13 (-0.22 to 2.49)	82.77 (65.29 to 103.73)	0.98 (-0.3 to 2.27)
Bahrain	0.1 (0.04 to 0.19)	-0.51 (-0.73 to -0.28)	0.22 (0.09 to 0.42)	-0.67 (-0.9 to -0.45)	3.77 (2.48 to 5.16)	1.45 (0.68 to 2.22)	48.78 (32.17 to 67.04)	1.04 (0.51 to 1.58)
Bangladesh	0.02 (0 to 0.04)	-0.06 (-0.07 to -0.05)	0.03 (0.01 to 0.08)	-0.28 (-0.3 to -0.25)	3.63 (1.85 to 6.5)	0.83 (0.35 to 1.32)	49.34 (24.76 to 89.27)	0.62 (0.2 to 1.03)
Barbados	0.29 (0.17 to 0.46)	0.21	0.54 (0.28 to 0.9)	0.07	3.99 (3.15 to 4.86)	0.23	50.18 (39.29 to 61.56)	0.24

© 2025 Wang R, et al. *JAMA Dermatol*.

		(0.2 to 0.22)		(0.05 to 0.09)		(0 to 0.45)		(0.04 to 0.44)
Belarus	20.23 (13.4 to 29.12)	0.35 (0.27 to 0.43)	35.29 (23.35 to 50.25)	0.21 (0.07 to 0.34)	6.57 (5.33 to 7.93)	-0.08 (-0.53 to 0.37)	94.83 (77.5 to 114.76)	-0.1 (-0.5 to 0.3)
Belgium	36.48 (24.13 to 52.73)	0.47 (0.4 to 0.55)	47.87 (31.51 to 70.47)	0.35 (0.24 to 0.47)	5.08 (4.11 to 5.89)	-0.16 (-0.73 to 0.4)	72.12 (59.82 to 82.97)	-0.13 (-0.54 to 0.28)
Belize	0.32 (0.19 to 0.49)	0.15 (0.14 to 0.16)	0.59 (0.31 to 0.96)	-0.02 (-0.06 to 0.03)	7.93 (6.51 to 9.43)	1.1 (0.18 to 2.02)	110.4 (91.01 to 131.25)	0.97 (0.16 to 1.79)
Benin	0.04 (0.01 to 0.07)	-0.11 (-0.15 to -0.07)	0.07 (0.02 to 0.15)	-0.35 (-0.37 to -0.32)	1.08 (0.35 to 1.95)	1.39 (1.16 to 1.62)	15.6 (4.8 to 28.22)	1.42 (1.2 to 1.65)
Bermuda	0.01 (0 to 0.03)	-1.12 (-1.13 to -1.11)	0.02 (0 to 0.06)	-1.18 (-1.2 to -1.16)	8.08 (6.46 to 9.96)	3.45 (2.26 to 4.66)	107.46 (85.69 to 133.27)	3.35 (2.15 to 4.56)
Bhutan	0.02 (0 to 0.04)	0.09 (0.06 to 0.11)	0.03 (0.01 to 0.07)	-0.12 (-0.15 to -0.1)	3.58 (2.15 to 5.79)	1.17 (1.1 to 1.24)	48.93 (29.33 to 78.75)	0.98 (0.91 to 1.06)
Bolivia (Plurinational State of)	1.24 (0.82 to 1.79)	0.06 (0.05 to 0.07)	2.26 (1.37 to 3.39)	-0.1 (-0.12 to -0.07)	11.88 (6.84 to 18.88)	1.52 (1.45 to 1.59)	161.45 (91.35 to 260.18)	1.45 (1.38 to 1.53)
Bosnia and Herzegovina	24.12 (15.89 to 34.98)	0.16 (0.14 to 0.17)	41.42 (27.03 to 60.05)	0.06 (0.02 to 0.1)	5.34 (3.78 to 7.19)	0.17 (-0.23 to 0.57)	73.96 (52.96 to 99.09)	0.06 (-0.35 to 0.47)

© 2025 Wang R, et al. *JAMA Dermatol*.

Botswana	18.89 (12.79 to 26.92)	0.02 (-0.01 to 0.04)	27.68 (18.47 to 39.63)	-0.17 (-0.19 to -0.14)	3.91 (2.36 to 6.79)	0.39 (-0.23 to 1.02)	59.02 (36.19 to 103.04)	0.41 (-0.12 to 0.95)
Brazil	20.35 (14.75 to 27.63)	-0.13 (-0.24 to -0.02)	22.21 (16.14 to 29.65)	-0.16 (-0.28 to -0.05)	11.57 (9.81 to 12.66)	1.35 (0.9 to 1.79)	161.09 (140.93 to 174.3)	1.16 (0.64 to 1.68)
Brunei Darussalam	6.41 (4.25 to 9.37)	-0.05 (-0.09 to -0.02)	11.65 (7.45 to 17.13)	-0.2 (-0.23 to -0.17)	4.83 (3.15 to 7.23)	-0.8 (-1.17 to -0.44)	62.73 (41.49 to 93.37)	-1.03 (-1.36 to -0.69)
Bulgaria	30.99 (24.53 to 37.59)	0.47 (0.32 to 0.63)	49.04 (34.82 to 66.48)	0.12 (0 to 0.24)	9.05 (7.68 to 10.43)	-1.29 (-3.02 to 0.48)	119.6 (101.56 to 138.44)	-1.12 (-2.8 to 0.59)
Burkina Faso	0.04 (0.01 to 0.07)	-0.05 (-0.06 to -0.04)	0.07 (0.02 to 0.15)	-0.29 (-0.34 to -0.24)	1.12 (0.34 to 2.03)	1.43 (1.21 to 1.65)	16.09 (4.62 to 29.53)	1.51 (1.31 to 1.71)
Burundi	0.25 (0.14 to 0.4)	0.18 (0.17 to 0.2)	0.45 (0.23 to 0.77)	0.03 (0.01 to 0.05)	3.83 (0.86 to 8.01)	1.05 (0.92 to 1.19)	54.54 (12.27 to 112.87)	0.98 (0.82 to 1.14)
Cabo Verde	0.03 (0.01 to 0.07)	-0.16 (-0.19 to -0.14)	0.07 (0.02 to 0.14)	-0.43 (-0.46 to -0.4)	0.51 (0.28 to 0.87)	-0.25 (-0.77 to 0.28)	7.09 (3.91 to 12.22)	-0.18 (-0.78 to 0.42)
Cambodia	0.37 (0.22 to 0.56)	-0.18 (-0.2 to -0.17)	0.65 (0.36 to 1.04)	-0.35 (-0.38 to -0.33)	5.66 (3.46 to 8.65)	1.03 (0.98 to 1.08)	87.86 (53.59 to 134.3)	0.89 (0.84 to 0.94)
Cameroon	0.04 (0.01 to 0.07)	-0.06 (-0.09 to	0.07 (0.02 to 0.15)	-0.27 (-0.31 to	1.29 (0.41 to 2.24)	1.45 (1.34 to	18.83 (5.68 to 33.14)	1.56 (1.44 to

© 2025 Wang R, et al. *JAMA Dermatol*.

		-0.03)		-0.23)		1.57)		1.67)
Canada	64.31 (41.82 to 93.97)	1.23 (1.17 to 1.29)	111.36 (70.52 to 162.3)	0.96 (0.9 to 1.01)	7.1 (5.89 to 8.14)	0.99 (0.5 to 1.49)	99.22 (84.27 to 113.18)	0.71 (0.28 to 1.13)
Central African Republic	0.24 (0.13 to 0.38)	-0.13 (-0.14 to -0.12)	0.41 (0.21 to 0.7)	-0.33 (-0.35 to -0.32)	3.64 (1.13 to 6.77)	0.75 (0.67 to 0.84)	53.24 (16.32 to 97.05)	0.75 (0.69 to 0.81)
Chad	0.04 (0.01 to 0.08)	0.14 (0.11 to 0.16)	0.08 (0.03 to 0.16)	-0.02 (-0.06 to 0.01)	1.15 (0.34 to 2.23)	2.28 (2.14 to 2.42)	17.08 (4.81 to 33.49)	2.39 (2.28 to 2.51)
Chile	22.42 (14.67 to 32.64)	0.1 (0.07 to 0.13)	41.19 (26.72 to 60.24)	-0.07 (-0.09 to -0.05)	9.91 (8.17 to 11.33)	2.1 (0.96 to 3.25)	127.35 (106.76 to 145.04)	2.05 (0.77 to 3.33)
China	44.34 (29.5 to 64.76)	6.02 (5.67 to 6.37)	97.17 (64.67 to 138.07)	7.04 (6.74 to 7.35)	7.58 (5.65 to 9.4)	0.63 (0.35 to 0.91)	115.39 (88.26 to 142.52)	0.59 (0.4 to 0.78)
Colombia	7.97 (5.49 to 11.4)	-0.07 (-0.1 to -0.05)	13.49 (8.81 to 19.47)	-0.3 (-0.32 to -0.28)	8.56 (6.82 to 10.31)	-1.15 (-1.53 to -0.76)	118.88 (95.36 to 144.02)	-1.4 (-1.84 to -0.97)
Comoros	0.24 (0.13 to 0.38)	-0.11 (-0.11 to -0.1)	0.42 (0.21 to 0.71)	-0.32 (-0.33 to -0.3)	4.59 (1.39 to 9.07)	1.43 (1.35 to 1.51)	63.95 (19.51 to 126.25)	1.31 (1.2 to 1.43)
Congo	0.26 (0.14 to 0.4)	0.11 (0.09 to 0.12)	0.45 (0.23 to 0.76)	-0.05 (-0.06 to -0.03)	4.89 (1.61 to 8.72)	1.02 (0.93 to 1.11)	69.1 (22.36 to 121.26)	0.99 (0.92 to 1.06)
Cook Islands	0.02 (0 to 0.04)	-0.02	0.05 (0.01 to 0.12)	-0.02	2.73 (1.66 to 4.39)	-0.46	34.48 (21.07 to 55.45)	-0.54

© 2025 Wang R, et al. *JAMA Dermatol*.

		(-0.02 to -0.01)		(-0.02 to -0.01)		(-1 to 0.09)		(-1.1 to 0.02)
Costa Rica	8.35 (5.78 to 12.05)	-3.37 (-3.51 to -3.23)	15.53 (10.28 to 22.38)	-2.41 (-2.55 to -2.27)	8.53 (6.9 to 10.12)	-0.84 (-1.44 to -0.24)	117.03 (96.01 to 138.74)	-0.85 (-1.37 to -0.34)
Croatia	16.2 (10.91 to 23.33)	0.19 (0.16 to 0.22)	29.35 (19.52 to 41.87)	0.15 (0.13 to 0.18)	9.13 (7.57 to 10.64)	-2.42 (-3.5 to -1.33)	111.99 (93.47 to 130.84)	-2.52 (-3.59 to -1.45)
Cuba	4.48 (3.01 to 6.35)	-2.56 (-3.23 to -1.88)	5.04 (3.36 to 7.09)	-2.36 (-2.98 to -1.73)	18.9 (15.67 to 22.1)	0.59 (-0.12 to 1.3)	257.84 (213.71 to 302.45)	0.68 (0 to 1.36)
Cyprus	19.92 (13.08 to 29.44)	0.07 (0.04 to 0.11)	34.23 (21.89 to 50.92)	-0.01 (-0.03 to 0.01)	10.93 (6.74 to 16.49)	-2.21 (-2.94 to -1.47)	132.79 (83.1 to 201.43)	-2.26 (-2.96 to -1.55)
Czechia	54.19 (36.27 to 78.89)	0.42 (0.12 to 0.72)	66.85 (45.2 to 96.13)	0.33 (0.17 to 0.5)	6 (5.01 to 6.93)	-2.52 (-3.63 to -1.39)	82.09 (69.2 to 95.04)	-2.43 (-3.44 to -1.41)
Côte d'Ivoire	0.01 (0 to 0.03)	-0.61 (-0.65 to -0.58)	0.02 (0 to 0.06)	-0.95 (-1.06 to -0.83)	1.24 (0.39 to 2.22)	1.59 (1.41 to 1.77)	18.04 (5.27 to 32.71)	1.68 (1.49 to 1.88)
Democratic People's Republic of Korea	1.58 (1 to 2.36)	0.26 (0.2 to 0.33)	2.81 (1.7 to 4.18)	0.11 (0.05 to 0.16)	5.67 (3.41 to 9.23)	0.7 (0.65 to 0.76)	89.06 (55.04 to 141.31)	0.68 (0.62 to 0.74)
Democratic Republic of the Congo	0.25 (0.14 to 0.39)	-0.21 (-0.23 to -0.19)	0.42 (0.22 to 0.72)	-0.48 (-0.51 to -0.46)	4.01 (1.18 to 7.87)	1.18 (1.12 to 1.24)	57.48 (16.9 to 111.14)	1.18 (1.11 to 1.24)

© 2025 Wang R, et al. *JAMA Dermatol*.

Denmark	54.61 (36.13 to 78.48)	-0.17 (-0.24 to -0.1)	66.04 (43.59 to 95.33)	-0.21 (-0.25 to -0.16)	6.14 (5.03 to 7.13)	1.34 (0.37 to 2.32)	82.36 (69.03 to 95.11)	0.96 (-0.14 to 2.07)
Djibouti	0.25 (0.14 to 0.4)	0.12 (0.1 to 0.15)	0.46 (0.23 to 0.77)	-0.04 (-0.06 to -0.01)	4.77 (1.27 to 10.19)	1.56 (1.46 to 1.67)	67.62 (17.83 to 144.48)	1.55 (1.49 to 1.62)
Dominica	0.29 (0.17 to 0.45)	0.28 (0.25 to 0.31)	0.53 (0.28 to 0.87)	0.11 (0.09 to 0.12)	5.14 (3.32 to 7.61)	0.38 (0.33 to 0.43)	73.27 (47.17 to 109.55)	0.45 (0.39 to 0.52)
Dominican Republic	0.3 (0.17 to 0.46)	-0.07 (-0.1 to -0.05)	0.56 (0.29 to 0.92)	-0.28 (-0.31 to -0.24)	11.68 (7.16 to 17.76)	2.43 (1.66 to 3.2)	161.09 (98.43 to 245.34)	2.54 (1.68 to 3.41)
Ecuador	2.42 (1.63 to 3.47)	-2.71 (-2.93 to -2.49)	4.28 (2.73 to 6.27)	-1.98 (-2.17 to -1.79)	15.94 (13 to 19.38)	3.33 (1.8 to 4.89)	200.51 (161.6 to 247.1)	3.12 (1.55 to 4.72)
Egypt	3.38 (2.15 to 5.01)	-0.12 (-0.21 to -0.04)	5.47 (3.4 to 8.22)	-0.14 (-0.2 to -0.08)	2.42 (1.42 to 3.51)	6.41 (5.5 to 7.32)	34.74 (20.17 to 49.98)	6.44 (5.68 to 7.2)
El Salvador	7.9 (5.44 to 11.27)	-0.09 (-0.1 to -0.07)	12.91 (8.42 to 18.7)	-0.31 (-0.35 to -0.26)	4.63 (3.21 to 6.44)	0.75 (0.23 to 1.27)	65.17 (45.64 to 90.75)	0.73 (0.54 to 0.93)
Equatorial Guinea	0.25 (0.14 to 0.39)	-0.04 (-0.05 to -0.02)	0.43 (0.22 to 0.71)	-0.2 (-0.22 to -0.18)	4.4 (1.37 to 7.98)	1.44 (1.3 to 1.58)	61.52 (18.94 to 111.37)	1.28 (1.14 to 1.42)
Eritrea	0.22 (0.13 to 0.36)	0.05 (0.04 to	0.38 (0.2 to 0.66)	-0.17 (-0.19 to	4.46 (1.42 to 8.55)	1.74 (1.68 to 1.8)	62.27 (20.06 to 117)	1.62 (1.54 to

© 2025 Wang R, et al. *JAMA Dermatol*.

		0.06)		-0.16)				1.69)
Estonia	20.13 (13.33 to 28.92)	0.01 (-0.03 to 0.05)	34.36 (22.97 to 48.78)	0.04 (0 to 0.08)	8.23 (6.72 to 9.8)	-0.08 (-0.89 to 0.73)	111.69 (92.09 to 133.31)	-0.14 (-0.78 to 0.51)
Eswatini	17.65 (11.99 to 24.93)	-0.08 (-0.1 to -0.07)	25.45 (17.09 to 36.57)	-0.26 (-0.28 to -0.23)	4.15 (2.24 to 7.06)	0.86 (0.68 to 1.03)	65.21 (35.78 to 110.06)	1.05 (0.9 to 1.21)
Ethiopia	0.27 (0.15 to 0.43)	-0.06 (-0.07 to -0.04)	0.45 (0.23 to 0.78)	-0.15 (-0.18 to -0.13)	3.84 (0.96 to 7.17)	0.87 (0.79 to 0.95)	54.04 (13.61 to 100.2)	0.68 (0.62 to 0.73)
Fiji	0.02 (0 to 0.04)	-0.05 (-0.05 to -0.04)	0.05 (0.01 to 0.12)	-0.1 (-0.12 to -0.08)	3.69 (2.35 to 5.77)	0.06 (-0.31 to 0.43)	46.4 (29.41 to 72.55)	-0.04 (-0.36 to 0.28)
Finland	48.04 (31.69 to 69.48)	0.41 (0.39 to 0.42)	60.48 (39.35 to 88.12)	0.44 (0.42 to 0.46)	4.11 (3.27 to 4.81)	-0.25 (-0.72 to 0.23)	56.29 (46.36 to 65.29)	-0.29 (-0.7 to 0.13)
France	52.72 (35.53 to 75.59)	-0.04 (-0.17 to 0.09)	61.64 (41.24 to 88.31)	-0.04 (-0.21 to 0.12)	5.9 (4.77 to 6.86)	0.28 (-0.39 to 0.96)	77.49 (64.09 to 89.49)	0.31 (-0.71 to 1.34)
Gabon	0.26 (0.14 to 0.41)	0.04 (0.03 to 0.05)	0.44 (0.23 to 0.75)	-0.15 (-0.17 to -0.13)	4.84 (1.58 to 8.45)	1.02 (0.98 to 1.06)	68.48 (21.33 to 118.56)	1 (0.88 to 1.12)
Gambia	0.95 (0.54 to 1.5)	-0.03 (-0.04 to -0.01)	1.36 (0.75 to 2.2)	-0.12 (-0.14 to -0.1)	1.3 (0.45 to 2.31)	1.9 (1.68 to 2.13)	19.02 (6.25 to 34.13)	1.98 (1.69 to 2.26)
Georgia	0.25 (0.15 to 0.4)	0.13	0.55 (0.3 to 0.93)	0.04	30.76 (25.06 to	11.91	447.81 (366.45 to	11.7

© 2025 Wang R, et al. *JAMA Dermatol*.

		(0.1 to 0.15)		(0 to 0.09)	36.57)	(9.18 to 14.72)	531.39)	(9.16 to 14.3)
Germany	35.8 (23.71 to 52.61)	1.52 (1.11 to 1.94)	48.69 (32.19 to 71.8)	1.17 (0.93 to 1.41)	4.99 (4.06 to 5.72)	0.75 (0.28 to 1.23)	67.23 (56.39 to 76.51)	0.74 (0.34 to 1.14)
Ghana	0.04 (0.01 to 0.07)	-0.18 (-0.19 to -0.17)	0.07 (0.02 to 0.14)	-0.44 (-0.47 to -0.42)	0.85 (0.5 to 1.37)	0.35 (0.18 to 0.52)	12.43 (7.34 to 20.26)	0.35 (0.26 to 0.44)
Greece	50.64 (33.65 to 73.15)	0.1 (0.09 to 0.12)	60.51 (39.69 to 87.8)	-0.03 (-0.07 to 0.02)	8.04 (6.65 to 9.23)	-0.27 (-0.57 to 0.04)	107.18 (90.2 to 122.49)	-0.18 (-0.46 to 0.09)
Greenland	247.77 (166.82 to 360.48)	0.25 (0.12 to 0.38)	283.2 (190.59 to 409.75)	0.25 (0.13 to 0.37)	1.55 (0.99 to 2.15)	-1.53 (-2.7 to -0.35)	35.23 (24.47 to 47.58)	-1.02 (-1.58 to -0.46)
Grenada	0.28 (0.16 to 0.44)	0.08 (0.06 to 0.1)	0.51 (0.27 to 0.84)	-0.1 (-0.13 to -0.07)	5.76 (4.69 to 6.95)	8.93 (5.97 to 11.98)	96.05 (77.78 to 116.5)	9.33 (6.42 to 12.31)
Guam	0.02 (0 to 0.04)	-0.01 (-0.02 to -0.01)	0.05 (0.01 to 0.12)	-0.01 (-0.02 to -0.01)	1.68 (1.05 to 2.4)	-2.44 (-4.24 to -0.61)	24.51 (15.51 to 34.47)	-1.75 (-3.33 to -0.15)
Guatemala	8.28 (5.75 to 11.55)	-0.04 (-0.04 to -0.03)	14.03 (9.21 to 19.97)	-0.22 (-0.26 to -0.17)	9.13 (7.54 to 10.89)	-1.87 (-2.97 to -0.75)	123.45 (101.69 to 148.42)	-1.63 (-2.57 to -0.68)
Guinea	0.01 (0 to 0.03)	-0.19 (-0.24 to -0.14)	0.02 (0 to 0.07)	-0.39 (-0.43 to -0.36)	1.2 (0.37 to 2.23)	2.16 (2.03 to 2.3)	17.65 (5.21 to 33.04)	2.28 (2.13 to 2.42)

© 2025 Wang R, et al. *JAMA Dermatol*.

Guinea-Bissau	0.04 (0.01 to 0.07)	-0.19 (-0.2 to -0.18)	0.07 (0.02 to 0.15)	-0.43 (-0.45 to -0.41)	1.28 (0.45 to 2.17)	1.47 (1.36 to 1.59)	18.76 (6.31 to 32.02)	1.5 (1.37 to 1.64)
Guyana	0.29 (0.17 to 0.45)	-0.06 (-0.08 to -0.05)	0.52 (0.27 to 0.85)	-0.25 (-0.28 to -0.23)	5.23 (3.9 to 6.78)	8.3 (6.2 to 10.44)	77.43 (57.91 to 100.42)	8.29 (5.86 to 10.77)
Haiti	0.3 (0.17 to 0.46)	0.1 (0.09 to 0.11)	0.54 (0.29 to 0.88)	-0.06 (-0.1 to -0.02)	7.02 (3.77 to 11.56)	1.71 (1.61 to 1.81)	99.74 (54.12 to 163.39)	1.61 (1.5 to 1.71)
Honduras	8.33 (5.78 to 11.69)	-0.04 (-0.05 to -0.02)	13.58 (9.1 to 19.51)	-0.22 (-0.24 to -0.2)	6.86 (4.02 to 11.14)	1.19 (0.98 to 1.4)	97.54 (57.34 to 158.52)	1.18 (0.99 to 1.37)
Hungary	24.71 (16.4 to 35.83)	-0.17 (-0.24 to -0.1)	38.28 (25.23 to 55.6)	-0.3 (-0.35 to -0.24)	7.01 (5.81 to 8.17)	-1.93 (-3.09 to -0.76)	96.9 (80.7 to 112.93)	-1.73 (-2.78 to -0.67)
Iceland	50.28 (33.1 to 72.72)	0.12 (0.1 to 0.14)	63.43 (41.25 to 92.43)	0.13 (0.11 to 0.16)	2.55 (2.01 to 3.01)	0.75 (0.6 to 0.91)	35.53 (28.84 to 41.77)	0.51 (0.38 to 0.64)
India	1.18 (0.73 to 1.76)	0.26 (0.22 to 0.3)	1.49 (0.87 to 2.3)	0.24 (0.2 to 0.28)	3.55 (2.83 to 4.63)	0.75 (0.05 to 1.45)	50.42 (40.41 to 65.87)	0.69 (0.09 to 1.28)
Indonesia	0.47 (0.28 to 0.71)	-0.03 (-0.05 to 0)	0.73 (0.4 to 1.16)	-0.16 (-0.19 to -0.14)	5.23 (3.66 to 6.97)	1.51 (1.46 to 1.56)	79.89 (55.48 to 106.05)	1.35 (1.32 to 1.39)
Iran (Islamic Republic of)	6.5 (4.29 to 9.5)	-0.95 (-1.34 to	8.75 (5.64 to 12.95)	-0.82 (-1.14 to	0.32 (0.01 to 0.47)	1.23 (1.05 to 1.4)	4.73 (0.7 to 6.8)	0.91 (0.63 to 1.2)

© 2025 Wang R, et al. *JAMA Dermatol*.

		-0.56)		-0.49)				
Iraq	3.37 (2.5 to 4.49)	0.67 (0.54 to 0.81)	5.36 (3.61 to 7.64)	0.38 (0.31 to 0.45)	3.66 (2.25 to 5.76)	1.08 (0.72 to 1.44)	51.89 (31.89 to 81.43)	0.94 (0.63 to 1.25)
Ireland	76.33 (50.52 to 110.22)	-0.74 (-0.8 to -0.69)	84.58 (56.91 to 119.58)	-0.69 (-0.75 to -0.64)	9.51 (7.5 to 11.47)	-1.39 (-2.07 to -0.71)	128.89 (103.76 to 154.84)	-1.51 (-2.06 to -0.95)
Israel	52.21 (34.87 to 75.31)	-0.03 (-0.05 to -0.01)	58.05 (38.33 to 83.07)	-0.08 (-0.1 to -0.06)	8.92 (7.02 to 10.41)	0.26 (-0.49 to 1.02)	113.8 (91.91 to 131.95)	0.05 (-0.67 to 0.77)
Italy	51.05 (33.59 to 74.43)	0.35 (0.28 to 0.42)	64.88 (42.8 to 95.51)	0.24 (0.19 to 0.29)	5.9 (4.84 to 6.53)	0.13 (-0.5 to 0.77)	78.75 (67.03 to 86.49)	-0.01 (-0.64 to 0.62)
Jamaica	0.11 (0.06 to 0.18)	-1.46 (-1.56 to -1.35)	0.23 (0.11 to 0.4)	-1.54 (-1.65 to -1.43)	2.8 (2.12 to 3.58)	0.7 (-0.12 to 1.52)	40.21 (30.35 to 51.78)	0.78 (-0.05 to 1.61)
Japan	5.1 (3.24 to 7.63)	1.24 (1.21 to 1.27)	9.84 (6.06 to 14.63)	1.2 (1.18 to 1.23)	2.34 (1.84 to 2.62)	-0.35 (-0.89 to 0.19)	30.64 (25.45 to 33.74)	-0.29 (-0.83 to 0.26)
Jordan	8.5 (5.57 to 12.4)	-0.69 (-0.78 to -0.6)	10.65 (6.92 to 15.87)	-0.55 (-0.63 to -0.46)	3.4 (2.38 to 4.58)	1.82 (1.23 to 2.41)	45.41 (31.87 to 61.31)	1.57 (1.11 to 2.02)
Kazakhstan	0.25 (0.15 to 0.39)	0.15 (0.11 to 0.19)	0.53 (0.28 to 0.86)	0.07 (0.02 to 0.11)	8.9 (7.44 to 10.43)	2.19 (1.65 to 2.73)	125.1 (104.93 to 146.48)	2.07 (1.61 to 2.53)
Kenya	0.69 (0.43 to 1.04)	-0.94	1.04 (0.6 to 1.64)	-0.93	4.23 (1.39 to 6.75)	1.65	59.16 (19.03 to 93.07)	1.67

© 2025 Wang R, et al. *JAMA Dermatol*.

		(-1.02 to -0.85)		(-1.1 to -0.76)		(1.52 to 1.77)		(1.55 to 1.8)
Kiribati	0.02 (0 to 0.04)	-0.04 (-0.05 to -0.03)	0.05 (0.01 to 0.12)	-0.1 (-0.13 to -0.08)	1.36 (0.81 to 2.12)	0.63 (0.57 to 0.7)	16.99 (10.21 to 26.45)	0.54 (0.47 to 0.6)
Kuwait	2.31 (1.47 to 3.4)	0.07 (-0.03 to 0.18)	4.15 (2.52 to 6.23)	-0.04 (-0.1 to 0.03)	2.16 (1.62 to 2.74)	2.11 (-4.56 to 9.25)	28.42 (21.63 to 36.16)	2 (-4.62 to 9.07)
Kyrgyzstan	0.26 (0.15 to 0.41)	-2.76 (-2.87 to -2.65)	0.56 (0.3 to 0.93)	-2.56 (-2.66 to -2.46)	15.13 (12.1 to 18.57)	4.12 (2.56 to 5.71)	213.54 (171.18 to 262.27)	4.02 (2.64 to 5.42)
Lao People's Democratic Republic	0.44 (0.26 to 0.66)	0.09 (0.05 to 0.14)	0.76 (0.41 to 1.22)	-0.07 (-0.13 to -0.02)	4.47 (2.73 to 6.9)	0.64 (0.58 to 0.7)	69.62 (41.93 to 107.3)	0.5 (0.46 to 0.55)
Latvia	16.34 (11 to 23.6)	0.04 (0.01 to 0.07)	31.51 (21.1 to 44.67)	0.03 (0.01 to 0.05)	9.47 (7.87 to 11.07)	0.45 (-0.86 to 1.79)	130.51 (109.37 to 152.9)	0.35 (-0.7 to 1.42)
Lebanon	9.26 (6.08 to 13.4)	0.06 (-0.27 to 0.4)	11.19 (7.31 to 16.49)	0.01 (-0.35 to 0.37)	2.15 (1.36 to 3.41)	0.62 (0.43 to 0.8)	28.89 (18.44 to 46.19)	0.46 (0.23 to 0.69)
Lesotho	17.48 (11.89 to 24.91)	0.03 (0.02 to 0.04)	24.96 (16.54 to 35.77)	-0.14 (-0.17 to -0.11)	4.42 (2.48 to 7.5)	1.6 (1.38 to 1.82)	70.55 (40.12 to 120.22)	1.75 (1.51 to 2)
Liberia	0.04 (0.01 to 0.07)	-0.12 (-0.17 to -0.07)	0.08 (0.03 to 0.16)	-0.37 (-0.43 to -0.31)	1.12 (0.35 to 2.09)	1.52 (1.32 to 1.73)	16.3 (4.83 to 30.69)	1.58 (1.32 to 1.84)

Libya	0.2 (0.1 to 0.34)	-0.13 (-0.27 to 0)	0.45 (0.21 to 0.78)	-0.17 (-0.32 to -0.02)	0.16 (0.03 to 1.27)	4.17 (3.86 to 4.48)	2.31 (0.4 to 18.52)	4.12 (3.84 to 4.4)
Lithuania	21.35 (14.21 to 30.43)	-0.15 (-0.23 to -0.07)	35.65 (23.68 to 51.47)	-0.27 (-0.31 to -0.24)	6.94 (5.73 to 8.2)	0.45 (-0.33 to 1.24)	95.73 (79.48 to 113.18)	0.38 (-0.26 to 1.02)
Luxembourg	49.97 (33.16 to 72.18)	0.42 (0.4 to 0.44)	60.3 (39.82 to 88.92)	0.33 (0.29 to 0.36)	5.42 (4.41 to 6.35)	-0.3 (-0.76 to 0.16)	72.76 (60.25 to 84.96)	-0.4 (-0.82 to 0.03)
Madagascar	0.24 (0.14 to 0.39)	-0.07 (-0.08 to -0.06)	0.43 (0.22 to 0.74)	-0.32 (-0.33 to -0.31)	3.35 (0.92 to 6.54)	0.8 (0.58 to 1.02)	47.55 (13.08 to 91.38)	0.7 (0.54 to 0.86)
Malawi	0.04 (0.02 to 0.07)	-0.7 (-0.81 to -0.58)	0.08 (0.03 to 0.15)	-0.91 (-1.08 to -0.74)	4.29 (1.14 to 8.42)	1.45 (1.28 to 1.62)	61.9 (16.17 to 119.83)	1.49 (1.31 to 1.66)
Malaysia	1.88 (1.21 to 2.77)	-0.69 (-0.84 to -0.54)	2.59 (1.6 to 3.88)	-0.55 (-0.7 to -0.4)	4.72 (3.1 to 6.97)	-0.03 (-0.35 to 0.29)	70.27 (46.6 to 102.88)	-0.38 (-0.69 to -0.07)
Maldives	0.49 (0.29 to 0.76)	-0.52 (-0.56 to -0.48)	0.86 (0.47 to 1.38)	-0.68 (-0.71 to -0.64)	1.39 (0.73 to 2.17)	-1.02 (-1.19 to -0.85)	19.09 (10.22 to 29.99)	-1.36 (-1.76 to -0.96)
Mali	1.57 (0.99 to 2.32)	0.09 (0.06 to 0.12)	2.04 (1.27 to 3.13)	0.03 (0.01 to 0.05)	1.26 (0.4 to 2.49)	1.69 (1.51 to 1.86)	18.49 (5.64 to 36.54)	1.8 (1.64 to 1.96)
Malta	50.09 (32.66 to 72.82)	-0.13 (-0.25 to	61.84 (40.63 to 90.49)	-0.22 (-0.31 to	4.86 (3.92 to 5.72)	-0.49 (-1.21 to	67.58 (55.88 to 79.22)	-0.47 (-1.17 to

© 2025 Wang R, et al. *JAMA Dermatol*.

		-0.01)		-0.13)		0.24)		0.23)
Marshall Islands	0.02 (0 to 0.04)	-0.02 (-0.04 to -0.01)	0.05 (0.01 to 0.12)	-0.07 (-0.09 to -0.05)	6.82 (4.13 to 10.95)	0.93 (0.85 to 1.01)	87.97 (52.8 to 141.43)	0.94 (0.85 to 1.03)
Mauritania	0.04 (0.01 to 0.07)	0.12 (0.11 to 0.14)	0.07 (0.03 to 0.16)	-0.05 (-0.08 to -0.02)	1.27 (0.41 to 2.38)	1.61 (1.43 to 1.79)	18.2 (5.71 to 34.54)	1.67 (1.5 to 1.84)
Mauritius	0.4 (0.24 to 0.62)	0.2 (0.18 to 0.22)	0.73 (0.4 to 1.16)	0.07 (0.01 to 0.13)	1.63 (1.37 to 1.89)	2.01 (-1.71 to 5.86)	25.16 (21.39 to 29.22)	2.05 (-1.68 to 5.93)
Mexico	8.04 (5.49 to 11.42)	-0.02 (-0.04 to 0.01)	13.87 (8.96 to 19.9)	-0.23 (-0.26 to -0.21)	8.58 (7.53 to 9.55)	-1.52 (-2.17 to -0.87)	113.29 (99.55 to 126.68)	-1.73 (-2.34 to -1.11)
Micronesia (Federated States of)	0.02 (0 to 0.04)	-0.07 (-0.08 to -0.05)	0.05 (0.01 to 0.12)	-0.12 (-0.15 to -0.1)	6.77 (4.09 to 10.61)	0.68 (0.61 to 0.75)	87.94 (52.99 to 139.77)	0.67 (0.61 to 0.73)
Monaco	49.07 (32.26 to 71.26)	0.2 (0.18 to 0.21)	61.95 (40.36 to 91.24)	0.23 (0.2 to 0.25)	2.85 (1.53 to 4.79)	0.4 (0.25 to 0.56)	41.3 (23.76 to 67.44)	0.2 (0.06 to 0.35)
Mongolia	0.26 (0.15 to 0.41)	-0.11 (-0.14 to -0.09)	0.55 (0.3 to 0.9)	-0.25 (-0.27 to -0.22)	5.79 (3.45 to 8.89)	8.25 (7.5 to 9.02)	79.7 (48.22 to 120.72)	8.09 (7.37 to 8.82)
Montenegro	23.73 (15.8 to 34.83)	0.08 (0.04 to 0.11)	41.15 (26.79 to 60.06)	-0.02 (-0.05 to 0.01)	6.47 (4.74 to 8.59)	0.98 (0.16 to 1.81)	82.28 (60.82 to 109.12)	0.78 (0.27 to 1.29)
Morocco	0.67 (0.41 to 1)	-0.14	1.46 (0.85 to 2.22)	-0.21	0.1 (0.02 to 0.73)	3.43	1.44 (0.4 to 10.27)	3.08

© 2025 Wang R, et al. *JAMA Dermatol*.

		(-0.22 to -0.06)		(-0.26 to -0.16)		(2.95 to 3.91)		(2.81 to 3.36)
Mozambique	0.23 (0.13 to 0.37)	-0.09 (-0.11 to -0.08)	0.41 (0.21 to 0.7)	-0.32 (-0.34 to -0.3)	4.55 (1.11 to 9.22)	1.45 (1.34 to 1.56)	65.03 (15.64 to 128.58)	1.5 (1.38 to 1.62)
Myanmar	0.39 (0.23 to 0.59)	-0.21 (-0.23 to -0.19)	0.68 (0.38 to 1.07)	-0.38 (-0.41 to -0.34)	4.16 (2.53 to 6.23)	0.43 (0.39 to 0.47)	64.18 (38.88 to 95.47)	0.29 (0.25 to 0.32)
Namibia	15.54 (10.48 to 22.26)	0.64 (0.2 to 1.07)	23.35 (15.49 to 33.85)	0.25 (-0.05 to 0.55)	4.81 (2.88 to 8.2)	1.23 (1.14 to 1.32)	71.94 (43.26 to 122.95)	1.28 (1.19 to 1.36)
Nauru	0.02 (0 to 0.04)	-0.06 (-0.06 to -0.06)	0.05 (0.01 to 0.11)	-0.06 (-0.06 to -0.06)	7.07 (3.95 to 11.67)	0.52 (0.49 to 0.55)	91.85 (50.83 to 153)	0.47 (0.44 to 0.49)
Nepal	0.02 (0 to 0.04)	-0.03 (-0.05 to -0.02)	0.03 (0.01 to 0.07)	-0.24 (-0.26 to -0.22)	3.49 (1.92 to 5.89)	0.95 (0.88 to 1.02)	47.91 (26.85 to 80.68)	0.82 (0.75 to 0.88)
Netherlands	81.42 (53.91 to 119.1)	0.59 (0.44 to 0.73)	95.33 (63.27 to 137.74)	0.49 (0.32 to 0.67)	4.74 (3.88 to 5.52)	0.29 (-0.15 to 0.73)	63.23 (52.8 to 72.83)	0.06 (-0.35 to 0.47)
New Zealand	444.12 (290.81 to 639.49)	0.28 (0.26 to 0.31)	538.09 (356.22 to 788.63)	0.26 (0.23 to 0.28)	20.1 (16.4 to 23.45)	2.54 (1.41 to 3.68)	282.64 (234.59 to 329.14)	1.72 (0.45 to 3)
Nicaragua	7.93 (5.47 to 11.44)	0.03 (0.02 to 0.04)	13.5 (8.76 to 19.61)	-0.15 (-0.2 to -0.11)	5.54 (3.59 to 8.22)	0.48 (0.18 to 0.79)	78.41 (51.18 to 116.21)	0.5 (0.21 to 0.79)

© 2025 Wang R, et al. *JAMA Dermatol*.

Niger	0.04 (0.01 to 0.07)	-0.1 (-0.13 to -0.07)	0.07 (0.02 to 0.15)	-0.34 (-0.41 to -0.26)	1 (0.29 to 1.99)	1.56 (1.37 to 1.76)	14.34 (4.04 to 28.93)	1.64 (1.46 to 1.81)
Nigeria	0.02 (0 to 0.04)	0.08 (0.05 to 0.11)	0.03 (0.01 to 0.08)	-0.03 (-0.09 to 0.04)	1.22 (0.4 to 1.77)	1.89 (1.77 to 2.01)	17.18 (5.36 to 25.44)	1.96 (1.84 to 2.07)
Niue	0.02 (0 to 0.04)	0 (0 to 0.01)	0.05 (0.01 to 0.11)	0 (0 to 0.01)	6.7 (4.01 to 10.24)	0.52 (0.36 to 0.69)	85.63 (50.69 to 131.65)	0.54 (0.4 to 0.68)
North Macedonia	24.42 (16.28 to 35.86)	-0.01 (-0.04 to 0.02)	43.27 (28.56 to 62.53)	-0.13 (-0.17 to -0.08)	13.75 (10.33 to 18.53)	0.68 (0.27 to 1.08)	169.5 (126.69 to 230.3)	0.23 (-0.07 to 0.53)
Northern Mariana Islands	0.02 (0 to 0.04)	-0.01 (-0.03 to 0)	0.05 (0.01 to 0.12)	-0.02 (-0.04 to 0.01)	10.2 (5.72 to 15.72)	0.74 (-0.04 to 1.52)	128.29 (70.8 to 197.3)	0.8 (-1.43 to 3.08)
Norway	51.56 (33.81 to 76.43)	0.24 (0.23 to 0.25)	65.51 (43.07 to 96.9)	0.25 (0.24 to 0.27)	4.97 (4.09 to 5.54)	1.39 (-0.31 to 3.13)	64.26 (54.58 to 71.15)	1.05 (-0.47 to 2.6)
Oman	6.64 (4.59 to 9.41)	0.01 (-0.02 to 0.05)	8.55 (5.82 to 12.07)	-0.04 (-0.11 to 0.04)	2.17 (1.33 to 3.45)	1.33 (0.91 to 1.76)	30.24 (18.82 to 47.54)	0.92 (0.6 to 1.24)
Pakistan	0.02 (0 to 0.04)	-1.78 (-1.83 to -1.73)	0.03 (0.01 to 0.08)	-1.96 (-2 to -1.92)	4.15 (2.75 to 6.21)	0.81 (0.76 to 0.86)	57.86 (38.83 to 86.5)	0.73 (0.68 to 0.79)
Palau	0.02 (0 to 0.04)	0.01 (0 to 0.01)	0.05 (0.01 to 0.12)	0.01 (0 to 0.01)	15.72 (9.7 to 24.92)	0.17 (0.07 to	190.64 (118.26 to 300.08)	0.06 (-0.01 to

© 2025 Wang R, et al. *JAMA Dermatol*.

						0.27)		0.14)
Palestine	1.74 (1.11 to 2.63)	-0.06 (-0.12 to 0)	3.3 (2.02 to 4.97)	-0.18 (-0.25 to -0.12)	5.52 (2.9 to 8.13)	0.33 (-0.03 to 0.69)	73.41 (38.86 to 107.87)	0.32 (-0.02 to 0.67)
Panama	7.71 (5.32 to 10.97)	-3.03 (-3.16 to -2.89)	13.58 (9.03 to 19.38)	-2.14 (-2.25 to -2.03)	3.86 (2.91 to 4.78)	-0.48 (-0.89 to -0.08)	54.46 (41.16 to 67.35)	-0.48 (-0.91 to -0.05)
Papua New Guinea	0.02 (0 to 0.04)	0 (-0.02 to 0.01)	0.05 (0.01 to 0.12)	-0.08 (-0.11 to -0.06)	4.77 (2.37 to 8.42)	0.82 (0.75 to 0.9)	63.74 (31.6 to 112.42)	0.83 (0.75 to 0.9)
Paraguay	0.01 (0 to 0.03)	-1.47 (-1.56 to -1.37)	0.02 (0 to 0.06)	-1.64 (-1.79 to -1.48)	10.3 (6.94 to 14.17)	1.13 (0.89 to 1.36)	145.26 (97.57 to 201.15)	1.15 (0.94 to 1.36)
Peru	0.58 (0.36 to 0.87)	-3.77 (-3.85 to -3.7)	1.17 (0.64 to 1.84)	-3.27 (-3.39 to -3.15)	7.87 (4.43 to 11.59)	1.61 (0.97 to 2.26)	107.5 (59.97 to 158.56)	1.51 (0.8 to 2.23)
Philippines	2.46 (1.55 to 3.66)	-0.22 (-0.37 to -0.08)	3.16 (1.92 to 4.85)	-0.23 (-0.36 to -0.09)	3.86 (3.1 to 5.03)	0.03 (-0.19 to 0.25)	61.32 (48.86 to 78.24)	0.21 (0.01 to 0.42)
Poland	16.3 (10.73 to 24.15)	0.31 (0.26 to 0.35)	29.01 (18.45 to 42.35)	0.24 (0.22 to 0.26)	1.51 (1.29 to 1.66)	-7.6 (-8.52 to -6.68)	20.54 (17.86 to 22.71)	-7.42 (-8.07 to -6.75)
Portugal	61.17 (41.57 to 86.84)	3.16 (2.89 to 3.43)	83.13 (56.33 to 120.03)	2.23 (2.06 to 2.4)	8.35 (6.8 to 9.6)	-1.04 (-2.17 to 0.11)	108.11 (89.75 to 123.62)	-1 (-2.04 to 0.04)
Puerto Rico	0.29 (0.17 to 0.45)	-0.08	0.51 (0.26 to 0.86)	-0.09	8.07 (6.29 to 9.9)	3.25	114.59 (89.59 to	3.52

© 2025 Wang R, et al. *JAMA Dermatol*.

		(-0.08 to -0.07)		(-0.09 to -0.08)		(2.38 to 4.12)	141.11)	(2.51 to 4.55)
Qatar	0.42 (0.16 to 0.8)	-0.12 (-0.41 to 0.18)	0.88 (0.34 to 1.68)	-0.19 (-0.46 to 0.07)	1.53 (0.82 to 2.49)	0.64 (-0.53 to 1.82)	21.69 (11.57 to 36.14)	0.41 (-0.69 to 1.52)
Republic of Korea	5.54 (4.27 to 7.06)	1.48 (1.4 to 1.55)	11.62 (8.44 to 15.2)	1.23 (1.19 to 1.28)	5.05 (3.6 to 7.82)	-2.92 (-3.12 to -2.73)	58.38 (41.99 to 95.6)	-3.28 (-3.47 to -3.08)
Republic of Moldova	19.78 (13.09 to 28.92)	0.08 (0.07 to 0.1)	34.22 (22.46 to 49.15)	0.01 (-0.02 to 0.03)	6.87 (5.68 to 8.06)	-0.91 (-1.72 to -0.1)	109.09 (91.66 to 127.4)	-0.5 (-1.28 to 0.28)
Romania	13.27 (8.74 to 19.39)	0.33 (0.31 to 0.34)	23.79 (15.38 to 34.73)	0.14 (0.13 to 0.16)	9.81 (8.19 to 11.51)	-1.97 (-3.1 to -0.83)	138.79 (116.24 to 163.2)	-1.84 (-2.84 to -0.83)
Russian Federation	16.28 (10.83 to 23.58)	0.78 (0.74 to 0.83)	27.59 (17.91 to 39.63)	0.85 (0.81 to 0.89)	6.55 (5.8 to 7.12)	0.2 (-0.31 to 0.72)	94.64 (84.99 to 102.61)	0.19 (-0.24 to 0.62)
Rwanda	0.23 (0.13 to 0.37)	-0.1 (-0.13 to -0.08)	0.4 (0.21 to 0.7)	-0.33 (-0.36 to -0.31)	4.36 (1.21 to 8.89)	0.89 (0.76 to 1.02)	60.91 (16.96 to 122.82)	0.79 (0.71 to 0.87)
Saint Kitts and Nevis	0.28 (0.16 to 0.44)	0.01 (-0.01 to 0.03)	0.5 (0.25 to 0.84)	0.01 (0 to 0.03)	12.8 (10.4 to 15.3)	8.84 (6.81 to 10.9)	173.23 (140.21 to 208.85)	8.84 (6.69 to 11.03)
Saint Lucia	0.3 (0.17 to 0.45)	0.18 (0.16 to 0.2)	0.55 (0.29 to 0.89)	0.03 (0 to 0.06)	4.15 (3.33 to 5.03)	2.95 (1.45 to 4.47)	54.33 (43.22 to 66.42)	3.06 (1.66 to 4.48)

© 2025 Wang R, et al. *JAMA Dermatol*.

Saint Vincent and the Grenadines	0.31 (0.18 to 0.48)	0.35 (0.33 to 0.36)	0.57 (0.3 to 0.94)	0.21 (0.18 to 0.24)	10.41 (8.73 to 12.16)	7.28 (6.01 to 8.56)	137.31 (114.6 to 161.41)	7.12 (5.94 to 8.32)
Samoa	0.02 (0 to 0.04)	-0.39 (-0.43 to -0.35)	0.05 (0.01 to 0.12)	-0.46 (-0.54 to -0.39)	6.84 (4.11 to 10.89)	0.64 (0.6 to 0.67)	88.35 (52.97 to 141.37)	0.62 (0.59 to 0.66)
San Marino	50.69 (33.25 to 73.73)	0.16 (0.15 to 0.18)	64.11 (41.69 to 94.59)	0.19 (0.17 to 0.21)	2.73 (1.51 to 4.39)	-1.86 (-2.53 to -1.19)	38.61 (22.52 to 60.7)	-1.68 (-2.23 to -1.12)
Sao Tome and Principe	0.04 (0.01 to 0.07)	0.01 (0 to 0.02)	0.07 (0.02 to 0.14)	-0.2 (-0.23 to -0.17)	0.21 (0.13 to 0.31)	0.33 (0.19 to 0.46)	2.78 (1.72 to 4.16)	0.41 (0.25 to 0.57)
Saudi Arabia	4.88 (3.84 to 6)	1.37 (0.94 to 1.81)	7.68 (5.38 to 10.75)	0.99 (0.8 to 1.18)	1.35 (0.85 to 2.05)	1.74 (1.36 to 2.12)	18.65 (11.91 to 28.19)	1.65 (1.32 to 1.99)
Senegal	0.15 (0.06 to 0.31)	-0.01 (-0.02 to 0)	0.28 (0.1 to 0.59)	-0.26 (-0.3 to -0.23)	1.29 (0.43 to 2.33)	1.97 (1.75 to 2.2)	18.71 (5.99 to 34.17)	2.03 (1.81 to 2.24)
Serbia	21.82 (14.36 to 32.48)	0.25 (0.21 to 0.29)	39.45 (26.05 to 57.01)	0.08 (0.05 to 0.1)	13.63 (10.12 to 17.95)	-1.25 (-1.82 to -0.69)	181.43 (134.83 to 238.15)	-1.03 (-1.28 to -0.77)
Seychelles	0.02 (0 to 0.04)	0.51 (0.46 to 0.55)	0.03 (0.01 to 0.08)	0.26 (0.2 to 0.31)	3.27 (2.29 to 4.41)	1.63 (1.19 to 2.08)	49.19 (34.24 to 66.86)	1.68 (1.17 to 2.2)
Sierra Leone	0.04 (0.01 to 0.07)	-0.07 (-0.09 to	0.07 (0.03 to 0.16)	-0.27 (-0.3 to	1.06 (0.33 to 1.92)	1.56 (1.37 to	15.38 (4.61 to 28.25)	1.65 (1.47 to

© 2025 Wang R, et al. *JAMA Dermatol*.

		-0.05)		-0.24)		1.76)		1.83)
Singapore	8.82 (5.8 to 12.9)	-0.15 (-0.17 to -0.12)	15.14 (9.69 to 22.41)	-0.17 (-0.2 to -0.15)	2.12 (1.69 to 2.48)	-1.94 (-2.88 to -1)	27.7 (22.64 to 32.22)	-2.23 (-3.21 to -1.24)
Slovakia	30.96 (20.57 to 44.91)	-0.53 (-0.69 to -0.37)	48.22 (31.57 to 70.16)	-0.46 (-0.49 to -0.42)	6.42 (4.73 to 9.05)	-1.21 (-1.39 to -1.03)	86.57 (64.07 to 122.34)	-1.24 (-1.39 to -1.08)
Slovenia	47.6 (34.62 to 61.81)	1.96 (1.88 to 2.04)	66.03 (45.86 to 93.63)	1.43 (1.37 to 1.5)	4.84 (3.89 to 5.74)	-1.66 (-3.21 to -0.1)	62.37 (51.05 to 73.72)	-1.79 (-3.13 to -0.43)
Solomon Islands	0.02 (0 to 0.04)	-0.06 (-0.09 to -0.04)	0.05 (0.01 to 0.12)	-0.12 (-0.21 to -0.04)	5.82 (3.38 to 10.03)	0.84 (0.77 to 0.91)	76.21 (43.66 to 132.23)	0.82 (0.69 to 0.96)
Somalia	0.23 (0.13 to 0.37)	-0.16 (-0.17 to -0.15)	0.4 (0.2 to 0.67)	-0.42 (-0.46 to -0.39)	3.02 (0.69 to 6.43)	0.77 (0.66 to 0.89)	45.25 (10.59 to 94.97)	0.86 (0.72 to 0.99)
South Africa	65.9 (44.16 to 93.77)	1.35 (1.01 to 1.7)	79.44 (54.01 to 115.48)	1.24 (0.92 to 1.55)	7.79 (5.5 to 9.16)	1.26 (0.94 to 1.59)	114.63 (80.94 to 134.15)	1.34 (0.97 to 1.71)
South Sudan	0.25 (0.14 to 0.4)	-0.14 (-0.15 to -0.13)	0.46 (0.23 to 0.79)	-0.33 (-0.36 to -0.3)	3.8 (0.91 to 7.9)	1.23 (1.16 to 1.31)	54.85 (13.22 to 112.92)	1.19 (1.12 to 1.26)
Spain	67.67 (45.72 to 96.92)	0.8 (0.68 to 0.91)	82.94 (56.38 to 118.81)	0.56 (0.51 to 0.61)	6.46 (5.22 to 7.49)	-1.45 (-1.67 to -1.23)	87.43 (72.58 to 100.69)	-1.43 (-1.98 to -0.87)
Sri Lanka	0.38 (0.23 to 0.56)	-1.59	0.65 (0.36 to 1.01)	-1.65	5.31 (3.36 to 8.39)	-1.97	78.6 (48.85 to 126.33)	-1.98

© 2025 Wang R, et al. *JAMA Dermatol*.

		(-1.78 to -1.4)		(-1.9 to -1.39)		(-2.26 to -1.69)		(-2.26 to -1.69)
Sudan	2.3 (1.45 to 3.36)	0.27 (0.25 to 0.28)	3.82 (2.31 to 5.81)	0.13 (0.11 to 0.14)	0.09 (0.02 to 0.72)	3.19 (3.07 to 3.31)	1.49 (0.44 to 10.21)	2.58 (2.5 to 2.67)
Suriname	0.29 (0.17 to 0.45)	-0.15 (-0.18 to -0.13)	0.52 (0.27 to 0.86)	-0.35 (-0.38 to -0.33)	3.29 (1.99 to 5.12)	1.27 (0.88 to 1.66)	47.06 (28.35 to 72.95)	1.16 (0.79 to 1.54)
Sweden	52.17 (34.48 to 76.86)	0.24 (0.23 to 0.25)	66.05 (43.42 to 98.76)	0.24 (0.23 to 0.26)	3.81 (3.11 to 4.42)	0.57 (-0.1 to 1.25)	53.2 (44.25 to 61.42)	0.29 (-0.36 to 0.95)
Switzerland	67.86 (45.31 to 97.19)	-0.37 (-0.44 to -0.29)	74.23 (49.86 to 105.23)	-0.35 (-0.5 to -0.21)	5.23 (4.13 to 6.11)	1.3 (1.15 to 1.46)	68.95 (56.26 to 80.04)	0.66 (0.25 to 1.07)
Syrian Arab Republic	2.18 (1.37 to 3.27)	0.13 (0.11 to 0.15)	4.05 (2.48 to 6.12)	0 (-0.04 to 0.04)	0.02 (0.02 to 0.03)	0.53 (-0.22 to 1.29)	0.48 (0.34 to 0.65)	0.25 (0.02 to 0.48)
Taiwan (Province of China)	4.03 (2.84 to 5.6)	0.57 (0.2 to 0.94)	6.28 (4.23 to 9.04)	0.05 (-0.21 to 0.3)	3.52 (2.91 to 4.11)	-1.94 (-3.18 to -0.68)	51.08 (42.81 to 59.27)	-2.05 (-3.1 to -0.99)
Tajikistan	0.29 (0.17 to 0.44)	0.29 (0.26 to 0.33)	0.63 (0.35 to 1.02)	0.25 (0.15 to 0.34)	4.29 (2.64 to 6.55)	-0.88 (-1.53 to -0.22)	61.51 (38.21 to 93.5)	-0.77 (-1.36 to -0.18)
Thailand	4.49 (3.03 to 6.48)	-1.41 (-1.56 to -1.25)	5.01 (3.35 to 7.05)	-1.28 (-1.46 to -1.1)	5.64 (3.67 to 8.76)	-0.66 (-0.88 to -0.44)	81.23 (53.07 to 127.83)	-0.66 (-0.89 to -0.43)

© 2025 Wang R, et al. *JAMA Dermatol*.

Timor-Leste	0.46 (0.27 to 0.7)	-0.03 (-0.07 to 0.01)	0.8 (0.44 to 1.27)	-0.19 (-0.25 to -0.13)	4.3 (2.55 to 7)	1.03 (0.93 to 1.13)	66.34 (39.36 to 107.72)	0.98 (0.88 to 1.08)
Togo	0.03 (0.01 to 0.07)	-0.3 (-0.36 to -0.23)	0.07 (0.02 to 0.14)	-0.58 (-0.7 to -0.45)	1.18 (0.42 to 2.05)	1.55 (1.29 to 1.81)	17.09 (5.68 to 29.95)	1.62 (1.37 to 1.87)
Tokelau	0.02 (0 to 0.04)	0 (-0.01 to 0)	0.05 (0.01 to 0.12)	-0.01 (-0.02 to 0)	6.82 (4.08 to 10.99)	0.35 (0.31 to 0.39)	85.63 (51.41 to 137.9)	0.29 (0.21 to 0.37)
Tonga	0.02 (0 to 0.04)	-0.06 (-0.07 to -0.05)	0.05 (0.01 to 0.12)	-0.09 (-0.1 to -0.08)	28.63 (17.62 to 44.57)	0.3 (0.12 to 0.47)	348.46 (216.01 to 541.19)	0.23 (0.05 to 0.41)
Trinidad and Tobago	2.66 (1.78 to 3.84)	-0.2 (-0.23 to -0.16)	3.29 (2.17 to 4.75)	-0.23 (-0.3 to -0.15)	2.68 (2.06 to 3.36)	-0.61 (-1.11 to -0.11)	37.92 (28.95 to 48.09)	-0.51 (-1.04 to 0.04)
Tunisia	3.28 (2.1 to 4.83)	-0.49 (-0.78 to -0.2)	5.35 (3.34 to 7.98)	-0.5 (-0.59 to -0.41)	0.1 (0.02 to 0.8)	2.65 (2.25 to 3.06)	1.62 (0.48 to 11.3)	1.9 (1.65 to 2.15)
Turkmenistan	0.26 (0.16 to 0.41)	0.14 (0.11 to 0.18)	0.56 (0.31 to 0.93)	0.07 (0 to 0.14)	8.57 (6.64 to 11.09)	8.57 (6.9 to 10.28)	130.11 (100.61 to 169.48)	8.7 (7 to 10.43)
Tuvalu	0.02 (0 to 0.04)	0.01 (0 to 0.01)	0.05 (0.01 to 0.11)	0.01 (0 to 0.01)	6.86 (4.23 to 11.02)	0.68 (0.6 to 0.76)	87.74 (53.85 to 140.84)	0.64 (0.58 to 0.69)
Turkey	9.75 (6.45 to 14.2)	-0.74 (-1.03 to	11.83 (7.85 to 17.14)	-0.63 (-0.83 to	7.15 (4.83 to 10.37)	-0.17 (-0.61 to	93.51 (63.17 to 135.29)	-0.29 (-0.74 to

© 2025 Wang R, et al. *JAMA Dermatol*.

		-0.45)		-0.43)		0.27)		0.16)
Uganda	3.18 (2.08 to 4.62)	0.41 (0.39 to 0.43)	3.5 (2.29 to 5.06)	0.31 (0.29 to 0.33)	3.74 (1.01 to 7.52)	1.56 (1.53 to 1.59)	52.64 (14.1 to 104.87)	1.5 (1.47 to 1.53)
Ukraine	20.32 (13.54 to 30.03)	0.26 (0.25 to 0.27)	32.55 (20.84 to 47.61)	0.22 (0.2 to 0.24)	5.72 (4.3 to 7.38)	0.55 (-0.1 to 1.2)	86.82 (65.17 to 112.21)	0.46 (0 to 0.92)
United Arab Emirates	3.07 (1.92 to 4.64)	1.26 (1.18 to 1.35)	5.76 (3.51 to 8.69)	1.13 (1.07 to 1.19)	5.73 (3.62 to 8.71)	0.91 (-2.45 to 4.38)	81.52 (52.4 to 122.64)	0.87 (-2.13 to 3.97)
United Kingdom	54.93 (36.62 to 79.65)	0.54 (0.46 to 0.61)	69.12 (46.11 to 101.16)	0.51 (0.45 to 0.57)	7.33 (6.32 to 7.86)	0.75 (-0.84 to 2.36)	96.96 (86.03 to 103.58)	0.42 (-1.17 to 2.04)
United Republic of Tanzania	0.24 (0.14 to 0.39)	-0.02 (-0.02 to -0.01)	0.43 (0.22 to 0.74)	-0.2 (-0.22 to -0.19)	3.87 (1.07 to 7.66)	0.95 (0.86 to 1.04)	54.53 (15.12 to 107.09)	0.92 (0.84 to 0.99)
United States of America	2226.83 (1877.72 to 2598.71)	2.74 (2.6 to 2.88)	2522.1 (2036.32 to 3194.44)	3.01 (2.87 to 3.14)	6.91 (5.96 to 7.46)	0.67 (0.18 to 1.17)	198.09 (160.88 to 246.23)	1.45 (1.28 to 1.62)
United States Virgin Islands	0.29 (0.17 to 0.46)	0.04 (0.03 to 0.05)	0.51 (0.26 to 0.85)	0.04 (0.03 to 0.05)	3.12 (1.82 to 4.89)	1.27 (0.73 to 1.82)	39.82 (22.91 to 63.35)	0.93 (0.35 to 1.52)
Uruguay	32.29 (21.36 to 47.12)	0.26 (0.11 to 0.41)	52.7 (34.7 to 77.06)	0.04 (-0.03 to 0.11)	6.2 (5.21 to 7.14)	0.56 (-0.03 to 1.15)	85.51 (72.88 to 98.16)	0.53 (-0.03 to 1.09)
Uzbekistan	0.27 (0.16 to 0.41)	0.18	0.59 (0.32 to 0.97)	0.13	6.12 (4.78 to 7.72)	0.74	88.56 (69.39 to 111.62)	1.07

© 2025 Wang R, et al. *JAMA Dermatol*.

		(0.16 to 0.21)		(0.07 to 0.19)		(0.32 to 1.16)		(0.85 to 1.29)
Vanuatu	0.02 (0 to 0.04)	-0.07 (-0.07 to -0.06)	0.05 (0.01 to 0.12)	-0.13 (-0.14 to -0.11)	5.72 (3.31 to 9.42)	0.76 (0.71 to 0.82)	74.75 (43.36 to 122.56)	0.76 (0.71 to 0.8)
Venezuela (Bolivarian Republic of)	7.94 (5.41 to 11)	0.01 (-0.01 to 0.02)	13.51 (8.94 to 19.43)	-0.19 (-0.21 to -0.17)	14.63 (11.11 to 18.66)	0.39 (-0.03 to 0.81)	204.94 (155.69 to 263.39)	0.29 (-0.09 to 0.67)
Viet Nam	0.12 (0.06 to 0.2)	-2.44 (-2.51 to -2.37)	0.23 (0.11 to 0.41)	-2.58 (-2.66 to -2.49)	5.23 (3.22 to 7.79)	0.93 (0.89 to 0.97)	80.31 (49.35 to 119.14)	0.85 (0.81 to 0.89)
Yemen	1.95 (1.23 to 2.9)	0.6 (0.58 to 0.62)	3.64 (2.2 to 5.47)	0.5 (0.44 to 0.55)	0.1 (0.02 to 0.73)	3.49 (3.11 to 3.88)	1.54 (0.45 to 10.45)	2.94 (2.76 to 3.11)
Zambia	0.24 (0.13 to 0.38)	-0.22 (-0.24 to -0.2)	0.43 (0.22 to 0.74)	-0.48 (-0.51 to -0.45)	4.3 (1.24 to 7.9)	1.15 (1 to 1.3)	62.19 (17.73 to 112.94)	1.13 (1.04 to 1.22)
Zimbabwe	8.97 (6.04 to 13.03)	-1.05 (-1.22 to -0.88)	16.36 (10.71 to 23.68)	-0.98 (-1.09 to -0.88)	4.77 (2.86 to 8.27)	1.22 (1.07 to 1.36)	72.45 (43.92 to 126.19)	1.32 (1.1 to 1.54)

Abbreviations: UI, uncertainty interval; ASR, age-standerised rate per 100,000; CI, confidence interval; DALYs, disability-adjusted life-year.

© 2025 Wang R, et al. *JAMA Dermatol*.

eTable 6. Burden of basal cell carcinoma in 204 countries and territories in 2021 and the average annual percent change (AAPC) from 1990 to 2021.

Country	Incidence		Prevalence		DALYs	
	ASR (95% UI)	AAPC (95% CI)	ASR (95% UI)	AAPC (95% CI)	ASR (95% UI)	AAPC (95% CI)
Afghanistan	29.45 (20.37 to 41.1)	-0.14 (-0.15 to -0.12)	3.64 (2.41 to 5.17)	-0.17 (-0.19 to -0.15)	0.02 (0.01 to 0.03)	-0.17 (-0.19 to -0.16)
Albania	177.7 (125.37 to 242.8)	0.05 (0.05 to 0.06)	24.61 (17.03 to 33.49)	0.03 (0.02 to 0.04)	0.1 (0.03 to 0.23)	0.01 (-0.09 to 0.11)
Algeria	34.52 (24.48 to 47.62)	-0.24 (-0.32 to -0.16)	5.09 (3.43 to 7.15)	-0.26 (-0.34 to -0.17)	0.02 (0.01 to 0.05)	-0.26 (-0.36 to -0.16)
American Samoa	0.17 (0.04 to 0.37)	0 (0 to 0)	0.03 (0.01 to 0.06)	0 (0 to 0)	0 (0 to 0)	0 (0 to 0)
Andorra	223.93 (157.88 to 313.96)	0.03 (-0.01 to 0.07)	33.05 (22.43 to 45.5)	-0.04 (-0.07 to -0.01)	0.13 (0.03 to 0.32)	-0.05 (-0.23 to 0.13)
Angola	26.11 (18.2 to 35.98)	-0.1 (-0.12 to -0.09)	3.22 (2.14 to 4.53)	-0.13 (-0.15 to -0.11)	0.01 (0.01 to 0.03)	-0.13 (-0.15 to -0.12)
Antigua and Barbuda	22.2 (15.28 to 30.69)	0.16 (0.13 to 0.19)	3.18 (2.12 to 4.53)	0.11 (0.09 to 0.12)	0.01 (0.01 to 0.03)	0.11 (0.1 to 0.12)
Argentina	146.36 (104.41 to 201.11)	0 (-0.05 to 0.06)	20.68 (14.09 to 28.45)	-0.05 (-0.09 to -0.01)	0.09 (0.03 to 0.19)	-0.01 (-0.12 to 0.1)
Armenia	147.26 (104.07 to 200.31)	0.03 (0.03 to 0.04)	20.37 (14.17 to 28.01)	0.01 (0 to 0.02)	0.09 (0.03 to 0.18)	0.02 (-0.01 to 0.05)
Australia	161.91 (114.46 to 222.5)	-0.05 (-0.09 to -0.02)	24.75 (16.7 to 33.9)	-0.08 (-0.1 to -0.05)	0.1 (0.03 to 0.24)	-0.03 (-0.31 to 0.26)
Austria	184.87 (131.01 to 255.88)	0.32	27.07 (18.47 to 38.03)	0.23	0.11 (0.03 to 0.26)	0.18

© 2025 Wang R, et al. *JAMA Dermatol*.

		(0.05 to 0.6)		(-0.01 to 0.47)		(-0.04 to 0.41)
Azerbaijan	151 (108.95 to 205.63)	0.06 (0.05 to 0.06)	18.68 (12.94 to 25.43)	0.04 (0.03 to 0.04)	0.08 (0.03 to 0.17)	0.01 (-0.01 to 0.04)
Bahamas	21.67 (14.89 to 30.42)	0.13 (0.12 to 0.15)	3.18 (2.09 to 4.54)	0.07 (0.05 to 0.09)	0.01 (0.01 to 0.03)	0.08 (0.06 to 0.09)
Bahrain	25.77 (16.02 to 38.98)	0.09 (0.06 to 0.11)	3.47 (2.1 to 5.19)	0.06 (0.03 to 0.09)	0.02 (0.01 to 0.03)	0.05 (0.03 to 0.08)
Bangladesh	0.2 (0.04 to 0.47)	-0.07 (-0.08 to -0.05)	0.03 (0.01 to 0.07)	-0.11 (-0.12 to -0.11)	0 (0 to 0)	-0.12 (-0.12 to -0.11)
Barbados	21.65 (14.97 to 30.44)	0.14 (0.12 to 0.15)	3.24 (2.14 to 4.61)	0.09 (0.06 to 0.13)	0.01 (0.01 to 0.03)	0.09 (0.06 to 0.13)
Belarus	133.77 (95.21 to 182.7)	0.15 (-0.07 to 0.37)	18.22 (12.64 to 24.88)	0.06 (-0.17 to 0.29)	0.08 (0.03 to 0.16)	0.1 (-0.06 to 0.26)
Belgium	204.22 (143.81 to 282.43)	0.21 (0.19 to 0.23)	28.76 (19.19 to 39.8)	0.11 (0.06 to 0.16)	0.12 (0.03 to 0.27)	0.09 (-0.11 to 0.29)
Belize	23.03 (15.79 to 32.07)	0.1 (0.08 to 0.11)	3.53 (2.34 to 5.02)	0.05 (0.03 to 0.06)	0.02 (0.01 to 0.03)	0.05 (0.03 to 0.06)
Benin	15.23 (10.28 to 21.86)	-0.04 (-0.04 to -0.03)	2.04 (1.33 to 2.97)	-0.09 (-0.1 to -0.07)	0.01 (0 to 0.02)	-0.09 (-0.12 to -0.06)
Bermuda	14.7 (9.32 to 22.22)	-0.1 (-0.11 to -0.09)	2.4 (1.43 to 3.58)	-0.13 (-0.15 to -0.11)	0.01 (0 to 0.02)	-0.13 (-0.15 to -0.11)
Bhutan	0.2 (0.04 to 0.46)	0.08 (0.05 to 0.1)	0.03 (0.01 to 0.07)	0.06 (0.03 to 0.09)	0 (0 to 0)	0.06 (0.03 to 0.08)
Bolivia (Plurinational State of)	82.89 (60.44 to 110)	0.04 (0.02 to 0.05)	9.79 (6.87 to 13.37)	0.01 (-0.01 to 0.04)	0.04 (0.02 to 0.09)	0.02 (-0.01 to 0.04)
Bosnia and Herzegovina	178.9 (126.47 to 243.94)	0.05	23.55 (16.23 to 32.38)	0.04	0.1 (0.03 to 0.22)	0.08

© 2025 Wang R, et al. *JAMA Dermatol*.

		(0.04 to 0.06)		(0.03 to 0.05)		(0.03 to 0.13)
Botswana	81.77 (58.08 to 109.88)	-0.03<br>(-0.05 to -0.02)	9.18 (6.33 to 12.6)	-0.06<br>(-0.08 to -0.05)	0.04 (0.02 to 0.08)	-0.06<br>(-0.08 to -0.05)
Brazil	196.74 (153.37 to 245.56)	-2.23<br>(-2.39 to -2.07)	21.2 (16.23 to 27.17)	-2.11<br>(-2.24 to -1.98)	0.09 (0.04 to 0.18)	-2<br>(-2.15 to -1.86)
Brunei Darussalam	33.56 (23.34 to 46.97)	0<br>(-0.01 to 0)	4.68 (3.05 to 6.7)	-0.06<br>(-0.07 to -0.05)	0.02 (0.01 to 0.04)	-0.06<br>(-0.07 to -0.05)
Bulgaria	328.24 (248.05 to 401.47)	1.55<br>(1.26 to 1.83)	36.97 (27.36 to 47.78)	1.31<br>(1.04 to 1.59)	0.15 (0.04 to 0.35)	1.22<br>(0.96 to 1.49)
Burkina Faso	15.26 (10.14 to 21.54)	-0.01<br>(-0.01 to 0)	2 (1.26 to 2.86)	-0.03<br>(-0.04 to -0.02)	0.01 (0 to 0.02)	-0.03<br>(-0.04 to -0.03)
Burundi	20.85 (14.17 to 29.27)	0.11<br>(0.1 to 0.13)	2.63 (1.7 to 3.76)	0.07<br>(0.06 to 0.09)	0.01 (0 to 0.02)	0.08<br>(0.06 to 0.09)
Cabo Verde	14.25 (9.68 to 19.94)	-0.06<br>(-0.07 to -0.05)	2.25 (1.46 to 3.19)	-0.07<br>(-0.08 to -0.07)	0.01 (0 to 0.02)	-0.07<br>(-0.08 to -0.06)
Cambodia	6.19 (3.95 to 8.92)	-0.04<br>(-0.05 to -0.02)	0.8 (0.49 to 1.2)	-0.06<br>(-0.08 to -0.05)	0 (0 to 0.01)	-0.07<br>(-0.09 to -0.05)
Cameroon	15.22 (10.27 to 21.26)	-0.04<br>(-0.05 to -0.02)	2.01 (1.3 to 2.89)	-0.07<br>(-0.09 to -0.06)	0.01 (0 to 0.02)	-0.08<br>(-0.09 to -0.06)
Canada	200.11 (140.24 to 275.29)	0.49<br>(0.39 to 0.58)	30.17 (20.63 to 41.52)	0.38<br>(0.27 to 0.49)	0.13 (0.03 to 0.3)	0.39<br>(0.16 to 0.64)
Central African Republic	26.08 (18.1 to 36.81)	-0.05<br>(-0.06 to -0.04)	2.96 (1.98 to 4.16)	-0.08<br>(-0.08 to -0.07)	0.01 (0.01 to 0.03)	-0.07<br>(-0.08 to -0.07)
Chad	15.97 (10.82 to 22.63)	0.1<br>(0.09 to 0.11)	2.08 (1.34 to 3.03)	0.07<br>(0.05 to 0.09)	0.01 (0 to 0.02)	0.07<br>(0.06 to 0.09)
Chile	130.42 (92.75 to 178.98)	0.08	20.04 (13.64 to 27.62)	0.02	0.08 (0.03 to 0.18)	0.02

© 2025 Wang R, et al. *JAMA Dermatol*.

		(0.05 to 0.12)		(-0.01 to 0.05)		(-0.09 to 0.12)
China	181.56 (141.1 to 229.76)	6.86<br>(6.46 to 7.25)	20.21 (15.41 to 25.93)	6.35<br>(5.99 to 6.72)	0.09 (0.04 to 0.17)	6.27<br>(5.92 to 6.63)
Colombia	174.83 (124.97 to 240.19)	-0.03<br>(-0.04 to -0.03)	22.38 (15.5 to 30.45)	-0.06<br>(-0.07 to -0.04)	0.09 (0.03 to 0.2)	-0.07<br>(-0.14 to 0.01)
Comoros	19.82 (13.38 to 27.69)	-0.04<br>(-0.04 to -0.03)	2.67 (1.77 to 3.82)	-0.07<br>(-0.08 to -0.06)	0.01 (0 to 0.02)	-0.07<br>(-0.08 to -0.07)
Congo	26.62 (18.45 to 36.5)	0.09<br>(0.07 to 0.1)	3.22 (2.12 to 4.45)	0.06<br>(0.05 to 0.07)	0.01 (0.01 to 0.03)	0.06<br>(0.05 to 0.07)
Cook Islands	0.17 (0.04 to 0.37)	0<br>(0 to 0)	0.03 (0.01 to 0.06)	0.01<br>(0.01 to 0.02)	0 (0 to 0)	0.01<br>(0.01 to 0.02)
Costa Rica	201.15 (143.71 to 272.34)	-1.09<br>(-1.4 to -0.78)	25.84 (17.92 to 35.17)	-0.96<br>(-1.07 to -0.86)	0.11 (0.03 to 0.24)	-0.97<br>(-1.23 to -0.71)
Croatia	149.39 (103.32 to 212.33)	0.19<br>(0.16 to 0.23)	20.49 (13.71 to 28.68)	0.12<br>(0.08 to 0.17)	0.09 (0.03 to 0.18)	0.09<br>(-0.05 to 0.23)
Cuba	44.89 (31.63 to 61.95)	-0.58<br>(-0.77 to -0.4)	6.52 (4.39 to 9.13)	-0.54<br>(-0.69 to -0.4)	0.03 (0.01 to 0.06)	-0.55<br>(-0.69 to -0.41)
Cyprus	164.57 (115.87 to 230.97)	0.1<br>(0.08 to 0.11)	22.65 (15.44 to 31.19)	0.03<br>(-0.02 to 0.08)	0.09 (0.03 to 0.21)	-0.03<br>(-0.16 to 0.1)
Czechia	257.33 (184.72 to 354.83)	-0.94<br>(-1.52 to -0.35)	32.61 (22.34 to 44.69)	-0.81<br>(-1.04 to -0.58)	0.13 (0.03 to 0.3)	-0.71<br>(-1.06 to -0.36)
Côte d'Ivoire	10.69 (6.96 to 15.35)	-0.09<br>(-0.11 to -0.07)	1.44 (0.89 to 2.14)	-0.14<br>(-0.16 to -0.12)	0.01 (0 to 0.01)	-0.14<br>(-0.16 to -0.12)
Democratic People's Republic of Korea	0.94 (0.48 to 1.55)	0.08<br>(0.07 to 0.09)	0.14 (0.07 to 0.24)	0.04<br>(0.04 to 0.05)	0 (0 to 0)	0.05<br>(0.04 to 0.05)
Democratic Republic of the Congo	25.7 (17.8 to 35.82)	-0.14	3.19 (2.12 to 4.49)	-0.18	0.01 (0.01 to 0.03)	-0.18

© 2025 Wang R, et al. *JAMA Dermatol*.

		(-0.16 to -0.12)		(-0.2 to -0.16)		(-0.2 to -0.17)
Denmark	250.4 (175.64 to 347.56)	-0.4 (-0.55 to -0.24)	33.53 (22.91 to 46.42)	-0.42 (-0.58 to -0.26)	0.14 (0.03 to 0.33)	-0.4 (-0.64 to -0.16)
Djibouti	20.77 (14.04 to 29.12)	0.1 (0.08 to 0.12)	2.77 (1.81 to 3.91)	0.05 (0.03 to 0.06)	0.01 (0 to 0.02)	0.04 (0.02 to 0.06)
Dominica	21.57 (14.83 to 30.06)	0.16 (0.13 to 0.2)	2.99 (1.98 to 4.17)	0.11 (0.09 to 0.13)	0.01 (0.01 to 0.03)	0.11 (0.09 to 0.13)
Dominican Republic	22.27 (15.52 to 31.33)	-0.03 (-0.05 to -0.02)	3.47 (2.3 to 4.93)	-0.1 (-0.11 to -0.08)	0.02 (0.01 to 0.03)	-0.1 (-0.11 to -0.08)
Ecuador	93.84 (67.28 to 129.38)	-1.55 (-1.62 to -1.49)	12.92 (8.98 to 18)	-1.33 (-1.38 to -1.27)	0.06 (0.02 to 0.12)	-1.32 (-1.46 to -1.17)
Egypt	30 (21.02 to 42.04)	-0.27 (-0.52 to -0.02)	3.94 (2.69 to 5.58)	-0.21 (-0.42 to 0)	0.02 (0.01 to 0.03)	-0.21 (-0.39 to -0.02)
El Salvador	175.48 (121.7 to 240.69)	-0.03 (-0.03 to -0.02)	21.86 (14.85 to 30.14)	-0.04 (-0.05 to -0.04)	0.09 (0.03 to 0.2)	-0.04 (-0.06 to -0.01)
Equatorial Guinea	26.02 (18 to 36.29)	0.01 (0 to 0.02)	3.25 (2.17 to 4.54)	-0.03 (-0.04 to -0.02)	0.01 (0.01 to 0.03)	-0.03 (-0.04 to -0.02)
Eritrea	19.02 (13.07 to 26.6)	0.06 (0.05 to 0.06)	2.33 (1.53 to 3.3)	0.01 (0 to 0.01)	0.01 (0 to 0.02)	0 (0 to 0.01)
Estonia	132.57 (93.54 to 184.22)	-0.27 (-0.3 to -0.24)	19.18 (12.94 to 26.43)	-0.27 (-0.3 to -0.23)	0.08 (0.03 to 0.17)	-0.21 (-0.4 to -0.01)
Eswatini	79.3 (56.39 to 108.3)	-0.09 (-0.1 to -0.07)	8.64 (6.01 to 11.87)	-0.12 (-0.14 to -0.1)	0.04 (0.02 to 0.07)	-0.12 (-0.14 to -0.1)
Ethiopia	21.74 (15 to 30.17)	-0.01 (-0.02 to -0.01)	2.74 (1.82 to 3.88)	-0.02 (-0.03 to -0.01)	0.01 (0 to 0.02)	-0.02 (-0.03 to -0.01)
Fiji	0.17 (0.04 to 0.37)	-0.02	0.02 (0 to 0.05)	-0.02	0 (0 to 0)	-0.03

© 2025 Wang R, et al. *JAMA Dermatol*.

		(-0.02 to -0.01)		(-0.03 to -0.02)		(-0.04 to -0.01)
Finland	222.63 (156.76 to 309.27)	0.13 (0.1 to 0.17)	30.75 (20.95 to 42.76)	0.09 (0.04 to 0.13)	0.13 (0.03 to 0.28)	0.06 (-0.13 to 0.24)
France	254.94 (178.72 to 353.27)	-0.71 (-0.77 to -0.66)	36.71 (25.02 to 50.44)	-0.71 (-0.76 to -0.66)	0.15 (0.04 to 0.35)	-0.69 (-0.79 to -0.58)
Gabon	26.31 (18.4 to 36.67)	0.03 (0.02 to 0.04)	3.31 (2.21 to 4.64)	-0.01 (-0.03 to 0)	0.01 (0.01 to 0.03)	-0.01 (-0.02 to 0)
Gambia	14.53 (9.67 to 20.6)	0.09 (0.08 to 0.1)	1.96 (1.24 to 2.9)	0.06 (0.04 to 0.08)	0.01 (0 to 0.02)	0.06 (0.05 to 0.08)
Georgia	145.47 (102.34 to 196.93)	0.04 (0.03 to 0.06)	19.71 (13.65 to 26.84)	0 (-0.01 to 0.01)	0.08 (0.03 to 0.18)	-0.07 (-0.12 to -0.02)
Germany	184.79 (130.82 to 254.14)	1.1 (0.43 to 1.76)	26.32 (17.77 to 36.35)	0.91 (0.58 to 1.25)	0.11 (0.03 to 0.24)	0.7 (0.23 to 1.18)
Ghana	14.89 (9.98 to 21.01)	-0.08 (-0.08 to -0.08)	1.99 (1.28 to 2.87)	-0.13 (-0.14 to -0.13)	0.01 (0 to 0.02)	-0.13 (-0.13 to -0.12)
Greece	222.1 (157.62 to 308.7)	0.06 (0.05 to 0.07)	31.06 (21.18 to 42.8)	-0.02 (-0.05 to 0.01)	0.13 (0.03 to 0.29)	-0.12 (-0.27 to 0.04)
Greenland	436.28 (313.62 to 579.37)	0.2 (0.13 to 0.27)	46.11 (32.28 to 62.4)	0.2 (0.15 to 0.24)	0.18 (0.05 to 0.43)	0.18 (-0.07 to 0.43)
Grenada	21.25 (14.63 to 29.77)	-0.01 (-0.04 to 0.03)	2.76 (1.82 to 3.94)	-0.04 (-0.07 to -0.01)	0.01 (0 to 0.02)	-0.04 (-0.07 to -0.01)
Guam	0.17 (0.04 to 0.37)	0 (0 to 0)	0.03 (0.01 to 0.07)	0.01 (0.01 to 0.01)	0 (0 to 0)	0.01 (0.01 to 0.01)
Guatemala	183.09 (130.74 to 251.78)	-0.01 (-0.02 to 0)	20.18 (14.05 to 28.15)	-0.03 (-0.03 to -0.02)	0.08 (0.03 to 0.18)	-0.01 (-0.03 to 0)
Guinea	14.16 (9.54 to 20.03)	0.04	1.89 (1.2 to 2.74)	-0.01	0.01 (0 to 0.02)	0

© 2025 Wang R, et al. *JAMA Dermatol*.

		(0.03 to 0.05)		(-0.02 to 0.01)		(-0.02 to 0.01)
Guinea-Bissau	15.23 (10.2 to 21.49)	-0.09 (-0.1 to -0.08)	1.87 (1.18 to 2.64)	-0.13 (-0.13 to -0.12)	0.01 (0 to 0.02)	-0.13 (-0.13 to -0.12)
Guyana	22.55 (15.34 to 31.92)	-0.06 (-0.07 to -0.04)	2.8 (1.83 to 3.96)	-0.13 (-0.15 to -0.11)	0.01 (0 to 0.02)	-0.13 (-0.15 to -0.12)
Haiti	23.51 (16.17 to 33.15)	0.12 (0.11 to 0.13)	2.91 (1.9 to 4.15)	0.08 (0.08 to 0.09)	0.01 (0.01 to 0.03)	0.08 (0.08 to 0.09)
Honduras	183.75 (131.5 to 250.02)	0 (0 to 0.01)	20.3 (14.15 to 27.5)	-0.03 (-0.03 to -0.02)	0.08 (0.03 to 0.18)	-0.02 (-0.04 to 0)
Hungary	182.04 (128.13 to 247.65)	0 (-0.03 to 0.03)	23.51 (15.75 to 32.21)	-0.03 (-0.07 to 0.01)	0.1 (0.03 to 0.23)	0 (-0.1 to 0.1)
Iceland	223.51 (157.37 to 304.1)	0.05 (0.04 to 0.06)	32.52 (22.3 to 44.58)	0.03 (-0.01 to 0.07)	0.13 (0.03 to 0.32)	0.06 (-0.13 to 0.25)
India	7.99 (5.36 to 11.35)	0.31 (0.19 to 0.43)	0.98 (0.63 to 1.44)	0.3 (0.18 to 0.41)	0 (0 to 0.01)	0.3 (0.18 to 0.41)
Indonesia	6.65 (4.32 to 9.59)	0.04 (0.03 to 0.06)	0.88 (0.55 to 1.3)	-0.01 (-0.01 to 0)	0 (0 to 0.01)	-0.01 (-0.01 to 0)
Iran (Islamic Republic of)	44.56 (31.3 to 61.65)	-0.89 (-1.11 to -0.66)	6.52 (4.42 to 9.02)	-0.73 (-0.92 to -0.54)	0.03 (0.01 to 0.06)	-0.73 (-0.92 to -0.54)
Iraq	24.97 (21.03 to 29.27)	-0.16 (-0.38 to 0.06)	3.62 (2.72 to 4.68)	-0.15 (-0.28 to -0.03)	0.02 (0.01 to 0.03)	-0.14 (-0.25 to -0.04)
Ireland	312.8 (222.56 to 425.11)	-1.53 (-1.66 to -1.4)	40.1 (27.35 to 54.62)	-1.39 (-1.54 to -1.24)	0.16 (0.04 to 0.37)	-1.34 (-1.58 to -1.09)
Israel	225.48 (158.25 to 309.53)	0.07 (0.04 to 0.09)	31.42 (21.53 to 43.07)	-0.05 (-0.1 to -0.01)	0.13 (0.03 to 0.3)	-0.1 (-0.24 to 0.04)
Italy	265.12 (187.03 to 368.35)	0.34	36.42 (24.85 to 50.18)	0.17	0.15 (0.06 to 0.3)	0.18

© 2025 Wang R, et al. *JAMA Dermatol*.

		(0.29 to 0.38)		(0.09 to 0.26)		(0.04 to 0.32)
Jamaica	26.06 (17.97 to 37.02)	-0.3<br>(-0.38 to -0.23)	4.02 (2.66 to 5.67)	-0.34<br>(-0.41 to -0.27)	0.02 (0.01 to 0.04)	-0.34<br>(-0.4 to -0.28)
Japan	25.85 (17.78 to 36.39)	1.17<br>(1.14 to 1.21)	5.41 (3.57 to 7.62)	1.02<br>(0.96 to 1.07)	0.02 (0.01 to 0.05)	1.02<br>(0.96 to 1.07)
Jordan	36.28 (25.23 to 50.27)	-0.43<br>(-0.55 to -0.31)	5.41 (3.61 to 7.46)	-0.33<br>(-0.48 to -0.18)	0.02 (0.01 to 0.05)	-0.36<br>(-0.52 to -0.19)
Kazakhstan	147.24 (105.55 to 200.7)	0.05<br>(0.04 to 0.06)	18.98 (13.05 to 25.75)	0.02<br>(0.01 to 0.02)	0.08 (0.03 to 0.17)	0.03<br>(-0.03 to 0.1)
Kenya	18.66 (12.74 to 26.12)	-0.25<br>(-0.27 to -0.24)	2.49 (1.64 to 3.55)	-0.26<br>(-0.27 to -0.24)	0.01 (0 to 0.02)	-0.26<br>(-0.27 to -0.24)
Kiribati	0.17 (0.04 to 0.37)	0.02<br>(0.02 to 0.02)	0.02 (0.01 to 0.05)	0<br>(-0.01 to 0.01)	0 (0 to 0)	0<br>(-0.01 to 0.01)
Kuwait	26.47 (18.45 to 37.08)	-0.05<br>(-0.12 to 0.01)	4.65 (3.09 to 6.52)	-0.06<br>(-0.14 to 0.02)	0.02 (0.01 to 0.04)	-0.07<br>(-0.15 to 0.01)
Kyrgyzstan	144.4 (102.47 to 196.7)	-0.19<br>(-0.22 to -0.15)	20.06 (13.81 to 27.63)	-0.16<br>(-0.18 to -0.15)	0.09 (0.03 to 0.18)	-0.15<br>(-0.36 to 0.06)
Lao People's Democratic Republic	6.37 (4.07 to 9.13)	0.01<br>(0 to 0.02)	0.81 (0.5 to 1.22)	-0.03<br>(-0.05 to -0.02)	0 (0 to 0.01)	-0.03<br>(-0.05 to -0.02)
Latvia	120.07 (85.02 to 166.14)	0.18<br>(0.16 to 0.19)	17.23 (11.78 to 24.01)	0.13<br>(0.11 to 0.14)	0.08 (0.03 to 0.15)	0.12<br>(0.09 to 0.16)
Lebanon	48.78 (34.48 to 66.96)	-0.04<br>(-0.27 to 0.18)	7.38 (4.96 to 10.29)	-0.06<br>(-0.21 to 0.1)	0.03 (0.01 to 0.06)	-0.05<br>(-0.2 to 0.1)
Lesotho	79.46 (56.21 to 110.03)	0.01<br>(0 to 0.02)	8.57 (6.01 to 11.69)	-0.02<br>(-0.03 to 0)	0.04 (0.02 to 0.07)	-0.02<br>(-0.03 to 0)
Liberia	15.58 (10.39 to 21.83)	-0.06	2.1 (1.35 to 3.03)	-0.08	0.01 (0 to 0.02)	-0.08

© 2025 Wang R, et al. *JAMA Dermatol*.

		(-0.08 to -0.04)		(-0.11 to -0.06)		(-0.11 to -0.06)
Libya	22.09 (15 to 31.8)	-0.03 (-0.07 to 0.01)	3.6 (2.35 to 5.17)	-0.08 (-0.1 to -0.07)	0.02 (0.01 to 0.03)	-0.08 (-0.1 to -0.06)
Lithuania	151.08 (110.17 to 196.25)	-0.16 (-0.29 to -0.02)	21.51 (14.88 to 29.08)	-0.14 (-0.21 to -0.07)	0.09 (0.03 to 0.21)	-0.14 (-0.32 to 0.04)
Luxembourg	223.67 (159.69 to 307.76)	0.18 (0.15 to 0.21)	30.65 (21.04 to 41.52)	0.07 (0.04 to 0.1)	0.13 (0.03 to 0.27)	0.04 (-0.09 to 0.17)
Madagascar	20.16 (13.94 to 28.37)	-0.09 (-0.1 to -0.08)	2.66 (1.78 to 3.77)	-0.13 (-0.13 to -0.12)	0.01 (0 to 0.02)	-0.13 (-0.14 to -0.13)
Malawi	15.94 (10.63 to 22.5)	-0.1 (-0.14 to -0.06)	2.04 (1.32 to 2.97)	-0.13 (-0.15 to -0.11)	0.01 (0 to 0.02)	-0.13 (-0.15 to -0.11)
Malaysia	13.44 (9.28 to 18.82)	-1.13 (-1.21 to -1.05)	1.87 (1.22 to 2.63)	-1.06 (-1.12 to -1)	0.01 (0 to 0.02)	-1.06 (-1.12 to -1)
Maldives	6.42 (4.15 to 9.33)	-0.09 (-0.11 to -0.08)	0.92 (0.57 to 1.38)	-0.13 (-0.14 to -0.12)	0 (0 to 0.01)	-0.12 (-0.14 to -0.1)
Mali	20.91 (14.49 to 29.43)	0.03 (0.03 to 0.03)	2.71 (1.79 to 3.83)	0 (-0.01 to 0.01)	0.01 (0 to 0.02)	0 (0 to 0)
Malta	241.96 (170.37 to 338.21)	-0.63 (-0.73 to -0.53)	33.44 (22.89 to 45.94)	-0.59 (-0.66 to -0.52)	0.14 (0.04 to 0.33)	-0.56 (-0.8 to -0.31)
Marshall Islands	0.17 (0.04 to 0.37)	-0.02 (-0.02 to -0.02)	0.02 (0 to 0.05)	-0.05 (-0.05 to -0.05)	0 (0 to 0)	-0.04 (-0.05 to -0.04)
Mauritania	15.55 (10.55 to 21.96)	0.12 (0.11 to 0.13)	2.19 (1.43 to 3.2)	0.09 (0.08 to 0.1)	0.01 (0 to 0.02)	0.09 (0.08 to 0.11)
Mauritius	6.26 (4.02 to 9.23)	0.05 (0.03 to 0.07)	0.93 (0.57 to 1.42)	0.01 (0 to 0.02)	0 (0 to 0.01)	0.01 (0 to 0.02)
Mexico	184.82 (131.12 to 253.31)	-0.02	21.91 (15.18 to 29.87)	-0.04	0.09 (0.04 to 0.18)	-0.01

© 2025 Wang R, et al. *JAMA Dermatol*.

		(-0.03 to -0.02)		(-0.05 to -0.03)		(-0.04 to 0.02)
Micronesia (Federated States of)	0.17 (0.04 to 0.37)	0<br>(0 to 0)	0.02 (0 to 0.05)	-0.03<br>(-0.04 to -0.03)	0 (0 to 0)	-0.03<br>(-0.04 to -0.03)
Monaco	226.14 (159.33 to 314.67)	0.08<br>(0.06 to 0.11)	30.61 (20.73 to 42.11)	0.05<br>(0.03 to 0.06)	0.13 (0.04 to 0.29)	0.02<br>(-0.16 to 0.19)
Mongolia	150.97 (108.18 to 206.32)	0.02<br>(0.01 to 0.02)	18.27 (12.63 to 25.05)	0<br>(-0.01 to 0)	0.08 (0.03 to 0.16)	0<br>(-0.06 to 0.06)
Montenegro	177.41 (124.98 to 241.48)	0.01<br>(-0.01 to 0.03)	23.27 (15.59 to 32.01)	-0.01<br>(-0.03 to 0)	0.1 (0.03 to 0.22)	0<br>(-0.03 to 0.03)
Morocco	19.38 (13.25 to 27.07)	-0.02<br>(-0.05 to 0.01)	2.81 (1.85 to 4.03)	-0.06<br>(-0.08 to -0.03)	0.01 (0 to 0.02)	-0.05<br>(-0.11 to 0)
Mozambique	19.38 (13.28 to 27.58)	-0.14<br>(-0.14 to -0.13)	2.43 (1.57 to 3.46)	-0.17<br>(-0.18 to -0.15)	0.01 (0 to 0.02)	-0.16<br>(-0.17 to -0.15)
Myanmar	6.25 (3.99 to 9.06)	-0.02<br>(-0.03 to 0)	0.86 (0.52 to 1.3)	-0.04<br>(-0.05 to -0.04)	0 (0 to 0.01)	-0.05<br>(-0.05 to -0.04)
Namibia	84.86 (60.34 to 115.77)	-0.01<br>(-0.11 to 0.09)	9.72 (6.65 to 13.36)	-0.03<br>(-0.12 to 0.07)	0.04 (0.02 to 0.08)	-0.04<br>(-0.13 to 0.06)
Nauru	0.17 (0.04 to 0.37)	0.03<br>(0.03 to 0.04)	0.02 (0 to 0.05)	0.05<br>(0.04 to 0.05)	0 (0 to 0)	0.05<br>(0.04 to 0.05)
Nepal	0.2 (0.04 to 0.46)	-0.03<br>(-0.04 to -0.02)	0.03 (0.01 to 0.06)	-0.07<br>(-0.08 to -0.06)	0 (0 to 0)	-0.07<br>(-0.07 to -0.06)
Netherlands	157.85 (111.86 to 216.39)	0.77<br>(0.75 to 0.8)	23.15 (15.7 to 31.64)	0.62<br>(0.58 to 0.65)	0.1 (0.03 to 0.21)	0.56<br>(0.51 to 0.62)
New Zealand	171.66 (122.02 to 231.24)	0.08<br>(0.02 to 0.13)	24.15 (16.59 to 33.26)	-0.02<br>(-0.07 to 0.03)	0.1 (0.03 to 0.23)	0.05<br>(-0.16 to 0.27)
Nicaragua	174.11 (121.81 to 241.02)	-0.02	22.23 (15.25 to 30.69)	-0.05	0.09 (0.03 to 0.21)	-0.04

© 2025 Wang R, et al. *JAMA Dermatol*.

		(-0.03 to -0.01)		(-0.05 to -0.04)		(-0.06 to -0.02)
Niger	15.53 (10.41 to 21.85)	0.01<br>(0 to 0.03)	2.05 (1.32 to 2.93)	-0.03<br>(-0.04 to -0.01)	0.01 (0 to 0.02)	-0.03<br>(-0.05 to -0.01)
Nigeria	13.41 (9 to 18.93)	0.07<br>(0.06 to 0.09)	1.83 (1.19 to 2.65)	0.04<br>(0.02 to 0.06)	0.01 (0 to 0.02)	0.04<br>(0.02 to 0.06)
Niue	0.17 (0.04 to 0.37)	0<br>(0 to 0)	0.03 (0.01 to 0.06)	0<br>(-0.01 to 0)	0 (0 to 0)	0<br>(-0.01 to 0)
North Macedonia	184.49 (132.21 to 250.41)	-0.01<br>(-0.02 to 0)	22.19 (15.38 to 30.32)	-0.01<br>(-0.02 to 0)	0.09 (0.03 to 0.2)	-0.01<br>(-0.04 to 0.03)
Northern Mariana Islands	0.17 (0.04 to 0.37)	0<br>(0 to 0)	0.03 (0.01 to 0.06)	0.01<br>(0 to 0.02)	0 (0 to 0)	0.01<br>(0 to 0.01)
Norway	234.97 (167.04 to 322.45)	0.08<br>(0.05 to 0.1)	32.02 (21.92 to 43.7)	0.07<br>(0.02 to 0.11)	0.13 (0.05 to 0.27)	0.1<br>(0.02 to 0.17)
Oman	31.8 (24.44 to 40.23)	-0.02<br>(-0.19 to 0.15)	3.95 (2.82 to 5.2)	-0.05<br>(-0.23 to 0.14)	0.02 (0.01 to 0.04)	-0.04<br>(-0.14 to 0.06)
Pakistan	0.82 (0.49 to 1.26)	-0.63<br>(-0.7 to -0.56)	0.11 (0.06 to 0.17)	-0.65<br>(-0.71 to -0.6)	0 (0 to 0)	-0.65<br>(-0.71 to -0.6)
Palau	0.17 (0.04 to 0.37)	0<br>(-0.01 to 0)	0.02 (0.01 to 0.06)	-0.01<br>(-0.01 to 0)	0 (0 to 0)	-0.01<br>(-0.01 to 0)
Palestine	28.47 (19.85 to 39.98)	0<br>(-0.01 to 0.01)	3.77 (2.54 to 5.27)	-0.02<br>(-0.04 to 0)	0.02 (0.01 to 0.03)	-0.02<br>(-0.04 to 0)
Panama	121.51 (106.17 to 138.55)	0.25<br>(0.2 to 0.3)	17.04 (13.36 to 21.53)	0.16<br>(0.11 to 0.22)	0.07 (0.03 to 0.16)	0.1<br>(-0.01 to 0.2)
Papua New Guinea	0.17 (0.04 to 0.37)	0.02<br>(0.02 to 0.03)	0.02 (0.01 to 0.06)	-0.01<br>(-0.01 to 0)	0 (0 to 0)	0<br>(-0.01 to 0.01)
Paraguay	0.26 (0.07 to 0.54)	-0.98	0.05 (0.01 to 0.1)	-1.02	0 (0 to 0)	-1.04

© 2025 Wang R, et al. *JAMA Dermatol*.

		(-1.13 to -0.82)		(-1.17 to -0.86)		(-1.16 to -0.92)
Peru	65.94 (47.95 to 86.22)	-1.21 (-1.32 to -1.1)	9.84 (6.96 to 13.37)	-1.07 (-1.13 to -1.01)	0.04 (0.02 to 0.09)	-1.09 (-1.16 to -1.02)
Philippines	10.75 (7.14 to 15.2)	-1.34 (-1.41 to -1.27)	1.47 (0.94 to 2.14)	-1.29 (-1.35 to -1.23)	0.01 (0 to 0.01)	-1.29 (-1.35 to -1.23)
Poland	135.97 (95.33 to 189.34)	1.15 (1.11 to 1.2)	19.01 (12.95 to 26.21)	0.96 (0.91 to 1.01)	0.08 (0.03 to 0.16)	0.88 (0.82 to 0.95)
Portugal	134.77 (95.92 to 183.22)	1.07 (1.03 to 1.12)	20.44 (13.62 to 28.23)	0.86 (0.81 to 0.92)	0.09 (0.03 to 0.19)	0.81 (0.67 to 0.95)
Puerto Rico	21.45 (14.81 to 30.58)	-0.06 (-0.07 to -0.05)	3.51 (2.31 to 4.99)	-0.08 (-0.08 to -0.07)	0.02 (0.01 to 0.03)	-0.08 (-0.09 to -0.08)
Qatar	26.12 (16.14 to 38.95)	-0.03 (-0.07 to 0.02)	3.56 (2.21 to 5.28)	-0.05 (-0.1 to 0)	0.02 (0.01 to 0.03)	-0.06 (-0.15 to 0.02)
Republic of Korea	33.52 (29.52 to 36.99)	1.97 (1.87 to 2.07)	6.12 (4.71 to 7.78)	1.69 (1.63 to 1.75)	0.03 (0.01 to 0.06)	1.69 (1.63 to 1.75)
Republic of Moldova	130.37 (92.1 to 180.62)	0.02 (0.01 to 0.03)	17.23 (11.83 to 23.49)	0 (-0.02 to 0.02)	0.07 (0.03 to 0.15)	0 (-0.04 to 0.04)
Romania	137.02 (97.3 to 190.16)	1.35 (1.33 to 1.37)	18.27 (12.42 to 25.33)	1.17 (1.15 to 1.19)	0.08 (0.03 to 0.17)	1.12 (1.07 to 1.17)
Russian Federation	115.78 (82.6 to 158.44)	0.95 (0.91 to 0.99)	15.46 (10.69 to 21.04)	0.81 (0.78 to 0.85)	0.07 (0.03 to 0.13)	0.81 (0.76 to 0.85)
Rwanda	19.25 (13.03 to 27.32)	-0.1 (-0.11 to -0.09)	2.46 (1.61 to 3.53)	-0.12 (-0.13 to -0.11)	0.01 (0 to 0.02)	-0.12 (-0.13 to -0.11)
Saint Kitts and Nevis	21.79 (15.14 to 30.5)	0.02 (-0.01 to 0.05)	2.76 (1.83 to 3.92)	0.02 (-0.01 to 0.05)	0.01 (0 to 0.02)	0.02 (-0.01 to 0.04)
Saint Lucia	22.04 (15.13 to 30.45)	0.15	3.23 (2.17 to 4.57)	0.08	0.01 (0.01 to 0.03)	0.09

© 2025 Wang R, et al. *JAMA Dermatol*.

		(0.14 to 0.16)		(0.07 to 0.1)		(0.07 to 0.11)
Saint Vincent and the Grenadines	23.26 (16.01 to 32.52)	0.32 (0.3 to 0.34)	3.29 (2.17 to 4.71)	0.25 (0.23 to 0.27)	0.01 (0.01 to 0.03)	0.26 (0.23 to 0.29)
Samoa	0.17 (0.04 to 0.37)	-0.66 (-0.74 to -0.58)	0.03 (0.01 to 0.06)	-0.68 (-0.74 to -0.61)	0 (0 to 0)	-0.67 (-0.77 to -0.57)
San Marino	221.26 (155.57 to 304.79)	0.04 (0.03 to 0.05)	33.94 (23.02 to 46.55)	0.04 (0.01 to 0.07)	0.14 (0.03 to 0.34)	0.07 (-0.08 to 0.22)
Sao Tome and Principe	15.08 (10.13 to 21.47)	0.05 (0.02 to 0.07)	2.1 (1.36 to 3.06)	0.02 (0 to 0.04)	0.01 (0 to 0.02)	0.02 (-0.01 to 0.04)
Saudi Arabia	27.64 (23.77 to 31.63)	0.21 (0 to 0.43)	4.16 (3.17 to 5.43)	0.25 (0.19 to 0.31)	0.02 (0.01 to 0.04)	0.26 (0.2 to 0.31)
Senegal	25.01 (17.27 to 34.91)	-0.02 (-0.03 to 0)	3.28 (2.16 to 4.61)	-0.04 (-0.05 to -0.03)	0.01 (0.01 to 0.03)	-0.04 (-0.06 to -0.03)
Serbia	166.61 (118.66 to 227.83)	0.83 (0.8 to 0.87)	20.99 (14.24 to 29.01)	0.69 (0.64 to 0.73)	0.09 (0.03 to 0.19)	0.62 (0.47 to 0.77)
Seychelles	6.41 (3.32 to 10.4)	0.25 (0.23 to 0.26)	0.93 (0.45 to 1.53)	0.16 (0.13 to 0.18)	0 (0 to 0.01)	0.16 (0.14 to 0.18)
Sierra Leone	15.47 (10.41 to 21.92)	-0.04 (-0.06 to -0.02)	2.04 (1.29 to 2.91)	-0.06 (-0.09 to -0.04)	0.01 (0 to 0.02)	-0.06 (-0.09 to -0.03)
Singapore	39.7 (27.47 to 55.31)	-1.25 (-1.44 to -1.06)	7.33 (4.84 to 10.27)	-1.18 (-1.39 to -0.96)	0.03 (0.01 to 0.06)	-1.08 (-1.25 to -0.9)
Slovakia	192.82 (135.86 to 267.14)	-0.37 (-0.53 to -0.22)	25.44 (17.11 to 35.09)	-0.33 (-0.39 to -0.26)	0.1 (0.03 to 0.25)	-0.31 (-0.5 to -0.13)
Slovenia	255.31 (185.34 to 338.65)	1.59 (1.45 to 1.74)	34.98 (24.62 to 46.95)	1.21 (1.16 to 1.26)	0.14 (0.03 to 0.34)	1.1 (0.88 to 1.32)
Solomon Islands	0.17 (0.04 to 0.37)	-0.01	0.02 (0 to 0.05)	-0.03	0 (0 to 0)	-0.03

© 2025 Wang R, et al. *JAMA Dermatol*.

		(-0.02 to 0)		(-0.04 to -0.03)		(-0.04 to -0.02)
Somalia	19.2 (13.19 to 26.98)	-0.19 (-0.2 to -0.17)	2.32 (1.5 to 3.31)	-0.22 (-0.24 to -0.2)	0.01 (0 to 0.02)	-0.22 (-0.24 to -0.2)
South Africa	116.05 (81.96 to 155.95)	0.63 (0.48 to 0.77)	13.55 (9.39 to 18.44)	0.55 (0.42 to 0.68)	0.06 (0.02 to 0.12)	0.54 (0.39 to 0.69)
South Sudan	21.09 (14.4 to 29.95)	-0.08 (-0.09 to -0.08)	2.7 (1.76 to 3.9)	-0.11 (-0.12 to -0.1)	0.01 (0 to 0.02)	-0.11 (-0.12 to -0.11)
Spain	243.1 (172.09 to 336.94)	-0.61 (-0.81 to -0.41)	35.53 (24.26 to 48.67)	-0.62 (-0.75 to -0.48)	0.14 (0.03 to 0.35)	-0.65 (-1.04 to -0.26)
Sri Lanka	5.12 (3.24 to 7.61)	-0.53 (-0.59 to -0.46)	0.78 (0.47 to 1.2)	-0.56 (-0.63 to -0.5)	0 (0 to 0.01)	-0.58 (-0.63 to -0.53)
Sudan	30.55 (21.5 to 42.34)	0.07 (0.06 to 0.08)	4.08 (2.72 to 5.69)	0.05 (0.04 to 0.06)	0.02 (0.01 to 0.04)	0.05 (0.04 to 0.06)
Suriname	21.65 (14.87 to 30.53)	-0.08 (-0.09 to -0.07)	3.18 (2.08 to 4.56)	-0.13 (-0.14 to -0.12)	0.01 (0.01 to 0.03)	-0.13 (-0.14 to -0.12)
Sweden	351.92 (260.87 to 470.23)	1.47 (1.35 to 1.59)	45.51 (31.58 to 62.54)	1.17 (1.05 to 1.29)	0.18 (0.06 to 0.41)	1.17 (0.99 to 1.34)
Switzerland	295.3 (211.86 to 409.15)	-1.28 (-1.49 to -1.07)	41.78 (28.25 to 57.24)	-1.23 (-1.41 to -1.04)	0.17 (0.04 to 0.4)	-1.22 (-1.48 to -0.96)
Syrian Arab Republic	29.61 (20.2 to 41.06)	0.02 (0.01 to 0.03)	4.36 (2.9 to 6.15)	0.02 (0.01 to 0.02)	0.02 (0.01 to 0.04)	0.02 (0.01 to 0.03)
Taiwan (Province of China)	0.3 (0.09 to 0.58)	-12.11 (-13.48 to -10.72)	0.05 (0.02 to 0.11)	-11.81 (-13.32 to -10.27)	0 (0 to 0)	-11.19 (-11.95 to -10.43)
Tajikistan	151.75 (108.28 to 206.77)	0.06 (0.05 to 0.07)	18.9 (12.86 to 25.64)	0.04 (0.01 to 0.06)	0.08 (0.03 to 0.17)	0.03 (-0.02 to 0.07)
Thailand	14.52 (9.9 to 20.23)	-3.06	2.27 (1.51 to 3.25)	-2.76	0.01 (0 to 0.02)	-2.76

© 2025 Wang R, et al. *JAMA Dermatol*.

		(-3.21 to -2.92)		(-2.87 to -2.65)		(-3.05 to -2.47)
Timor-Leste	6.43 (4.18 to 9.35)	0.03<br>(0 to 0.06)	0.88 (0.54 to 1.33)	0<br>(-0.02 to 0.01)	0 (0 to 0.01)	0<br>(-0.02 to 0.01)
Togo	14.65 (9.83 to 20.56)	-0.12<br>(-0.15 to -0.1)	1.95 (1.24 to 2.84)	-0.15<br>(-0.16 to -0.13)	0.01 (0 to 0.02)	-0.14<br>(-0.16 to -0.13)
Tokelau	0.17 (0.04 to 0.37)	0<br>(0 to 0)	0.02 (0.01 to 0.06)	-0.01<br>(-0.01 to 0)	0 (0 to 0)	0<br>(-0.01 to 0)
Tonga	0.17 (0.04 to 0.37)	0<br>(0 to 0.01)	0.03 (0.01 to 0.06)	0<br>(-0.01 to 0)	0 (0 to 0)	0<br>(-0.01 to 0)
Trinidad and Tobago	21.16 (14.57 to 29.77)	0.39<br>(0.36 to 0.42)	3.04 (2.04 to 4.34)	0.31<br>(0.24 to 0.37)	0.01 (0.01 to 0.03)	0.31<br>(0.25 to 0.36)
Tunisia	34.13 (24.02 to 47.77)	-0.31<br>(-0.37 to -0.25)	5.21 (3.56 to 7.26)	-0.29<br>(-0.37 to -0.21)	0.02 (0.01 to 0.05)	-0.29<br>(-0.36 to -0.22)
Turkmenistan	147.37 (104.49 to 196.55)	0.04<br>(0.03 to 0.05)	19.58 (13.56 to 26.32)	0.03<br>(0.02 to 0.03)	0.08 (0.03 to 0.18)	0.01<br>(-0.02 to 0.05)
Tuvalu	0.17 (0.04 to 0.37)	-0.01<br>(-0.01 to -0.01)	0.02 (0 to 0.05)	0<br>(0 to 0)	0 (0 to 0)	0<br>(0 to 0)
Turkey	55.05 (39.33 to 75.89)	-1.37<br>(-1.53 to -1.21)	8.08 (5.47 to 11.13)	-1.2<br>(-1.34 to -1.07)	0.04 (0.01 to 0.07)	-1.22<br>(-1.42 to -1.01)
Uganda	25.59 (18.29 to 34.39)	-0.37<br>(-0.45 to -0.28)	3.27 (2.26 to 4.54)	-0.36<br>(-0.44 to -0.28)	0.01 (0.01 to 0.03)	-0.37<br>(-0.44 to -0.29)
Ukraine	134.73 (97.24 to 183.16)	0.08<br>(0.07 to 0.08)	17.4 (12.11 to 23.72)	0.03<br>(0.03 to 0.04)	0.08 (0.03 to 0.15)	0.04<br>(0.02 to 0.05)
United Arab Emirates	35.13 (24.29 to 49.36)	0.46<br>(0.44 to 0.49)	4.41 (2.91 to 6.21)	0.52<br>(0.49 to 0.55)	0.02 (0.01 to 0.04)	0.52<br>(0.49 to 0.55)
United Kingdom	268.42 (190.93 to 366.42)	-0.08	34.77 (23.95 to 47.43)	-0.08	0.14 (0.06 to 0.28)	-0.09

© 2025 Wang R, et al. *JAMA Dermatol*.

		(-0.17 to 0.01)		(-0.14 to -0.02)		(-0.16 to -0.01)
United Republic of Tanzania	20.1 (13.76 to 27.76)	-0.01<br>(-0.02 to -0.01)	2.68 (1.76 to 3.82)	-0.04<br>(-0.05 to -0.04)	0.01 (0 to 0.02)	-0.05<br>(-0.06 to -0.04)
United States of America	3452.24 (2971.67 to 3949.03)	2.91<br>(2.81 to 3)	343.35 (285.91 to 413.48)	2.74<br>(2.64 to 2.83)	1.35 (0.62 to 2.62)	2.69<br>(2.59 to 2.79)
United States Virgin Islands	22.12 (15.2 to 31.02)	0.05<br>(0.02 to 0.09)	3.07 (2.03 to 4.36)	0.03<br>(0 to 0.06)	0.01 (0.01 to 0.03)	0.03<br>(0 to 0.06)
Uruguay	162.12 (114.58 to 223.98)	-0.11<br>(-0.43 to 0.2)	22.96 (15.57 to 31.67)	0.03<br>(-0.23 to 0.28)	0.1 (0.03 to 0.21)	0<br>(-0.33 to 0.33)
Uzbekistan	153.71 (110.72 to 203.81)	0.04<br>(0.03 to 0.04)	17.62 (12.24 to 23.94)	0.03<br>(0.03 to 0.04)	0.08 (0.03 to 0.15)	0.03<br>(-0.03 to 0.1)
Vanuatu	0.17 (0.04 to 0.37)	0<br>(0 to 0)	0.02 (0.01 to 0.05)	-0.02<br>(-0.03 to -0.02)	0 (0 to 0)	-0.02<br>(-0.02 to -0.02)
Venezuela (Bolivarian Republic of)	176.48 (124.23 to 242.85)	-0.04<br>(-0.07 to 0)	21.35 (14.51 to 29.66)	-0.04<br>(-0.06 to -0.02)	0.09 (0.03 to 0.19)	-0.02<br>(-0.05 to 0)
Viet Nam	8.34 (5.47 to 12.14)	-1.83<br>(-1.9 to -1.75)	1.2 (0.76 to 1.78)	-1.79<br>(-1.81 to -1.77)	0.01 (0 to 0.01)	-1.8<br>(-1.84 to -1.77)
Yemen	29.27 (20.46 to 40.62)	0.17<br>(0.16 to 0.19)	3.88 (2.6 to 5.48)	0.15<br>(0.14 to 0.16)	0.02 (0.01 to 0.03)	0.15<br>(0.14 to 0.16)
Zambia	20.13 (13.57 to 28.32)	-0.18<br>(-0.19 to -0.17)	2.53 (1.64 to 3.63)	-0.2<br>(-0.21 to -0.2)	0.01 (0 to 0.02)	-0.2<br>(-0.2 to -0.19)
Zimbabwe	64.44 (45.64 to 86.75)	-0.18<br>(-0.63 to 0.26)	7.37 (5.08 to 10.15)	-0.2<br>(-0.6 to 0.2)	0.03 (0.01 to 0.06)	-0.21<br>(-0.63 to 0.21)

Abbreviations: UI, uncertainty interval; ASR, age-standerised rate per 100,000; CI, confidence interval; DALYs, disability-adjusted life-year.

© 2025 Wang R, et al. *JAMA Dermatol*.

eTable 7. Decomposition of change in incidence globally and by SDI quintile, 1990 to 2021

Cause	Location	Overall difference	Aging	Population growth	Epidemiological changes
Cutaneous melanoma	Global	109278.76	2591.96(2.37%)	74719.2(68.37%)	31967.6(29.25%)
	High SDI	77691.38	1135.28(1.46%)	44418.1(57.17%)	32138(41.37%)
	High-middle SDI	22241.88	393.81(1.77%)	12156.36(54.66%)	9691.71(43.57%)
	Middle SDI	6976.85	209.32(3%)	4497.58(64.46%)	2269.95(32.54%)
	Low-middle SDI	1655.37	21.62(1.31%)	1172.64(70.84%)	461.11(27.86%)
	Low SDI	582.24	6.58(1.13%)	527.25(90.56%)	48.41(8.31%)
Squamous cell carcinoma	Global	1114737.96	42523.87 (3.81%)	661006.34 (59.3%)	411207.75 (36.89%)
	High SDI	1018649.18	31761.99 (3.12%)	488687.63 (47.97%)	498199.57 (48.91%)
	High-middle SDI	51498.62	1193.13 (2.32%)	27361.37 (53.13%)	22944.11 (44.55%)
	Middle SDI	43051.89	643.64 (1.5%)	21029.75 (48.85%)	21378.5 (49.66%)
	Low-middle SDI	1319.23	26.48 (2.01%)	1391.46 (105.48%)	-98.71 (-7.48%)
	Low SDI	147.46	3 (2.03%)	125.04 (84.79%)	19.43 (13.17%)
Basal cell carcinoma	Global	2087861.88	46632.55 (2.23%)	1296821.31 (62.11%)	744408.02 (35.65%)
	High SDI	1635842.58	25024.35 (1.53%)	809379.85 (49.48%)	801438.38 (48.99%)
	High-middle SDI	216500.01	3403.36 (1.57%)	138156.37 (63.81%)	74940.29 (34.61%)
	Middle SDI	218294.81	2464.78 (1.13%)	136210.73 (62.4%)	79619.29 (36.47%)
	Low-middle SDI	14034.12	424.69 (3.03%)	19529.1(139.15%)	-5919.67 (-42.18%)
	Low SDI	2781.68	43.7 (1.57%)	2906.08 (104.47%)	-168.11 (-6.04%)

Abbreviations: SDI, sociodemographic index.

eTable 8. Decomposition of change in DALYs globally and by SDI quintile, 1990 to 2021

Cause	Location	Overall difference	Aging	Population growth	Epidemiological changes
Cutaneous melanoma	Global	380461.21	8566.19 (2.25%)	387566.78 (101.87%)	-15671.76 (-4.12%)
	High SDI	189325.26	2944.71 (1.56%)	173130.11 (91.45%)	13250.44 (7%)
	High-middle SDI	99038.41	1724.92 (1.74%)	85443.94 (86.27%)	11869.55 (11.98%)
	Middle SDI	61283.72	1382.4 (2.26%)	54874.08 (89.54%)	5027.23 (8.2%)
	Low-middle SDI	21214.79	88.78 (0.42%)	18400.4 (86.73%)	2725.61 (12.85%)
	Low SDI	8993.62	-31.13 (-0.35%)	9692.32 (107.77%)	-667.56 (-7.42%)
Squamous cell carcinoma	Global	450154.27	28459.89 (6.32%)	378107.62 (84%)	43586.76 (9.68%)
	High SDI	126864.57	13196.3 (10.4%)	98844.05 (77.91%)	14824.22 (11.69%)
	High-middle SDI	103823.9	9665.08 (9.31%)	93935.61 (90.48%)	223.22 (0.21%)
	Middle SDI	160372.54	7352.1 (4.58%)	138284 (86.23%)	14736.44 (9.19%)
	Low-middle SDI	49168.97	1521.83 (3.1%)	35213.51 (71.62%)	12433.64 (25.29%)
	Low SDI	9728.58	307 (3.16%)	6714.79 (69.02%)	2706.8 (27.82%)
Basal cell carcinoma	Global	869.58	19.24 (2.21%)	579.59 (66.65%)	270.75 (31.14%)
	High SDI	647.35	9.78 (1.51%)	343.32 (53.04%)	294.25 (45.45%)
	High-middle SDI	108.52	1.54 (1.42%)	76.44 (70.44%)	30.54 (28.14%)
	Middle SDI	104.2	1.14 (1.09%)	65.59 (62.94%)	37.47 (35.96%)
	Low-middle SDI	7.77	0.21 (2.72%)	9.87 (127.06%)	-2.31 (-29.78%)
	Low SDI	1.53	0.03 (1.91%)	1.61 (105.41%)	-0.11 (-7.31%)

Abbreviations: DALY, disability-adjusted life years; SDI, sociodemographic index.

© 2025 Wang R, et al. *JAMA Dermatol.*

eTable 9. Frontier analysis based on SDI and DALYs of cutaneous melanoma in 204 countries and territories.

Location	SDI	DALYs rate in 2021	Frontier DALYs	Effective difference
Afghanistan	0.337199998	44.27(14.46 to 91.99)	2.6	41.67
Albania	0.706849791	84.24(48.68 to 128.31)	1.76	82.48
Algeria	0.659500924	11.42(5.97 to 18.33)	2.39	9.03
American Samoa	0.723727533	55.97(32.12 to 85.24)	1.78	54.19
Andorra	0.869444113	134.87(75.88 to 213.26)	1.74	133.13
Angola	0.453721949	57.34(32.66 to 91.44)	2.39	54.95
Antigua and Barbuda	0.749886887	110.21(97.11 to 126.14)	2.01	108.2
Argentina	0.723122973	112.42(92.75 to 135.07)	1.85	110.57
Armenia	0.701833194	47.45(39.17 to 55.79)	2.4	45.05
Australia	0.844252814	491.63(396.84 to 596.61)	1.87	489.76
Austria	0.853837004	244.58(198.28 to 291.19)	2.02	242.56
Azerbaijan	0.694851274	51.37(30.95 to 79.63)	2.41	48.96
Bahamas	0.805020668	82.02(65.93 to 101.68)	2.31	79.71
Bahrain	0.753043204	19.16(9.47 to 30.89)	1.95	17.21
Bangladesh	0.492420885	17.55(8.62 to 32.46)	2.39	15.16
Barbados	0.746748764	55.58(43.7 to 69.03)	1.81	53.77
Belarus	0.784484711	212.87(165.08 to 269.87)	1.93	210.94
Belgium	0.853654016	189.79(152.72 to 230.05)	1.76	188.03
Belize	0.610229002	30.98(25.87 to 36.12)	2.4	28.58
Benin	0.373486574	23.27(8.62 to 37.96)	2.51	20.76
Bermuda	0.821365422	210.01(161.12 to 271.57)	1.77	208.24
Bhutan	0.473062378	19.25(10.3 to 35.97)	2.4	16.85
Bolivia (Plurinational State of)	0.599010799	145.23(80.8 to 245.62)	2.4	142.83
Bosnia and Herzegovina	0.723077893	165.76(86.71 to 227.33)	1.86	163.9
Botswana	0.642721629	133.01(77.59 to 221.64)	2.39	130.62
Brazil	0.653043887	102.04(90.33 to 111.53)	2.39	99.65
Brunei Darussalam	0.810234367	45.3(27.83 to 65.98)	1.9	43.4
Bulgaria	0.768150939	162.72(131.88 to 196.13)	1.79	160.93
Burkina Faso	0.285118402	28.44(11.51 to 45.81)	8.7	19.74
Burundi	0.289374365	84.42(49.7 to 125.6)	7.26	77.16
Cabo Verde	0.533534539	37.31(5.69 to 69.19)	2.39	34.92
Cambodia	0.473621491	31.11(18.27 to 48.41)	2.4	28.71
Cameroon	0.479691223	28.19(10.89 to 46.17)	2.39	25.8
Canada	0.87317068	199.54(161.59 to 239.1)	2.34	197.2
Central African Republic	0.30916769	53.48(32.6 to 84.64)	7.56	45.92
Chad	0.240436019	23.25(11.16 to 38.02)	16.79	6.46
Chile	0.771514716	100.83(82.94 to 120.84)	2.16	98.67
China	0.72162976	26.37(13.63 to 34.87)	2.03	24.34

© 2025 Wang R, et al. *JAMA Dermatol.*

Colombia	0.655442913	88.86(69.58 to 110.84)	2.4	86.46
Comoros	0.475978688	78.39(41.36 to 122.91)	2.4	75.99
Congo	0.583075236	59.8(36.32 to 91.07)	2.4	57.4
Cook Islands	0.779109955	30.36(18.59 to 47.47)	1.75	28.61
Costa Rica	0.700340477	123.58(97.71 to 151.13)	2.39	121.19
Croatia	0.425941883	305.68(245.68 to 374.33)	1.84	303.84
Cuba	0.798341027	64.65(50.64 to 79.44)	2.4	62.25
Cyprus	0.668729864	141.3(72.15 to 213.87)	1.77	139.53
Czechia	0.835630545	285.15(230.67 to 348.45)	1.96	283.19
Côte d'Ivoire	0.828450433	39.79(22.85 to 63.45)	2.4	37.39
Democratic People's Republic of Korea	0.569854634	28.71(17.45 to 46.04)	2.39	26.32
Democratic Republic of the Congo	0.383179849	54.33(30.3 to 88.05)	2.42	51.91
Denmark	0.896424204	321.32(262.42 to 386.48)	1.79	319.53
Djibouti	0.487958371	80.86(41.3 to 131.67)	2.39	78.47
Dominica	0.746967185	41.69(24.01 to 62.11)	1.83	39.86
Dominican Republic	0.619388201	18.27(11 to 29.14)	2.4	15.87
Ecuador	0.661017053	121.93(92.01 to 156.58)	2.4	119.53
Egypt	0.606787094	5.52(3.31 to 7.97)	2.39	3.13
El Salvador	0.563775188	20.64(10.85 to 28.84)	2.39	18.25
Equatorial Guinea	0.657857456	55.64(28.13 to 91.82)	2.39	53.25
Eritrea	0.403863943	98.22(58.63 to 150.38)	2.4	95.82
Estonia	0.844917787	231.34(183.31 to 281.69)	1.81	229.53
Eswatini	0.585459713	162.92(100.2 to 275.17)	2.4	160.52
Ethiopia	0.358823295	46.74(29.36 to 64.13)	2.61	44.13
Fiji	0.675051631	42.19(22.16 to 87)	2.4	39.79
Finland	0.859831368	243.18(194.28 to 295.69)	1.82	241.36
France	0.838364875	169.3(136.97 to 204.93)	2.04	167.26
Gabon	0.634691393	63.36(35.7 to 98.41)	2.39	60.97
Gambia	0.40971416	22.5(13.23 to 35.05)	2.39	20.11
Georgia	0.732473604	169.21(142.07 to 198.54)	1.97	167.24
Germany	0.902957091	220.73(179.93 to 265.62)	1.74	218.99
Ghana	0.56493039	2.72(1.38 to 4.74)	2.4	0.32
Greece	0.791854408	134.82(116.12 to 152.68)	1.94	132.88
Greenland	0.826210336	96.84(54.7 to 148.99)	1.85	94.99
Grenada	0.668993028	70.02(56.08 to 86.42)	2.4	67.62
Guam	0.803982203	8.07(5.07 to 12.74)	1.91	6.16
Guatemala	0.539972424	36.62(30.19 to 43.45)	2.4	34.22
Guinea	0.336401293	87.08(50.91 to 136)	2.62	84.46
Guinea-Bissau	0.353109621	30.42(13.61 to 51.49)	2.6	27.82
Guyana	0.650812335	33.38(24.52 to 44.09)	2.4	30.98
Haiti	0.448278285	69.21(36.61 to 120.83)	2.4	66.81
Honduras	0.513037248	29.46(18.07 to 43)	2.39	27.07

Hungary	0.790754768	208.49(167.33 to 254.91)	1.84	206.65
Iceland	0.87636168	219.05(172.2 to 271.15)	2	217.05
India	0.575401649	18.93(12.3 to 24.78)	2.4	16.53
Indonesia	0.656868336	19.92(12.94 to 26.55)	2.39	17.53
Iran (Islamic Republic of)	0.697207398	50.79(26.31 to 66.13)	2.39	48.4
Iraq	0.662626231	17.96(10.87 to 29.16)	2.39	15.57
Ireland	0.87375385	202.76(160.94 to 245.59)	1.99	200.77
Israel	0.809011652	235.58(188.08 to 285.46)	1.85	233.73
Italy	0.805773534	178.63(156.1 to 196.86)	1.95	176.68
Jamaica	0.683263064	53.22(38.81 to 70.39)	2.39	50.83
Japan	0.871241813	25.14(21.47 to 27.79)	1.85	23.29
Jordan	0.725307227	9.73(4.94 to 15.3)	2.02	7.71
Kazakhstan	0.725144495	92.59(76.58 to 109.57)	1.9	90.69
Kenya	0.523768077	42.51(25.84 to 60.16)	2.4	40.11
Kiribati	0.527186583	7.85(2.76 to 13.41)	2.39	5.46
Kuwait	0.846651055	7.5(5.52 to 9.98)	1.91	5.59
Kyrgyzstan	0.603979328	60.11(45.65 to 77.08)	2.39	57.72
Lao People's Democratic Republic	0.489136091	27.79(16.23 to 45.01)	2.39	25.4
Latvia	0.830663516	227.5(183.23 to 280)	1.97	225.53
Lebanon	0.744746351	26.93(15.17 to 45.66)	1.97	24.96
Lesotho	0.510393066	160.09(97.57 to 280.85)	2.4	157.69
Liberia	0.352442452	24.4(9.77 to 40.9)	2.59	21.81
Libya	0.725771399	9.18(5.23 to 15.14)	1.92	7.26
Lithuania	0.856484049	232.64(187.07 to 286.79)	2.03	230.61
Luxembourg	0.884428955	197.48(166.73 to 229.76)	2.03	195.45
Madagascar	0.400246943	69.5(37.87 to 114.54)	2.4	67.1
Malawi	0.384553634	168.86(90.48 to 267.25)	2.41	166.45
Malaysia	0.742523828	38.55(21.07 to 59.65)	1.9	36.65
Maldives	0.650886627	7.76(3.7 to 12.69)	2.4	5.36
Mali	0.268579941	67.85(36.1 to 115.83)	16.62	51.23
Malta	0.801585034	115.99(91.59 to 142.96)	1.82	114.17
Marshall Islands	0.574091128	38.11(21.77 to 63.62)	2.39	35.72
Mauritania	0.4989451	24.6(9.79 to 40.75)	2.39	22.21
Mauritius	0.718260446	21.24(18.55 to 24.07)	1.82	19.42
Mexico	0.664575304	81.57(72.25 to 91.34)	2.39	79.18
Micronesia (Federated States of)	0.587534967	33.95(19.91 to 55.43)	2.4	31.55
Monaco	0.908262831	181.99(101.61 to 263.02)	2.31	179.68
Mongolia	0.617621565	44.44(24.91 to 73.22)	2.39	42.05
Montenegro	0.795800584	165.39(101.74 to 229.53)	2.04	163.35
Morocco	0.562698301	33.71(11.13 to 57.82)	2.4	31.31
Mozambique	0.326462614	99.78(54.84 to 156.06)	2.62	97.16
Myanmar	0.53390084	23.49(14.04 to 36.35)	2.39	21.1
Namibia	0.617564872	281.22(174.36 to 476.13)	2.39	278.83

© 2025 Wang R, et al. *JAMA Dermatol*.

Nauru	0.625177834	38.25(20.1 to 66.63)	2.39	35.86
Nepal	0.433174635	18.94(11.2 to 33.64)	2.39	16.55
Netherlands	0.888464256	337.1(273.21 to 404.45)	1.86	335.24
New Zealand	0.849442499	560.61(452.38 to 680.46)	1.84	558.77
Nicaragua	0.523958472	31.51(16.94 to 46.22)	2.39	29.12
Niger	0.168072774	22.43(9.68 to 36.22)	17.03	5.4
Nigeria	0.503390833	60.46(26.09 to 85.61)	2.4	58.06
Niue	0.72622205	40.57(24.1 to 66.64)	1.77	38.8
North Macedonia	0.750629703	356.43(209.28 to 500.35)	2.3	354.13
Northern Mariana Islands	0.771535213	27.14(16.36 to 41.89)	1.81	25.33
Norway	0.91613281	418.12(367.66 to 463.6)	1.76	416.36
Oman	0.773391602	8.34(4.68 to 14.06)	1.93	6.41
Pakistan	0.504028689	36.75(23.13 to 56.19)	2.39	34.36
Palau	0.754046931	116.28(67.06 to 180.03)	1.87	114.41
Palestine	0.631011665	21.93(13.16 to 31.67)	2.4	19.53
Panama	0.708864828	84.15(63.71 to 103.81)	1.86	82.29
Papua New Guinea	0.417797443	31.44(18.02 to 53.5)	2.39	29.05
Paraguay	0.635718099	85.19(43.6 to 130.74)	2.4	82.79
Peru	0.662054037	76.33(45.16 to 111.42)	2.39	73.94
Philippines	0.651219329	28.47(18.26 to 40.21)	2.39	26.08
Poland	0.812042809	278.57(247.04 to 306.44)	1.96	276.61
Portugal	0.744151851	118.11(96.29 to 142.75)	1.79	116.32
Puerto Rico	0.825525847	48.97(37.68 to 61.66)	1.72	47.25
Qatar	0.846860584	35.63(17.32 to 59.73)	2.33	33.3
Republic of Korea	0.886675267	31.38(11.29 to 45.86)	1.98	29.4
Republic of Moldova	0.732214875	121.45(104.49 to 139.83)	2.38	119.07
Romania	0.768453864	135.99(111.77 to 163.59)	2.03	133.96
Russian Federation	0.808536005	179.25(161.87 to 195.11)	1.82	177.43
Rwanda	0.435588706	91.29(51.38 to 138.06)	2.4	88.89
Saint Kitts and Nevis	0.754987055	29.67(24.36 to 35.38)	1.86	27.81
Saint Lucia	0.672509735	60.91(48.76 to 73.89)	2.4	58.51
Saint Vincent and the Grenadines	0.637195963	68.84(56.25 to 82.38)	2.4	66.44
Samoa	0.593392769	145.41(67.98 to 311.24)	2.4	143.01
San Marino	0.888005474	152.13(82.04 to 242.7)	2	150.13
Sao Tome and Principe	0.505413747	3.02(1.23 to 4.82)	2.39	0.63
Saudi Arabia	0.815143493	5.89(2.92 to 12.06)	2	3.89
Senegal	0.408054193	25.71(11.03 to 42.01)	2.4	23.31
Serbia	0.792416294	217.54(132.24 to 312.7)	1.93	215.61
Seychelles	0.730150775	62.32(33.54 to 91.51)	1.74	60.58
Sierra Leone	0.358665881	23.32(9.38 to 37.82)	2.62	20.7
Singapore	0.856097766	28.02(22.31 to 34.13)	2.3	25.72
Slovakia	0.81061053	238.24(119.64 to 358.83)	1.95	236.29
Slovenia	0.842430731	345.93(272.09 to 426.68)	1.84	344.09

Solomon Islands	0.429360316	32.16(18.66 to 53.62)	2.41	29.75
Somalia	0.077688109	81.89(51.37 to 123.62)	81.89	0
South Africa	0.679626598	130.5(64.14 to 167.49)	2.4	128.1
South Sudan	0.278371125	78.2(46.1 to 118.55)	15.7	62.5
Spain	0.769283698	130.3(104.68 to 157.33)	2.37	127.93
Sri Lanka	0.701534935	18.08(8.88 to 28.42)	2.4	15.68
Sudan	0.541949735	25.98(8.32 to 45.72)	2.39	23.59
Suriname	0.633665739	33.64(18.9 to 52.3)	2.39	31.25
Sweden	0.886880299	309.68(253.31 to 374.28)	1.78	307.9
Switzerland	0.933059111	208.64(162.77 to 256.65)	1.86	206.78
Syrian Arab Republic	0.623004075	18.63(9.89 to 27.23)	2.39	16.24
Taiwan (Province of China)	0.874747053	39.59(31.64 to 47.71)	1.8	37.79
Tajikistan	0.541511187	51.88(31.2 to 75)	2.4	49.48
Thailand	0.682547933	13.8(6.79 to 32.43)	2.4	11.4
Timor-Leste	0.444667619	20(11.81 to 30.07)	2.39	17.61
Togo	0.408533695	27.76(10.22 to 46.22)	2.4	25.36
Tokelau	0.686425621	31.7(17.98 to 54.5)	2.4	29.3
Tonga	0.626349936	34.47(18.95 to 61.7)	2.39	32.08
Trinidad and Tobago	0.768763254	24.93(19.02 to 31.4)	1.88	23.05
Tunisia	0.682432216	14.24(7.87 to 22.97)	2.4	11.84
Turkmenistan	0.712692673	59.29(46.07 to 75.89)	2.39	56.9
Tuvalu	0.682160776	31.88(19.26 to 51.51)	2.39	29.49
Turkey	0.576620529	78.92(37.75 to 117.44)	1.84	77.08
Uganda	0.423261181	169.56(96.68 to 256.67)	2.4	167.16
Ukraine	0.760773913	139.38(101.2 to 182.8)	2.01	137.37
United Arab Emirates	0.849317734	120.74(74.49 to 179.24)	1.99	118.75
United Kingdom	0.859000182	248.72(225.67 to 265.17)	1.81	246.91
United Republic of Tanzania	0.446568273	81.87(44.82 to 127.83)	2.39	79.48
United States of America	0.821830853	232.8(209.18 to 251.76)	1.74	231.06
United States Virgin Islands	0.862448354	74.6(46.63 to 115.45)	1.9	72.7
Uruguay	0.719283445	169.28(136.97 to 204.03)	1.92	167.36
Uzbekistan	0.662621694	42.83(33.53 to 53.66)	2.4	40.43
Vanuatu	0.473100706	39.37(23.1 to 63.01)	2.4	36.97
Venezuela (Bolivarian Republic of)	0.596513059	68.55(52.21 to 87.36)	2.4	66.15
Viet Nam	0.627933721	23.22(13.79 to 34.65)	2.4	20.82
Yemen	0.450376375	30.44(9.28 to 55.41)	2.39	28.05
Zambia	0.505948954	124.96(52.43 to 213.58)	2.4	122.56
Zimbabwe	0.473819486	197.73(113.82 to 293.46)	2.39	195.34

Abbreviations: DALY, disability-adjusted life years; SDI, sociodemographic index.

eTable 10. Frontier analysis based on SDI and DALYs of squamous cell carcinoma in 204 countries and territories.

Location	SDI	DALYs rate in 2021	Frontier DALYs	Effective difference
Afghanistan	0.337199998	1.83(0.56 to 11.99)	0.63	1.19
Albania	0.706849791	156.1(101.05 to 235.91)	0.41	155.69
Algeria	0.659500924	1.97(0.63 to 12.71)	0.4	1.57
American Samoa	0.723727533	104.89(62.37 to 160.85)	0.41	104.48
Andorra	0.869444113	66.22(39.59 to 106.56)	0.4	65.81
Angola	0.453721949	61.63(17.75 to 116.75)	0.46	61.16
Antigua and Barbuda	0.749886887	126.18(107.49 to 147.38)	0.41	125.77
Argentina	0.723122973	103.99(89.37 to 118.58)	0.41	103.58
Armenia	0.701833194	154.56(126.05 to 183.63)	0.41	154.15
Australia	0.844252814	217.14(182.17 to 249.66)	0.4	216.74
Austria	0.853837004	89.28(73.71 to 103.26)	0.4	88.87
Azerbaijan	0.694851274	66.25(38.5 to 108.65)	0.41	65.84
Bahamas	0.805020668	82.77(65.29 to 103.73)	0.4	82.37
Bahrain	0.753043204	48.78(32.17 to 67.04)	0.4	48.37
Bangladesh	0.492420885	49.34(24.76 to 89.27)	0.47	48.87
Barbados	0.746748764	50.18(39.29 to 61.56)	0.4	49.78
Belarus	0.784484711	94.83(77.5 to 114.76)	0.4	94.42
Belgium	0.853654016	72.12(59.82 to 82.97)	0.4	71.71
Belize	0.610229002	110.4(91.01 to 131.25)	0.41	109.99
Benin	0.373486574	15.6(4.8 to 28.22)	0.6	14.99
Bermuda	0.821365422	107.46(85.69 to 133.27)	0.4	107.06
Bhutan	0.473062378	48.93(29.33 to 78.75)	0.46	48.47
Bolivia (Plurinational State of)	0.599010799	161.45(91.35 to 260.18)	0.41	161.05
Bosnia and Herzegovina	0.723077893	73.96(52.96 to 99.09)	0.41	73.55
Botswana	0.642721629	59.02(36.19 to 103.04)	0.4	58.61
Brazil	0.653043887	161.09(140.93 to 174.3)	0.4	160.69
Brunei Darussalam	0.810234367	62.73(41.49 to 93.37)	0.4	62.32
Bulgaria	0.768150939	119.6(101.56 to 138.44)	0.41	119.19
Burkina Faso	0.285118402	16.09(4.62 to 29.53)	0.64	15.46
Burundi	0.289374365	54.54(12.27 to 112.87)	0.64	53.9
Cabo Verde	0.533534539	7.09(3.91 to 12.22)	0.42	6.67
Cambodia	0.473621491	87.86(53.59 to 134.3)	0.46	87.4
Cameroon	0.479691223	18.83(5.68 to 33.14)	0.47	18.36
Canada	0.87317068	99.22(84.27 to 113.18)	0.41	98.82
Central African Republic	0.30916769	53.24(16.32 to 97.05)	0.64	52.61
Chad	0.240436019	17.08(4.81 to 33.49)	0.64	16.45
Chile	0.771514716	127.35(106.76 to 145.04)	0.4	126.95
China	0.72162976	115.39(88.26 to 142.52)	0.4	114.99

Colombia	0.655442913	118.88(95.36 to 144.02)	0.4	118.47
Comoros	0.475978688	63.95(19.51 to 126.25)	0.47	63.49
Congo	0.583075236	69.1(22.36 to 121.26)	0.4	68.7
Cook Islands	0.779109955	34.48(21.07 to 55.45)	0.4	34.07
Costa Rica	0.700340477	117.03(96.01 to 138.74)	0.4	116.62
Croatia	0.425941883	111.99(93.47 to 130.84)	0.4	111.58
Cuba	0.798341027	257.84(213.71 to 302.45)	0.4	257.43
Cyprus	0.668729864	132.79(83.1 to 201.43)	0.41	132.39
Czechia	0.835630545	82.09(69.2 to 95.04)	0.41	81.68
Côte d'Ivoire	0.828450433	18.04(5.27 to 32.71)	0.56	17.49
Democratic People's Republic of Korea	0.569854634	89.06(55.04 to 141.31)	0.4	88.66
Democratic Republic of the Congo	0.383179849	57.48(16.9 to 111.14)	0.56	56.92
Denmark	0.896424204	82.36(69.03 to 95.11)	0.4	81.95
Djibouti	0.487958371	67.62(17.83 to 144.48)	0.46	67.16
Dominica	0.746967185	73.27(47.17 to 109.55)	0.4	72.87
Dominican Republic	0.619388201	161.09(98.43 to 245.34)	0.4	160.69
Ecuador	0.661017053	200.51(161.6 to 247.1)	0.4	200.1
Egypt	0.606787094	34.74(20.17 to 49.98)	0.41	34.33
El Salvador	0.563775188	65.17(45.64 to 90.75)	0.4	64.76
Equatorial Guinea	0.657857456	61.52(18.94 to 111.37)	0.4	61.12
Eritrea	0.403863943	62.27(20.06 to 117)	0.56	61.71
Estonia	0.844917787	111.69(92.09 to 133.31)	0.4	111.28
Eswatini	0.585459713	65.21(35.78 to 110.06)	0.41	64.8
Ethiopia	0.358823295	54.04(13.61 to 100.2)	0.63	53.4
Fiji	0.675051631	46.4(29.41 to 72.55)	0.41	45.99
Finland	0.859831368	56.29(46.36 to 65.29)	0.4	55.88
France	0.838364875	77.49(64.09 to 89.49)	0.4	77.09
Gabon	0.634691393	68.48(21.33 to 118.56)	0.4	68.08
Gambia	0.40971416	19.02(6.25 to 34.13)	0.56	18.46
Georgia	0.732473604	447.81(366.45 to 531.39)	0.4	447.4
Germany	0.902957091	67.23(56.39 to 76.51)	0.41	66.82
Ghana	0.56493039	12.43(7.34 to 20.26)	0.4	12.03
Greece	0.791854408	107.18(90.2 to 122.49)	0.4	106.78
Greenland	0.826210336	35.23(24.47 to 47.58)	0.41	34.83
Grenada	0.668993028	96.05(77.78 to 116.5)	0.41	95.65
Guam	0.803982203	24.51(15.51 to 34.47)	0.41	24.1
Guatemala	0.539972424	123.45(101.69 to 148.42)	0.41	123.04
Guinea	0.336401293	17.65(5.21 to 33.04)	0.64	17.01
Guinea-Bissau	0.353109621	18.76(6.31 to 32.02)	0.63	18.12
Guyana	0.650812335	77.43(57.91 to 100.42)	0.4	77.03
Haiti	0.448278285	99.74(54.12 to 163.39)	0.54	99.2
Honduras	0.513037248	97.54(57.34 to 158.52)	0.47	97.08

Hungary	0.790754768	96.9(80.7 to 112.93)	0.4	96.49
Iceland	0.87636168	35.53(28.84 to 41.77)	0.41	35.12
India	0.575401649	50.42(40.41 to 65.87)	0.41	50.01
Indonesia	0.656868336	79.89(55.48 to 106.05)	0.41	79.48
Iran (Islamic Republic of)	0.697207398	4.73(0.7 to 6.8)	0.41	4.33
Iraq	0.662626231	51.89(31.89 to 81.43)	0.41	51.49
Ireland	0.87375385	128.89(103.76 to 154.84)	0.41	128.48
Israel	0.809011652	113.8(91.91 to 131.95)	0.41	113.39
Italy	0.805773534	78.75(67.03 to 86.49)	0.41	78.34
Jamaica	0.683263064	40.21(30.35 to 51.78)	0.4	39.81
Japan	0.871241813	30.64(25.45 to 33.74)	0.41	30.23
Jordan	0.725307227	45.41(31.87 to 61.31)	0.4	45
Kazakhstan	0.725144495	125.1(104.93 to 146.48)	0.41	124.7
Kenya	0.523768077	59.16(19.03 to 93.07)	0.43	58.74
Kiribati	0.527186583	16.99(10.21 to 26.45)	0.44	16.55
Kuwait	0.846651055	28.42(21.63 to 36.16)	0.41	28.02
Kyrgyzstan	0.603979328	213.54(171.18 to 262.27)	0.41	213.13
Lao People's Democratic Republic	0.489136091	69.62(41.93 to 107.3)	0.47	69.15
Latvia	0.830663516	130.51(109.37 to 152.9)	0.41	130.11
Lebanon	0.744746351	28.89(18.44 to 46.19)	0.4	28.49
Lesotho	0.510393066	70.55(40.12 to 120.22)	0.47	70.08
Liberia	0.352442452	16.3(4.83 to 30.69)	0.63	15.66
Libya	0.725771399	2.31(0.4 to 18.52)	0.4	1.91
Lithuania	0.856484049	95.73(79.48 to 113.18)	0.4	95.33
Luxembourg	0.884428955	72.76(60.25 to 84.96)	0.4	72.35
Madagascar	0.400246943	47.55(13.08 to 91.38)	0.56	46.99
Malawi	0.384553634	61.9(16.17 to 119.83)	0.56	61.34
Malaysia	0.742523828	70.27(46.6 to 102.88)	0.4	69.87
Maldives	0.650886627	19.09(10.22 to 29.99)	0.41	18.69
Mali	0.268579941	18.49(5.64 to 36.54)	0.63	17.85
Malta	0.801585034	67.58(55.88 to 79.22)	0.4	67.17
Marshall Islands	0.574091128	87.97(52.8 to 141.43)	0.4	87.57
Mauritania	0.4989451	18.2(5.71 to 34.54)	0.46	17.74
Mauritius	0.718260446	25.16(21.39 to 29.22)	0.41	24.75
Mexico	0.664575304	113.29(99.55 to 126.68)	0.41	112.88
Micronesia (Federated States of)	0.587534967	87.94(52.99 to 139.77)	0.4	87.53
Monaco	0.908262831	41.3(23.76 to 67.44)	0.41	40.89
Mongolia	0.617621565	79.7(48.22 to 120.72)	0.4	79.29
Montenegro	0.795800584	82.28(60.82 to 109.12)	0.41	81.87
Morocco	0.562698301	1.44(0.4 to 10.27)	0.41	1.03
Mozambique	0.326462614	65.03(15.64 to 128.58)	0.63	64.39
Myanmar	0.53390084	64.18(38.88 to 95.47)	0.43	63.75
Namibia	0.617564872	71.94(43.26 to 122.95)	0.4	71.53

© 2025 Wang R, et al. *JAMA Dermatol.*

Nauru	0.625177834	91.85(50.83 to 153)	0.41	91.45
Nepal	0.433174635	47.91(26.85 to 80.68)	0.55	47.36
Netherlands	0.888464256	63.23(52.8 to 72.83)	0.41	62.82
New Zealand	0.849442499	282.64(234.59 to 329.14)	0.41	282.23
Nicaragua	0.523958472	78.41(51.18 to 116.21)	0.43	77.98
Niger	0.168072774	14.34(4.04 to 28.93)	8.49	5.85
Nigeria	0.503390833	17.18(5.36 to 25.44)	0.45	16.73
Niue	0.72622205	85.63(50.69 to 131.65)	0.4	85.22
North Macedonia	0.750629703	169.5(126.69 to 230.3)	0.41	169.1
Northern Mariana Islands	0.771535213	128.29(70.8 to 197.3)	0.4	127.89
Norway	0.91613281	64.26(54.58 to 71.15)	0.4	63.85
Oman	0.773391602	30.24(18.82 to 47.54)	0.41	29.83
Pakistan	0.504028689	57.86(38.83 to 86.5)	0.46	57.4
Palau	0.754046931	190.64(118.26 to 300.08)	0.41	190.23
Palestine	0.631011665	73.41(38.86 to 107.87)	0.4	73
Panama	0.708864828	54.46(41.16 to 67.35)	0.4	54.06
Papua New Guinea	0.417797443	63.74(31.6 to 112.42)	0.56	63.18
Paraguay	0.635718099	145.26(97.57 to 201.15)	0.4	144.85
Peru	0.662054037	107.5(59.97 to 158.56)	0.41	107.09
Philippines	0.651219329	61.32(48.86 to 78.24)	0.4	60.92
Poland	0.812042809	20.54(17.86 to 22.71)	0.4	20.13
Portugal	0.744151851	108.11(89.75 to 123.62)	0.41	107.71
Puerto Rico	0.825525847	114.59(89.59 to 141.11)	0.41	114.18
Qatar	0.846860584	21.69(11.57 to 36.14)	0.41	21.29
Republic of Korea	0.886675267	58.38(41.99 to 95.6)	0.41	57.97
Republic of Moldova	0.732214875	109.09(91.66 to 127.4)	0.4	108.69
Romania	0.768453864	138.79(116.24 to 163.2)	0.4	138.38
Russian Federation	0.808536005	94.64(84.99 to 102.61)	0.41	94.23
Rwanda	0.435588706	60.91(16.96 to 122.82)	0.53	60.37
Saint Kitts and Nevis	0.754987055	173.23(140.21 to 208.85)	0.4	172.82
Saint Lucia	0.672509735	54.33(43.22 to 66.42)	0.4	53.92
Saint Vincent and the Grenadines	0.637195963	137.31(114.6 to 161.41)	0.4	136.91
Samoa	0.593392769	88.35(52.97 to 141.37)	0.4	87.95
San Marino	0.888005474	38.61(22.52 to 60.7)	0.4	38.21
Sao Tome and Principe	0.505413747	2.78(1.72 to 4.16)	0.46	2.32
Saudi Arabia	0.815143493	18.65(11.91 to 28.19)	0.4	18.25
Senegal	0.408054193	18.71(5.99 to 34.17)	0.56	18.15
Serbia	0.792416294	181.43(134.83 to 238.15)	0.4	181.03
Seychelles	0.730150775	49.19(34.24 to 66.86)	0.41	48.78
Sierra Leone	0.358665881	15.38(4.61 to 28.25)	0.64	14.75
Singapore	0.856097766	27.7(22.64 to 32.22)	0.41	27.29
Slovakia	0.81061053	86.57(64.07 to 122.34)	0.4	86.16
Slovenia	0.842430731	62.37(51.05 to 73.72)	0.41	61.96

© 2025 Wang R, et al. *JAMA Dermatol*.

Solomon Islands	0.429360316	76.21(43.66 to 132.23)	0.56	75.65
Somalia	0.077688109	45.25(10.59 to 94.97)	35.18	10.06
South Africa	0.679626598	114.63(80.94 to 134.15)	0.41	114.22
South Sudan	0.278371125	54.85(13.22 to 112.92)	0.64	54.21
Spain	0.769283698	87.43(72.58 to 100.69)	0.4	87.03
Sri Lanka	0.701534935	78.6(48.85 to 126.33)	0.4	78.19
Sudan	0.541949735	1.49(0.44 to 10.21)	0.41	1.08
Suriname	0.633665739	47.06(28.35 to 72.95)	0.41	46.65
Sweden	0.886880299	53.2(44.25 to 61.42)	0.41	52.79
Switzerland	0.933059111	68.95(56.26 to 80.04)	0.41	68.54
Syrian Arab Republic	0.623004075	0.48(0.34 to 0.65)	0.41	0.08
Taiwan (Province of China)	0.874747053	51.08(42.81 to 59.27)	0.41	50.67
Tajikistan	0.541511187	61.51(38.21 to 93.5)	0.42	61.09
Thailand	0.682547933	81.23(53.07 to 127.83)	0.4	80.82
Timor-Leste	0.444667619	66.34(39.36 to 107.72)	0.48	65.87
Togo	0.408533695	17.09(5.68 to 29.95)	0.56	16.53
Tokelau	0.686425621	85.63(51.41 to 137.9)	0.41	85.22
Tonga	0.626349936	348.46(216.01 to 541.19)	0.4	348.06
Trinidad and Tobago	0.768763254	37.92(28.95 to 48.09)	0.4	37.52
Tunisia	0.682432216	1.62(0.48 to 11.3)	0.41	1.21
Turkmenistan	0.712692673	130.11(100.61 to 169.48)	0.4	129.7
Tuvalu	0.682160776	87.74(53.85 to 140.84)	0.4	87.33
Turkey	0.576620529	93.51(63.17 to 135.29)	0.41	93.1
Uganda	0.423261181	52.64(14.1 to 104.87)	0.55	52.09
Ukraine	0.760773913	86.82(65.17 to 112.21)	0.41	86.41
United Arab Emirates	0.849317734	81.52(52.4 to 122.64)	0.4	81.12
United Kingdom	0.859000182	96.96(86.03 to 103.58)	0.4	96.55
United Republic of Tanzania	0.446568273	54.53(15.12 to 107.09)	0.47	54.05
United States of America	0.821830853	198.09(160.88 to 246.23)	0.41	197.68
United States Virgin Islands	0.862448354	39.82(22.91 to 63.35)	0.41	39.41
Uruguay	0.719283445	85.51(72.88 to 98.16)	0.4	85.1
Uzbekistan	0.662621694	88.56(69.39 to 111.62)	0.4	88.16
Vanuatu	0.473100706	74.75(43.36 to 122.56)	0.46	74.29
Venezuela (Bolivarian Republic of)	0.596513059	204.94(155.69 to 263.39)	0.4	204.54
Viet Nam	0.627933721	80.31(49.35 to 119.14)	0.41	79.9
Yemen	0.450376375	1.54(0.45 to 10.45)	0.5	1.04
Zambia	0.505948954	62.19(17.73 to 112.94)	0.47	61.73
Zimbabwe	0.473819486	72.45(43.92 to 126.19)	0.47	71.99

Abbreviations: DALY, disability-adjusted life years; SDI, sociodemographic index.

© 2025 Wang R, et al. *JAMA Dermatol.*

eTable 11. Frontier analysis based on SDI and DALYs of basal cell carcinoma in 204 countries and territories.

Location	SDI	DALYs rate in 2021	Frontier DALYs	Effective difference
Afghanistan	0.337199998	0.02(0.01 to 0.03)	0	0.02
Albania	0.706849791	0.1(0.03 to 0.23)	0	0.1
Algeria	0.659500924	0.02(0.01 to 0.05)	0	0.02
American Samoa	0.723727533	0(0 to 0)	0	0
Andorra	0.869444113	0.13(0.03 to 0.32)	0	0.13
Angola	0.453721949	0.01(0.01 to 0.03)	0	0.01
Antigua and Barbuda	0.749886887	0.01(0.01 to 0.03)	0	0.01
Argentina	0.723122973	0.09(0.03 to 0.19)	0	0.09
Armenia	0.701833194	0.09(0.03 to 0.18)	0	0.09
Australia	0.844252814	0.1(0.03 to 0.24)	0	0.1
Austria	0.853837004	0.11(0.03 to 0.26)	0	0.11
Azerbaijan	0.694851274	0.08(0.03 to 0.17)	0	0.08
Bahamas	0.805020668	0.01(0.01 to 0.03)	0	0.01
Bahrain	0.753043204	0.02(0.01 to 0.03)	0	0.02
Bangladesh	0.492420885	0(0 to 0)	0	0
Barbados	0.746748764	0.01(0.01 to 0.03)	0	0.01
Belarus	0.784484711	0.08(0.03 to 0.16)	0	0.08
Belgium	0.853654016	0.12(0.03 to 0.27)	0	0.12
Belize	0.610229002	0.02(0.01 to 0.03)	0	0.02
Benin	0.373486574	0.01(0 to 0.02)	0	0.01
Bermuda	0.821365422	0.01(0 to 0.02)	0	0.01
Bhutan	0.473062378	0(0 to 0)	0	0
Bolivia (Plurinational State of)	0.599010799	0.04(0.02 to 0.09)	0	0.04
Bosnia and Herzegovina	0.723077893	0.1(0.03 to 0.22)	0	0.1
Botswana	0.642721629	0.04(0.02 to 0.08)	0	0.04
Brazil	0.653043887	0.09(0.04 to 0.18)	0	0.09
Brunei Darussalam	0.810234367	0.02(0.01 to 0.04)	0	0.02
Bulgaria	0.768150939	0.15(0.04 to 0.35)	0	0.15
Burkina Faso	0.285118402	0.01(0 to 0.02)	0	0.01
Burundi	0.289374365	0.01(0 to 0.02)	0	0.01
Cabo Verde	0.533534539	0.01(0 to 0.02)	0	0.01
Cambodia	0.473621491	0(0 to 0.01)	0	0
Cameroon	0.479691223	0.01(0 to 0.02)	0	0.01
Canada	0.87317068	0.13(0.03 to 0.3)	0	0.13
Central African Republic	0.30916769	0.01(0.01 to 0.03)	0	0.01
Chad	0.240436019	0.01(0 to 0.02)	0	0.01
Chile	0.771514716	0.08(0.03 to 0.18)	0	0.08
China	0.72162976	0.09(0.04 to 0.17)	0	0.09

© 2025 Wang R, et al. *JAMA Dermatol.*

Colombia	0.655442913	0.09(0.03 to 0.2)	0	0.09
Comoros	0.475978688	0.01(0 to 0.02)	0	0.01
Congo	0.583075236	0.01(0.01 to 0.03)	0	0.01
Cook Islands	0.779109955	0(0 to 0)	0	0
Costa Rica	0.700340477	0.11(0.03 to 0.24)	0	0.11
Croatia	0.425941883	0.09(0.03 to 0.18)	0	0.09
Cuba	0.798341027	0.03(0.01 to 0.06)	0	0.03
Cyprus	0.668729864	0.09(0.03 to 0.21)	0	0.09
Czechia	0.835630545	0.13(0.03 to 0.3)	0	0.13
Côte d'Ivoire	0.828450433	0.01(0 to 0.01)	0	0.01
Democratic People's Republic of Korea	0.569854634	0(0 to 0)	0	0
Democratic Republic of the Congo	0.383179849	0.01(0.01 to 0.03)	0	0.01
Denmark	0.896424204	0.14(0.03 to 0.33)	0	0.14
Djibouti	0.487958371	0.01(0 to 0.02)	0	0.01
Dominica	0.746967185	0.01(0.01 to 0.03)	0	0.01
Dominican Republic	0.619388201	0.02(0.01 to 0.03)	0	0.02
Ecuador	0.661017053	0.06(0.02 to 0.12)	0	0.06
Egypt	0.606787094	0.02(0.01 to 0.03)	0	0.02
El Salvador	0.563775188	0.09(0.03 to 0.2)	0	0.09
Equatorial Guinea	0.657857456	0.01(0.01 to 0.03)	0	0.01
Eritrea	0.403863943	0.01(0 to 0.02)	0	0.01
Estonia	0.844917787	0.08(0.03 to 0.17)	0	0.08
Eswatini	0.585459713	0.04(0.02 to 0.07)	0	0.04
Ethiopia	0.358823295	0.01(0 to 0.02)	0	0.01
Fiji	0.675051631	0(0 to 0)	0	0
Finland	0.859831368	0.13(0.03 to 0.28)	0	0.13
France	0.838364875	0.15(0.04 to 0.35)	0	0.15
Gabon	0.634691393	0.01(0.01 to 0.03)	0	0.01
Gambia	0.40971416	0.01(0 to 0.02)	0	0.01
Georgia	0.732473604	0.08(0.03 to 0.18)	0	0.08
Germany	0.902957091	0.11(0.03 to 0.24)	0	0.11
Ghana	0.56493039	0.01(0 to 0.02)	0	0.01
Greece	0.791854408	0.13(0.03 to 0.29)	0	0.13
Greenland	0.826210336	0.18(0.05 to 0.43)	0	0.18
Grenada	0.668993028	0.01(0 to 0.02)	0	0.01
Guam	0.803982203	0(0 to 0)	0	0
Guatemala	0.539972424	0.08(0.03 to 0.18)	0	0.08
Guinea	0.336401293	0.01(0 to 0.02)	0	0.01
Guinea-Bissau	0.353109621	0.01(0 to 0.02)	0	0.01
Guyana	0.650812335	0.01(0 to 0.02)	0	0.01
Haiti	0.448278285	0.01(0.01 to 0.03)	0	0.01
Honduras	0.513037248	0.08(0.03 to 0.18)	0	0.08

© 2025 Wang R, et al. *JAMA Dermatol.*

Hungary	0.790754768	0.1(0.03 to 0.23)	0	0.1
Iceland	0.87636168	0.13(0.03 to 0.32)	0	0.13
India	0.575401649	0(0 to 0.01)	0	0
Indonesia	0.656868336	0(0 to 0.01)	0	0
Iran (Islamic Republic of)	0.697207398	0.03(0.01 to 0.06)	0	0.03
Iraq	0.662626231	0.02(0.01 to 0.03)	0	0.02
Ireland	0.87375385	0.16(0.04 to 0.37)	0	0.16
Israel	0.809011652	0.13(0.03 to 0.3)	0	0.13
Italy	0.805773534	0.15(0.06 to 0.3)	0	0.15
Jamaica	0.683263064	0.02(0.01 to 0.04)	0	0.02
Japan	0.871241813	0.02(0.01 to 0.05)	0	0.02
Jordan	0.725307227	0.02(0.01 to 0.05)	0	0.02
Kazakhstan	0.725144495	0.08(0.03 to 0.17)	0	0.08
Kenya	0.523768077	0.01(0 to 0.02)	0	0.01
Kiribati	0.527186583	0(0 to 0)	0	0
Kuwait	0.846651055	0.02(0.01 to 0.04)	0	0.02
Kyrgyzstan	0.603979328	0.09(0.03 to 0.18)	0	0.09
Lao People's Democratic Republic	0.489136091	0(0 to 0.01)	0	0
Latvia	0.830663516	0.08(0.03 to 0.15)	0	0.07
Lebanon	0.744746351	0.03(0.01 to 0.06)	0	0.03
Lesotho	0.510393066	0.04(0.02 to 0.07)	0	0.04
Liberia	0.352442452	0.01(0 to 0.02)	0	0.01
Libya	0.725771399	0.02(0.01 to 0.03)	0	0.02
Lithuania	0.856484049	0.09(0.03 to 0.21)	0	0.09
Luxembourg	0.884428955	0.13(0.03 to 0.27)	0	0.13
Madagascar	0.400246943	0.01(0 to 0.02)	0	0.01
Malawi	0.384553634	0.01(0 to 0.02)	0	0.01
Malaysia	0.742523828	0.01(0 to 0.02)	0	0.01
Maldives	0.650886627	0(0 to 0.01)	0	0
Mali	0.268579941	0.01(0 to 0.02)	0	0.01
Malta	0.801585034	0.14(0.04 to 0.33)	0	0.14
Marshall Islands	0.574091128	0(0 to 0)	0	0
Mauritania	0.4989451	0.01(0 to 0.02)	0	0.01
Mauritius	0.718260446	0(0 to 0.01)	0	0
Mexico	0.664575304	0.09(0.04 to 0.18)	0	0.09
Micronesia (Federated States of)	0.587534967	0(0 to 0)	0	0
Monaco	0.908262831	0.13(0.04 to 0.29)	0	0.13
Mongolia	0.617621565	0.08(0.03 to 0.16)	0	0.08
Montenegro	0.795800584	0.1(0.03 to 0.22)	0	0.1
Morocco	0.562698301	0.01(0 to 0.02)	0	0.01
Mozambique	0.326462614	0.01(0 to 0.02)	0	0.01
Myanmar	0.53390084	0(0 to 0.01)	0	0
Namibia	0.617564872	0.04(0.02 to 0.08)	0	0.04

© 2025 Wang R, et al. *JAMA Dermatol.*

Nauru	0.625177834	0(0 to 0)	0	0
Nepal	0.433174635	0(0 to 0)	0	0
Netherlands	0.888464256	0.1(0.03 to 0.21)	0	0.1
New Zealand	0.849442499	0.1(0.03 to 0.23)	0	0.1
Nicaragua	0.523958472	0.09(0.03 to 0.21)	0	0.09
Niger	0.168072774	0.01(0 to 0.02)	0.01	0
Nigeria	0.503390833	0.01(0 to 0.02)	0	0.01
Niue	0.72622205	0(0 to 0)	0	0
North Macedonia	0.750629703	0.09(0.03 to 0.2)	0	0.09
Northern Mariana Islands	0.771535213	0(0 to 0)	0	0
Norway	0.91613281	0.13(0.05 to 0.27)	0	0.13
Oman	0.773391602	0.02(0.01 to 0.04)	0	0.02
Pakistan	0.504028689	0(0 to 0)	0	0
Palau	0.754046931	0(0 to 0)	0	0
Palestine	0.631011665	0.02(0.01 to 0.03)	0	0.02
Panama	0.708864828	0.07(0.03 to 0.16)	0	0.07
Papua New Guinea	0.417797443	0(0 to 0)	0	0
Paraguay	0.635718099	0(0 to 0)	0	0
Peru	0.662054037	0.04(0.02 to 0.09)	0	0.04
Philippines	0.651219329	0.01(0 to 0.01)	0	0.01
Poland	0.812042809	0.08(0.03 to 0.16)	0	0.08
Portugal	0.744151851	0.09(0.03 to 0.19)	0	0.09
Puerto Rico	0.825525847	0.02(0.01 to 0.03)	0	0.02
Qatar	0.846860584	0.02(0.01 to 0.03)	0	0.02
Republic of Korea	0.886675267	0.03(0.01 to 0.06)	0	0.03
Republic of Moldova	0.732214875	0.07(0.03 to 0.15)	0	0.07
Romania	0.768453864	0.08(0.03 to 0.17)	0	0.08
Russian Federation	0.808536005	0.07(0.03 to 0.13)	0	0.07
Rwanda	0.435588706	0.01(0 to 0.02)	0	0.01
Saint Kitts and Nevis	0.754987055	0.01(0 to 0.02)	0	0.01
Saint Lucia	0.672509735	0.01(0.01 to 0.03)	0	0.01
Saint Vincent and the Grenadines	0.637195963	0.01(0.01 to 0.03)	0	0.01
Samoa	0.593392769	0(0 to 0)	0	0
San Marino	0.888005474	0.14(0.03 to 0.34)	0	0.14
Sao Tome and Principe	0.505413747	0.01(0 to 0.02)	0	0.01
Saudi Arabia	0.815143493	0.02(0.01 to 0.04)	0	0.02
Senegal	0.408054193	0.01(0.01 to 0.03)	0	0.01
Serbia	0.792416294	0.09(0.03 to 0.19)	0	0.09
Seychelles	0.730150775	0(0 to 0.01)	0	0
Sierra Leone	0.358665881	0.01(0 to 0.02)	0	0.01
Singapore	0.856097766	0.03(0.01 to 0.06)	0	0.03
Slovakia	0.81061053	0.1(0.03 to 0.25)	0	0.1
Slovenia	0.842430731	0.14(0.03 to 0.34)	0	0.14

Solomon Islands	0.429360316	0(0 to 0)	0	0
Somalia	0.077688109	0.01(0 to 0.02)	0.01	0
South Africa	0.679626598	0.06(0.02 to 0.12)	0	0.06
South Sudan	0.278371125	0.01(0 to 0.02)	0	0.01
Spain	0.769283698	0.14(0.03 to 0.35)	0	0.14
Sri Lanka	0.701534935	0(0 to 0.01)	0	0
Sudan	0.541949735	0.02(0.01 to 0.04)	0	0.02
Suriname	0.633665739	0.01(0.01 to 0.03)	0	0.01
Sweden	0.886880299	0.18(0.06 to 0.41)	0	0.18
Switzerland	0.933059111	0.17(0.04 to 0.4)	0	0.17
Syrian Arab Republic	0.623004075	0.02(0.01 to 0.04)	0	0.02
Taiwan (Province of China)	0.874747053	0(0 to 0)	0	0
Tajikistan	0.541511187	0.08(0.03 to 0.17)	0	0.08
Thailand	0.682547933	0.01(0 to 0.02)	0	0.01
Timor-Leste	0.444667619	0(0 to 0.01)	0	0
Togo	0.408533695	0.01(0 to 0.02)	0	0.01
Tokelau	0.686425621	0(0 to 0)	0	0
Tonga	0.626349936	0(0 to 0)	0	0
Trinidad and Tobago	0.768763254	0.01(0.01 to 0.03)	0	0.01
Tunisia	0.682432216	0.02(0.01 to 0.05)	0	0.02
Turkmenistan	0.712692673	0.08(0.03 to 0.18)	0	0.08
Tuvalu	0.682160776	0(0 to 0)	0	0
Turkey	0.576620529	0.04(0.01 to 0.07)	0	0.04
Uganda	0.423261181	0.01(0.01 to 0.03)	0	0.01
Ukraine	0.760773913	0.08(0.03 to 0.15)	0	0.08
United Arab Emirates	0.849317734	0.02(0.01 to 0.04)	0	0.02
United Kingdom	0.859000182	0.14(0.06 to 0.28)	0	0.14
United Republic of Tanzania	0.446568273	0.01(0 to 0.02)	0	0.01
United States of America	0.821830853	1.35(0.62 to 2.62)	0	1.35
United States Virgin Islands	0.862448354	0.01(0.01 to 0.03)	0	0.01
Uruguay	0.719283445	0.1(0.03 to 0.21)	0	0.1
Uzbekistan	0.662621694	0.08(0.03 to 0.15)	0	0.08
Vanuatu	0.473100706	0(0 to 0)	0	0
Venezuela (Bolivarian Republic of)	0.596513059	0.09(0.03 to 0.19)	0	0.09
Viet Nam	0.627933721	0.01(0 to 0.01)	0	0.01
Yemen	0.450376375	0.02(0.01 to 0.03)	0	0.02
Zambia	0.505948954	0.01(0 to 0.02)	0	0.01
Zimbabwe	0.473819486	0.03(0.01 to 0.06)	0	0.03

Abbreviations: DALY, disability-adjusted life years; SDI, sociodemographic index.

© 2025 Wang R, et al. *JAMA Dermatol.*

eFigure 1. Global maps of age-standardised prevalence rate attributable to skin cancer in 2021

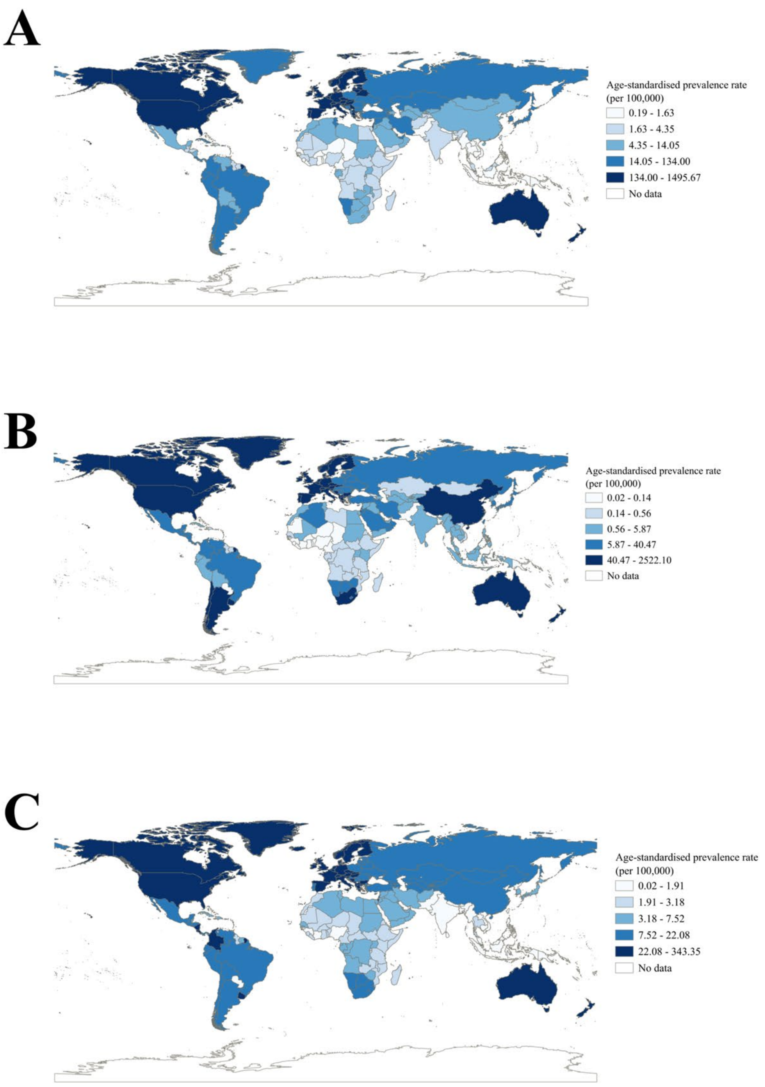

This figure demonstrates age-standardised rate of prevalence attributable to cutaneous melanoma (A), squamous cell carcinoma (B) and basal cell carcinoma (C) across 204 countries and territories in 2021.

© 2025 Wang R, et al. *JAMA Dermatol*.

eFigure 2. Global maps of age-standardised deaths rate attributable to skin cancer in 2021

A

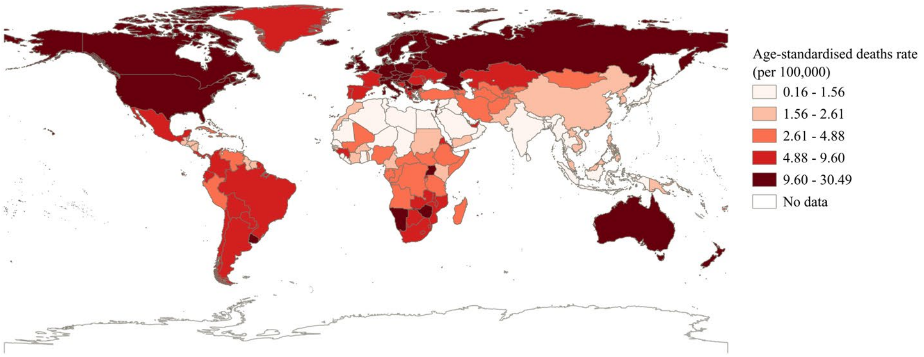

B

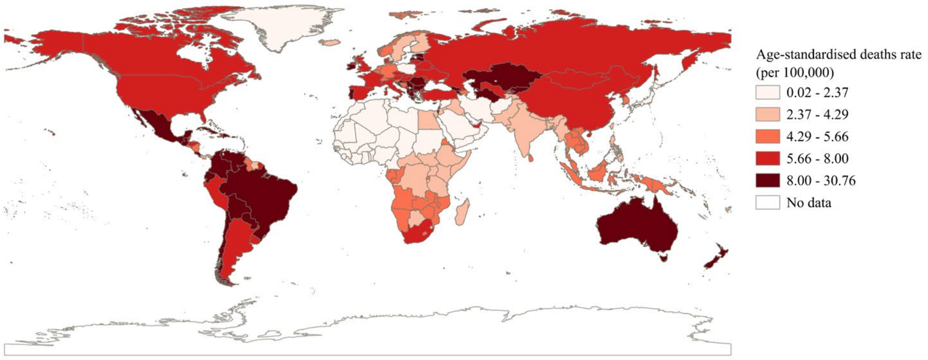

This figure demonstrates age-standardised rate of deaths attributable to cutaneous melanoma (A) and squamous cell carcinoma (B) across 204 countries and territories in 2021.

© 2025 Wang R, et al. *JAMA Dermatol*.

eFigure 3. Global maps of age-standardised disability-adjusted life-years (DALYs) rate attributable to skin cancer in 2021

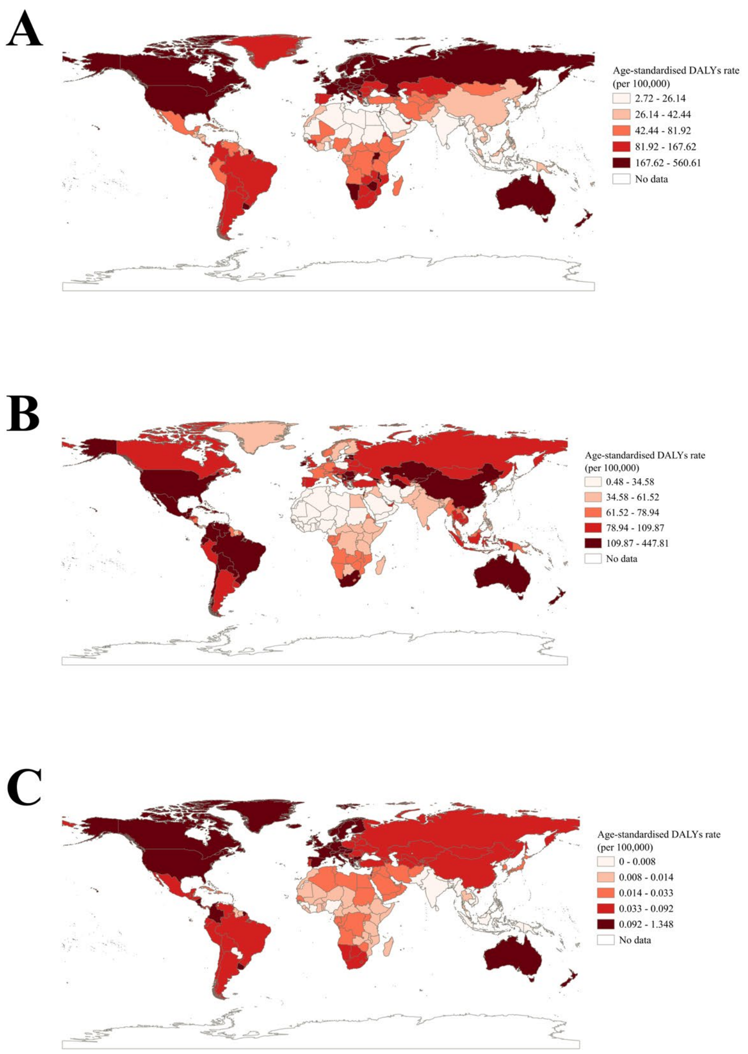

This figure demonstrates age-standardised rate of DALYs attributable to cutaneous melanoma (A), squamous cell carcinoma (B) and basal cell carcinoma (C) across 204 countries and territories in 2021

© 2025 Wang R, et al. *JAMA Dermatol.*

eFigure 4. Decomposition analysis of changes in incidence and DALYs attributable to skin cancer, 1990-2021

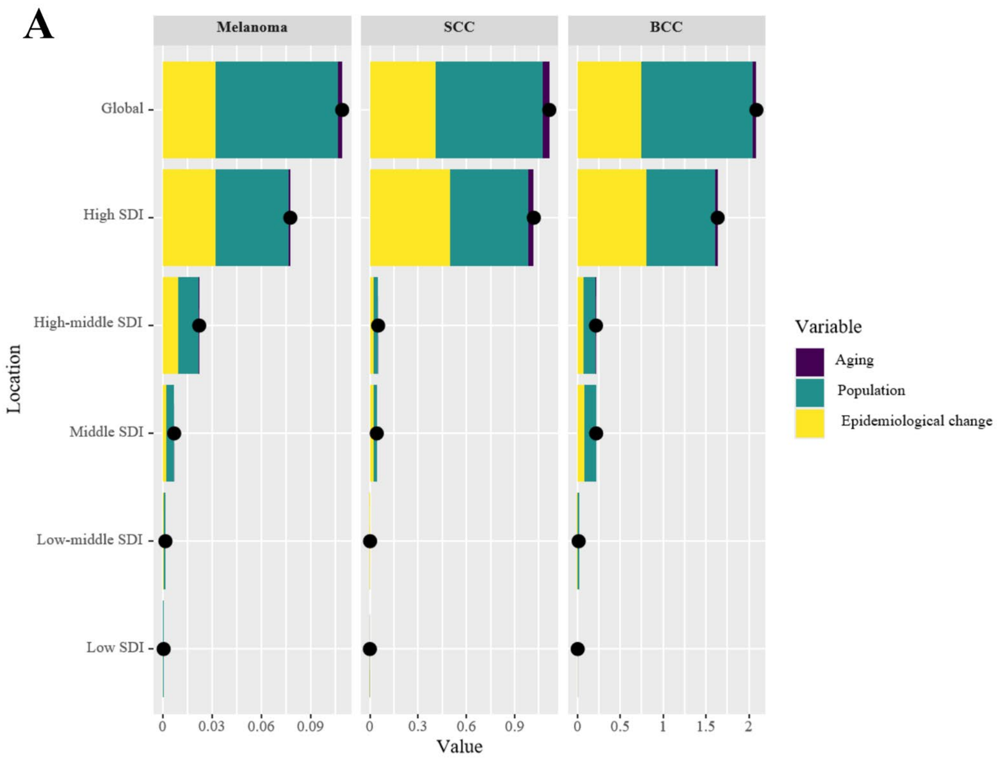

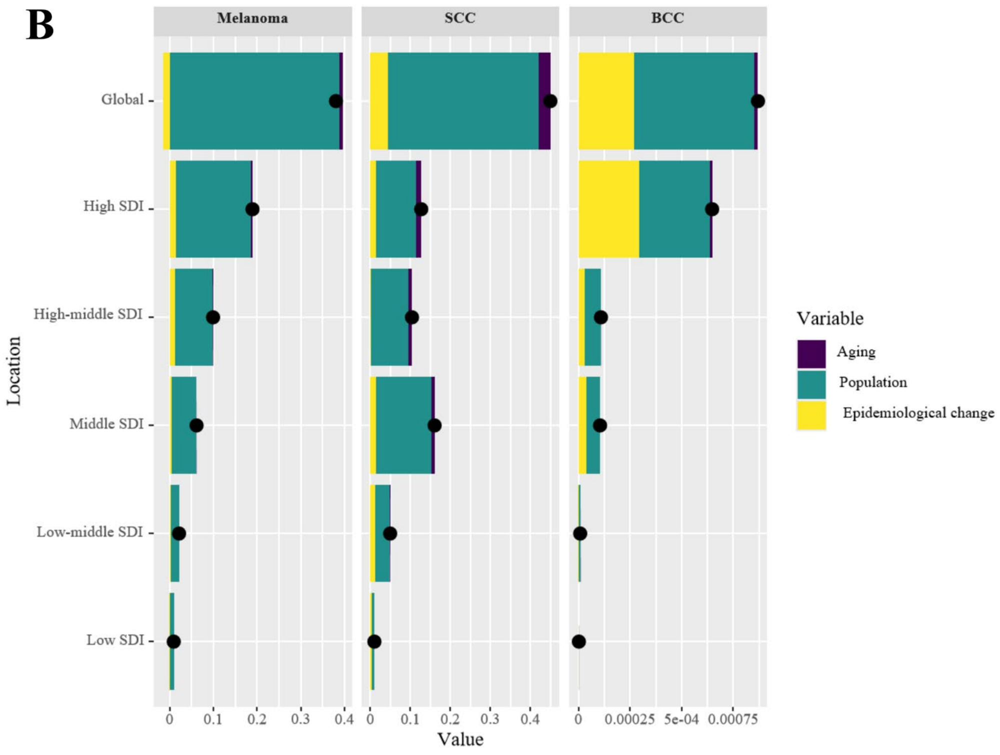

© 2025 Wang R, et al. *JAMA Dermatol*.

This figure demonstrates changes in (A) incidence and (B) DALYs associated with skin cancer according to population-level determinants of aging, population growth, and epidemiological change from 1990 to 2021 at the global level and by SDI quintile. The black dot represents the overall value of change contributed by all three components. For each component, the magnitude of a positive value indicates a corresponding increase in disease metrics attributed to that component, while the magnitude of a negative value indicates a corresponding decrease in disease metrics attributed to the related component.

Abbreviations: DALYs, disability-adjusted life-years; SDI, sociodemographic index.

© 2025 Wang R, et al. *JAMA Dermatol.*

eFigure 5. Inequality analysis of DALYs attributable to skin cancer, 1990-2021

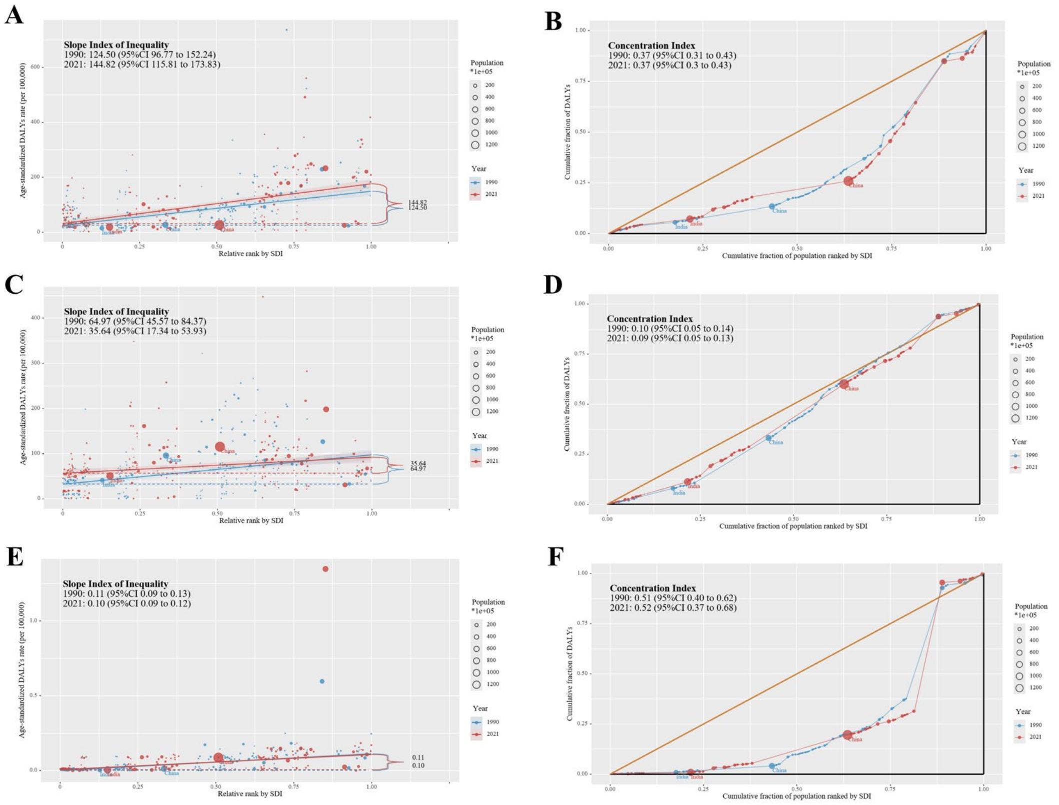

The inequality slope index and concentration index for DALYs of melanoma (A-B), SCC (C-D), and BCC (E-F) worldwide in 1990 and 2021. A, C, and E illustrate the inequality slope index, depicting the relationship between SDI and age-standardised DALYs rates for each condition, with points representing individual countries sized by population. B, D, and F present the concentration index, which quantifies relative inequalities by integrating the area under the Lorenz curve, aligning DALYs distribution with population distribution by SDI. Blue represents data from 1990, and red represents data from 2021.

Abbreviations: DALYs, disability-adjusted life-years; SDI, sociodemographic index; SCC, squamous cell carcinoma; BCC, squamous cell carcinoma.

© 2025 Wang R, et al. *JAMA Dermatol.*

eFigure 6. Frontier analysis based on DALYs attributable to skin cancer and SDI, 1990-2021

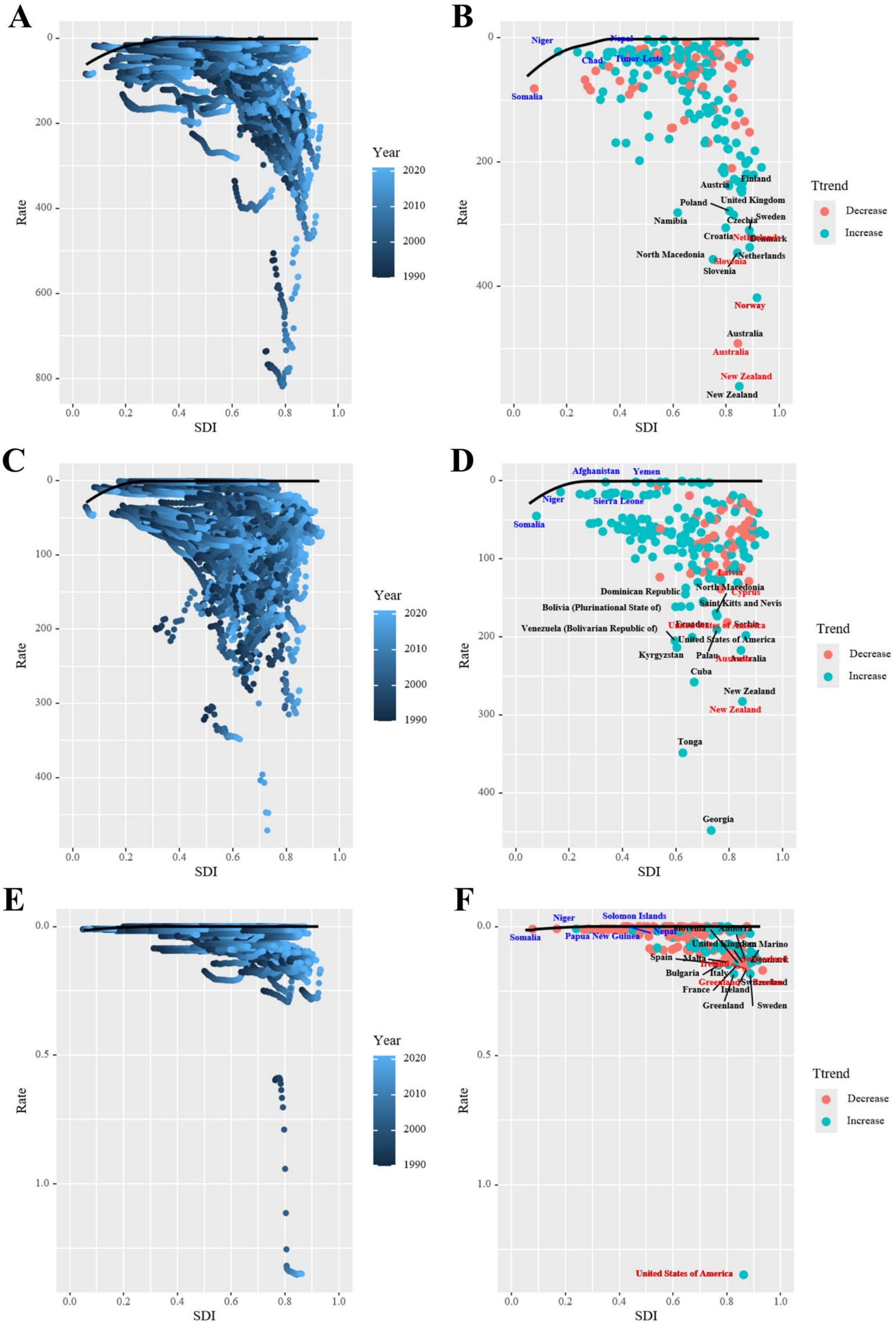

Frontier analysis based on age-standardised DALYs rates of melanoma (A-B), SCC (C-D),

© 2025 Wang R, et al. *JAMA Dermatol*.

and BCC (E-F) and SDI from 1990 to 2021. The color scale ranges from dark blue (1990) to light blue (2021). Panels B, D, and F illustrate the frontier analysis based on age-standardised DALYs rates and SDI in 2021. The solid black line delineates the frontier.

Abbreviations: DALYs, disability-adjusted life-years; SDI, sociodemographic index; SCC, squamous cell carcinoma; BCC, squamous cell carcinoma.

© 2025 Wang R, et al. *JAMA Dermatol*.

eFigure 7. Projected burden of cutaneous melanoma by 2050

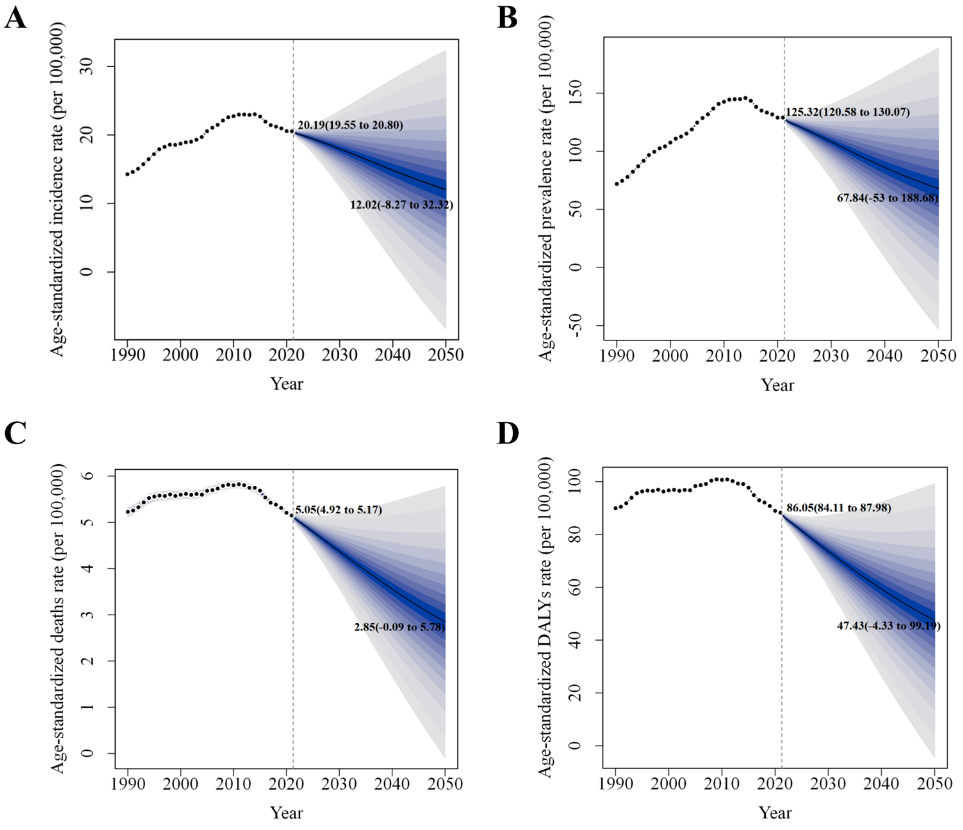

Projected age-standardised rate of (A) incidence, (B) prevalence, (C) deaths, and (D) DALYs attributable to cutaneous melanoma by 2050. The solid line represents the publicly available real-world data from 1990 to 2021 and the dashed line represents the projected data for the period 2022 to 2050. The blue region shows the upper and lower limits of the 95% UI.

Abbreviations: DALYs, disability-adjusted life-years; UI, uncertainty interval.

eFigure 8. Projected burden of squamous cell carcinoma by 2050

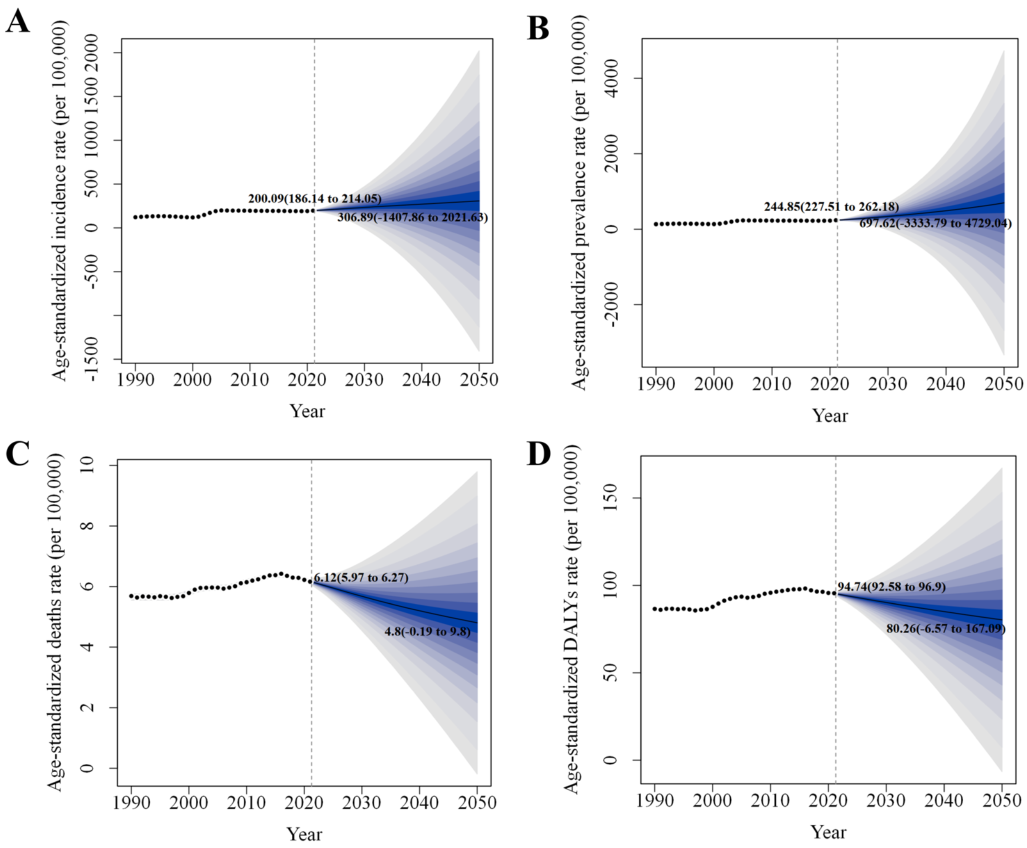

Projected age-standardised rate of (A) incidence, (B) prevalence, (C) deaths, and (D) DALYs attributable to squamous cell carcinoma by 2050. The solid line represents the publicly available real-world data from 1990 to 2021 and the dashed line represents the projected data for the period 2022 to 2050. The blue region shows the upper and lower limits of the 95% UI.

Abbreviations: DALYs, disability-adjusted life-years; UI, uncertainty interval.

© 2025 Wang R, et al. *JAMA Dermatol.*

eFigure 9. Projected burden of basal cell carcinoma by 2050

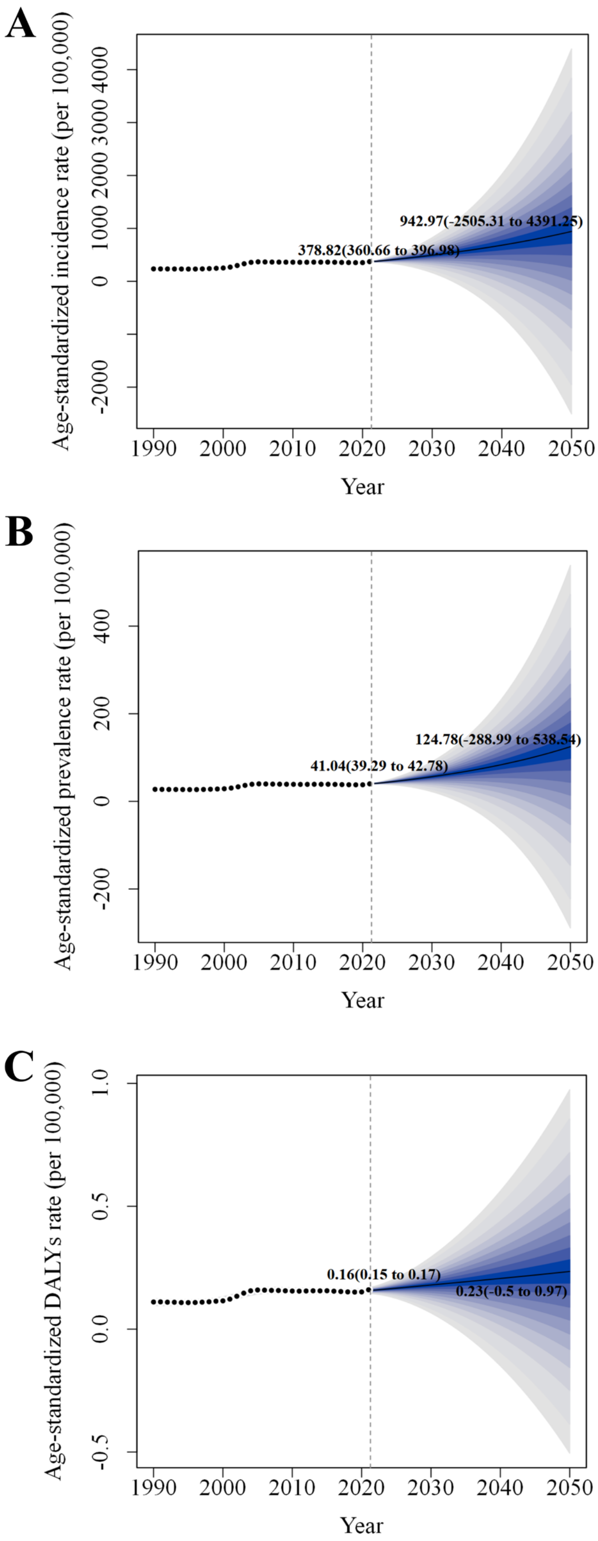

Projected age-standardised rate of (A) incidence, (B) prevalence, and (C) DALYs attributable to basal cell carcinoma by 2050. The solid line represents the publicly available real-world

© 2025 Wang R, et al. *JAMA Dermatol.*

data from 1990 to 2021 and the dashed line represents the projected data for the period 2022 to 2050. The blue region shows the upper and lower limits of the 95% UI.

Abbreviations: DALYs, disability-adjusted life-years; UI, uncertainty interval.

© 2025 Wang R, et al. *JAMA Dermatol*.
