# Supplementary material for: Burden of Skin Cancer in Older Adults From 1990 to 2021 and Modelled Projection to 2050
Source: JAMA Dermatol. 2025 May 21;161(7):715–22. doi: 10.1001/jamadermatol.2025.1276 (PMC12096324; doi:10.1001/jamadermatol.2025.1276)
Supplement: Supplement 2. — Data Sharing Statement [file jamadermatol-e251276-s002.pdf]

Data Sharing Statement

Wang. Burden of Skin Cancer in Older Adults From 1990 to 2021 and Modelled Projection to 2050. *JAMA Dermatol*. Published May 21, 2025. doi:10.1001/jamadermatol.2025.1276

Data
Data available: No

Additional Information
Explanation for why data not available: The analyzed datasets during the current study are available from the corresponding author on reasonable request.
